# RhoA signaling increases mitophagy and protects cardiomyocytes against ischemia by stabilizing PINK1 protein and recruiting Parkin to mitochondria

**DOI:** 10.1038/s41418-022-01032-w

**Published:** 2022-06-27

**Authors:** Michelle Tu, Valerie P. Tan, Justin D. Yu, Raghav Tripathi, Zahna Bigham, Melissa Barlow, Jeffrey M. Smith, Joan Heller Brown, Shigeki Miyamoto

**Affiliations:** 1 Department of Pharmacology, University of California, San Diego, 9500 Gilman Drive, La Jolla, CA 92093-0636 USA; 2 Present Address: Sanford Burnham Prebys Medical Discovery Institute, 10901 North Torrey Pines Road, La Jolla, CA 92037 USA

**Keywords:** Macroautophagy, Cardiovascular diseases

## Abstract

Mitophagy, a mitochondria-specific form of autophagy, removes dysfunctional mitochondria and is hence an essential process contributing to mitochondrial quality control. PTEN-induced kinase 1 (PINK1) and the E3 ubiquitin ligase Parkin are critical molecules involved in stress-induced mitophagy, but the intracellular signaling mechanisms by which this pathway is regulated are unclear. We tested the hypothesis that signaling through RhoA, a small GTPase, induces mitophagy via modulation of the PINK1/Parkin pathway as a protective mechanism against ischemic stress. We demonstrate that expression of constitutively active RhoA as well as sphingosine-1-phosphate induced activation of endogenous RhoA in cardiomyocytes result in an accumulation of PINK1 at mitochondria. This is accompanied by translocation of Parkin to mitochondria and ubiquitination of mitochondrial proteins leading to recognition of mitochondria by autophagosomes and their lysosomal degradation. Expression of RhoA in cardiomyocytes confers protection against ischemia, and this cardioprotection is attenuated by siRNA-mediated PINK1 knockdown. In vivo myocardial infarction elicits increases in mitochondrial PINK1, Parkin, and ubiquitinated mitochondrial proteins. AAV9-mediated RhoA expression potentiates these responses and a concurrent decrease in infarct size is observed. Interestingly, induction of mitochondrial PINK1 accumulation in response to RhoA signaling is neither mediated through its transcriptional upregulation nor dependent on depolarization of the mitochondrial membrane, the canonical mechanism for PINK1 accumulation. Instead, our results reveal that RhoA signaling inhibits PINK1 cleavage, thereby stabilizing PINK1 protein at mitochondria. We further show that active RhoA localizes at mitochondria and interacts with PINK1, and that the mitochondrial localization of RhoA is regulated by its downstream effector protein kinase D. These findings demonstrate that RhoA activation engages a unique mechanism to regulate PINK1 accumulation, induce mitophagy and protect against ischemic stress, and implicates regulation of RhoA signaling as a potential strategy to enhance mitophagy and confer protection under stress conditions.

## Introduction

Mitochondria are the main site of ATP generation in the heart, but under stress conditions they become damaged and contribute to development of tissue dysfunction and disease. This occurs through generation of reactive oxygen species as well as other molecules such as cytochrome c released from damaged mitochondria, inducing necrotic and apoptotic cell death [1, 2]. Thus, prevention of mitochondrial deterioration and removal of damaged mitochondria are critical for cell survival.

Autophagy is an evolutionarily conserved catabolic process that is rapidly induced in response to stress [3–5]. Cytoplasmic components and damaged organelles are engulfed by autophagosomes which subsequently fuse with lysosomes and are degraded by lysosomal enzymes [6–14]. There is also a selective form of autophagy, termed mitophagy, which specifically eliminates damaged mitochondria, thereby maintaining mitochondrial quality [4, 15–19]. An established mechanism of tagging damaged mitochondria for removal is the PTEN-induced kinase 1 (PINK1)/Parkin pathway. In healthy cells, PINK1, a mitochondrial serine/threonine kinase, is constitutively cleaved and released from mitochondria to the cytosol where it undergoes proteasomal degradation. Under stress conditions, dissipation of the mitochondrial membrane potential leads to inhibition of PINK1 cleavage and accumulation of full-length PINK1 at the outer mitochondrial membrane [17, 20–22]. Full-length PINK1 recruits Parkin, an E3 ubiquitin ligase, which subsequently ubiquitinates and tags damaged mitochondria for selective removal [21–24].

In the heart, acute ischemia caused by myocardial infarction (MI) progresses to irreversible damage, and ischemic heart disease is the most common cause of heart failure [25]. It has been shown that cardiac damage induced by ischemic stress is exacerbated in PINK1 knockout (KO) and Parkin KO mouse hearts [26, 27]. Protective roles of the PINK1/Parkin pathway against ischemic stress have also been demonstrated in other tissues including liver, kidney and brain [15, 28–31]. Depolarization of the mitochondrial membrane potential and activation of the canonical PINK1/Parkin pathway are late-stage events leading to mitophagy which occur just before cell death [25, 32]. The discovery of additional intracellular mechanisms that enhance mitophagy at earlier stages would be important for prolonging cell survival during stress.

RhoA is a small G-protein and intracellular signal transducer that modulates a range of cellular processes including cell proliferation and cell survival [33–38]. RhoA is activated in response to stimulation of various G protein-coupled receptors (GPCRs) including those for sphingosine-1-phosphate (S1P), a cardioprotective ligand generated and released in response to injury [39–41]. We and others have demonstrated that RhoA signaling provides cardioprotection against oxidative stress through inhibition of mitochondrial death pathways [35, 37, 42–47]. However, it has not been determined whether RhoA signals also regulate mitochondrial quality control through modulation of mitophagy. Here, using pharmacological and genetic approaches, we provide evidence that activation of RhoA stabilizes PINK1 protein at mitochondria, induces mitophagy, and promotes cardiomyocyte survival against ischemia both in vitro and in vivo.

## Results

### RhoA activation increases levels of exogenously expressed PINK1 and Parkin at mitochondria

**Fig. 1:**
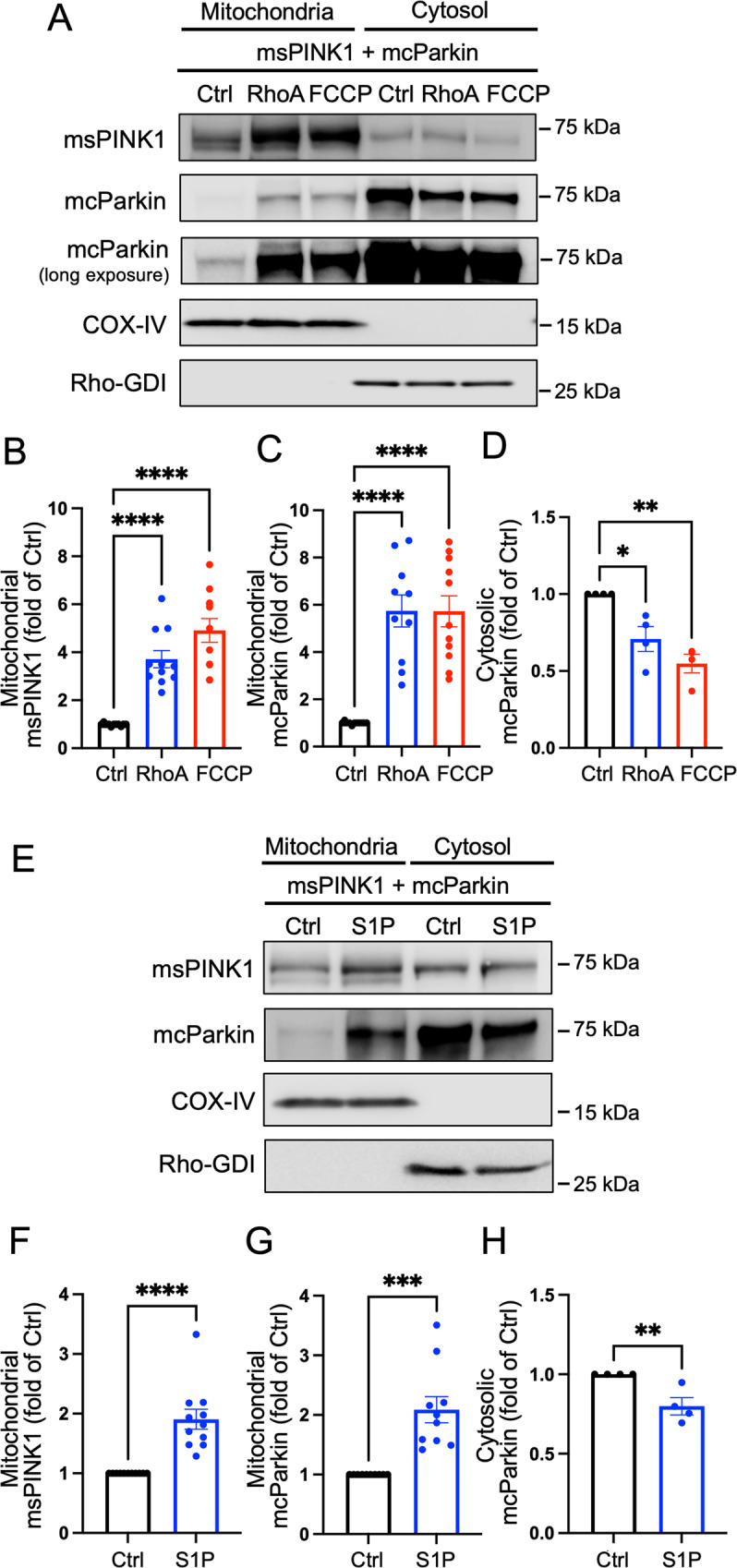
RhoA activation increases exogenously expressed PINK1 and Parkin at mitochondria. miniSOG-tagged PINK1 (msPINK1) and mCherry-tagged Parkin (mcParkin) were adenovirally expressed at 10 MOI (multiplicity of infection) in neonatal rat ventricular myocytes (NRVMs). **A**, **B**, **C**, **D** GFP (Ctrl) or HA-tagged constitutively active RhoA (L63RhoA) were co-expressed at 30 MOI with msPINK1 and mcParkin for 16 h. Mitochondrial and cytosolic fractions were isolated and subjected for Western blot (WB). FCCP (20 μM for 2 h) was used as a positive control. *n* = 4–11, *p < 0.05, **p < 0.01, ****p < 0.0001. **E**, **F**, **G**, **H** Cells were treated with S1P at 1 μM for 1 h and mitochondrial and cytosolic fractions were isolated for WB analysis. *n* = 4–10. **p < 0.01, ***p < 0.001, ****p < 0.0001. COX-IV was used as a mitochondrial marker and loading control, and Rho-GDI was used as a cytosolic marker and loading control.

To explore a possible role for RhoA in regulation of PINK1 and Parkin association with mitochondria, a hemagglutinin (HA)-tagged active mutant of RhoA (L63RhoA) was adenovirally co-expressed in neonatal rat ventricular myocytes (NRVMs) along with miniSOG-tagged PINK1 (msPINK1) and mCherry-tagged Parkin (mcParkin). Mitochondrial and cytosolic fractions were isolated and subjected to Western blotting (WB) for PINK1 and Parkin. As expected based on previous studies [20, 21, 23, 48], a mitochondrial uncoupler FCCP induced robust increases in PINK1 in the mitochondrial fractions (Fig. 1A, B). Remarkably, RhoA expression also significantly increased levels of PINK1 in the mitochondrial fractions compared to adenovirally expressed GFP control (Ctrl) (Fig. 1A, B). Both FCCP treatment and RhoA expression also induced increases in Parkin in the mitochondrial fractions with concomitant decreases in Parkin in the cytosolic fractions (Fig. 1A, C, D), suggesting recruitment of Parkin to the mitochondria in response to increased PINK1. In addition to examining responses to expression of active RhoA, we treated cells with S1P, a GPCR agonist that activates RhoA [46, 47]. S1P treatment significantly increased mitochondrial levels of PINK1, leading to recruitment of Parkin as indicated by increased mitochondrial and decreased cytosolic Parkin (Fig. 1E–H).

### RhoA increases endogenous PINK1 at mitochondria

**Fig. 2:**
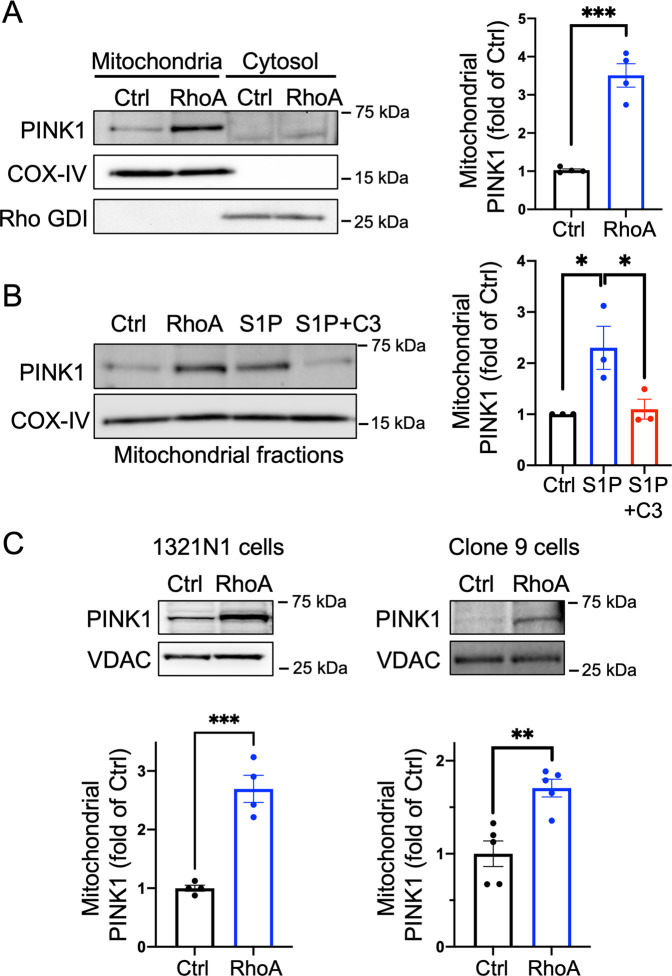
RhoA activation leads to increases in endogenous PINK1 at mitochondria. **A** Cells were infected with GFP (Ctrl) and RhoA adenovirus for 16 h and mitochondrial and cytosolic fractions were isolated for WB analysis. Endogenous PINK1 levels in mitochondrial fractions were quantified. COX-IV (mitochondrial marker) and Rho-GDI (cytosolic marker) were used as loading controls. *n* = 4, ***p < 0.001. **B** Cells were treated with 1 μM S1P for 1 h and mitochondrial fractions were isolated for WB. For RhoA inhibition studies, NRVMs were pretreated with 1.5 μg/ml C3 exoenzyme for 6 h prior to S1P stimulation. *n* = 3, *p < 0.05. **C** RhoA was adenovirally expressed in 1321N1 human astrocytoma cells (*n* = 4) and in Clone 9 rat hepatocyte cells (*n* = 5), and the levels of endogenous PINK1 in mitochondrial fractions were assessed. VDAC was used as a mitochondrial marker and loading control. **p < 0.01, ***p < 0.001.

To examine whether endogenous PINK1 levels at mitochondria were also increased by activation of RhoA, cytosolic and mitochondrial fractions were isolated from cells expressing GFP (Ctrl) or RhoA and subjected to WB. RhoA significantly increased the level of PINK1 in the mitochondrial fractions without affecting cytosolic PINK1 (Fig. 2A). S1P treatment also increased endogenous PINK1 levels in the mitochondrial fractions and this response was RhoA-dependent as indicated by its attenuation through functional inhibition of RhoA with C3 exoenzyme (Fig. 2B). We further determined that activated RhoA has similar effects in non-cardiac cells, specifically 1321N1 human astrocytoma cells and Clone 9 rat liver cells. In both cell types, RhoA expression also significantly increased mitochondrial PINK1 (Fig. 2C), suggesting that this may be a global rather than cell type-specific phenomenon.

### RhoA induces mitophagy

**Fig. 3:**
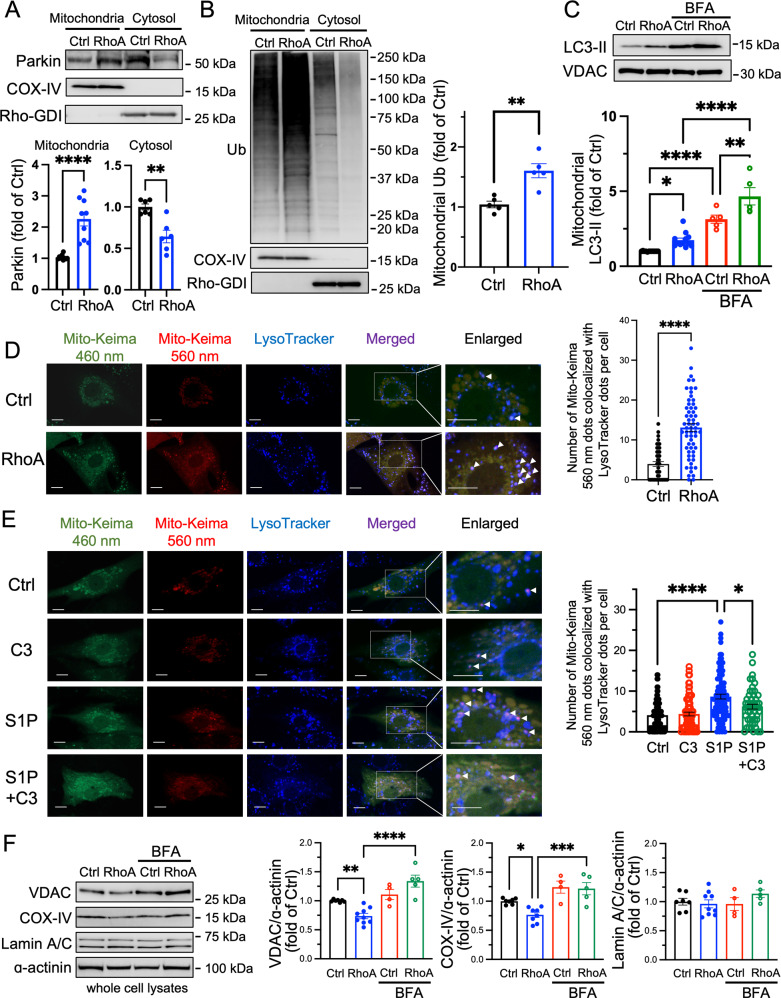
RhoA activation increases mitophagy. **A**–**C** Cells were infected with GFP (Ctrl) and RhoA adenovirus for 24 h and mitochondrial and cytosolic fractions were isolated for WB analysis. The lysosome inhibitor Bafilomycin A1 (BFA) was used at 50 nM. COX-IV and VDAC (mitochondrial markers) and Rho-GDI (cytosolic marker) were used as loading controls. *n* = 5–9, *p < 0.05, **p < 0.01, ***p < 0.001, ****p < 0.0001. **D**, **E** Mitophagy was assessed by Mito-Keima. Mito-Keima was adenovirally co-expressed at 20 MOI in NRVMs with GFP (Ctrl) or RhoA for 32 h or treated with S1P at 1 μM for 1-2 h. LysoTracker Blue (250 nM) was loaded onto cells for 2 h prior to visualization. Merged images show all three colors and arrowheads to the purple dots indicate co-localization of Mito-Keima-560 nm fluorescence and LysoTracker Blue fluorescence signals. Scale bars: 10 μm. *n* > 40 from four independent experiments; *p < 0.05, ****p < 0.0001. **F** After 36 h infection of RhoA or GFP adenovirus in the absence or presence of BFA (50 nM), cells were collected and subjected to WB for VDAC, COX-IV, Lamin A/C and α-actinin, and band intensities were quantitated. *n* = 4–8, *p < 0.05, **p < 0.01, ***p < 0.001, ****p < 0.0001.

During the process of mitophagy, PINK1 recruits Parkin to mitochondria following which Parkin catalyzes the ubiquitination of mitochondrial proteins. Ubiquitinated mitochondria are recognized by LC3-II on autophagosomes [4, 15–19], culminating in degradation of the tagged mitochondria in lysosomes. RhoA induced increase in mitochondrial and decrease in cytosolic Parkin, and this was associated with a marked increase in mitochondrial protein ubiquitination without affecting cytosolic protein ubiquitination (Fig. 3A, B). The level of LC3-II in the mitochondrial fraction, which is indicative of the recognition of mitochondria by autophagosomes, was also significantly increased by RhoA. (Fig. 3C). This increase was not abolished by the lysosome inhibitor Bafilomycin A1 (BFA), supporting induction of mitophagy rather than changes in lysosomal clearance in response to RhoA. To directly assess mitophagy, we used mitochondrial matrix-targeted Keima (Mito-Keima), a pH-sensitive, lysosomal protease-resistant fluorescent probe together with LysoTracker blue, a fluorescent stain for lysosomes as described in our previous work [48]. In the acidic environment of the lysosome, Mito-Keima excitation shifts from 460 nm to 560 nm, thus colocalization of Mito-Keima 560 nm with LysoTracker signals, visualized as purple puncta, is indicative of mitochondria in the lysosome [48–50]. Remarkably, RhoA expression for 32 h increased the number of Mito-Keima 560 nm-LysoTracker double-positive puncta (Fig. 3D; purple dots indicated by arrowheads). Importantly, S1P treatment also increased the number of double-positive puncta, and this response was diminished by inhibition of RhoA with C3 exoenzyme (Fig. 3E).

To determine whether mitochondrial clearance is enhanced as a result of mitophagy induced by RhoA expression, the levels of mitochondrial proteins in whole cell lysates were measured. Following RhoA expression for 36 h, the protein levels of mitochondrial proteins VDAC and COX-IV, expressed relative to α-actinin, were significantly decreased compared to control (Fig. 3F). The RhoA-induced decrease in VDAC and COX-IV levels was reversed by BFA. Expression levels of Lamin A/C, a nuclear protein, were unaffected by RhoA or BFA. Taken together, our data provide evidence for a RhoA-triggered lysosome-dependent mechanism for the selective clearance of mitochondrial proteins. Together, these results suggest that in response to RhoA activation, ubiquitinated mitochondria are recognized by autophagosomes, delivered to lysosomes, and undergo degradation.

### RhoA and cardioprotection

**Fig. 4:**
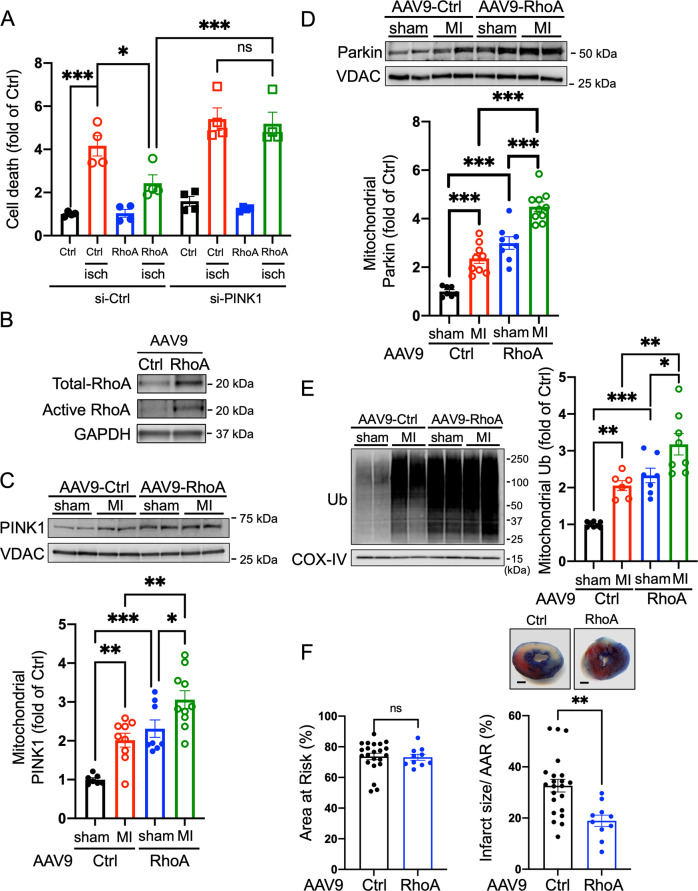
RhoA activation confers protection in vitro and in vivo. **A** NRVMs were transfected with control siRNA (si-Ctrl) or PINK1 siRNA (si-PINK1) followed by infection with GFP (Ctrl) or RhoA adenovirus. Cells were then subjected to simulated ischemia (isch) for 16 h and apoptotic cell death was assessed. *n* = 4, *p < 0.05, ***p < 0.001. **B** AAV9-GFP (Ctrl) or AAV9-L63RhoA (0.3 × 10^11^vp/mouse) was injected into mice via the tail vein and allowed to express for 2 wk, after which total and activated RhoA were assessed in whole heart homogenates by WB. GAPDH was used as a loading control. **C**–**E** Mice were injected via the tail vein with AAV9-GFP (Ctrl) or AAV9-RhoA. After allowing constructs to express for 2 wk, mice were subjected to sham surgery or MI surgery via LAD ligation. Mice were euthanized 1 h following the procedures, and mitochondrial fractions were isolated from the hearts for WB to assess levels of PINK1, Parkin and ubiquitinated proteins. VDAC and COX-IV were used as mitochondrial markers and served as loading controls. *n* = 6–10; *p < 0.05, **p < 0.01, ***p < 0.001. **F** Mice injected with AAV9-GFP (Ctrl) or AAV9-RhoA for 2 wk were subjected to sham surgery or MI surgery. Mice were sacrificed 2 h after, and hearts were perfused with Evans Blue and stained with triphenyltetrazolium chloride (TTC) for morphometric determination of Area at Risk (AAR) and infarct size. Scale bar: 1 mm. *n* > 9, **p < 0.01.

To determine whether mitophagy induced by RhoA activation confers protection, cardiomyocytes were subjected to simulated ischemia and apoptotic cell death was assessed as previously reported [48, 51, 52]. Cell death induced by ischemia was significantly diminished by RhoA expression and importantly, this protection was reversed by siRNA-mediated PINK1 knockdown (Fig. 4A). These results support our previous findings that RhoA is cardioprotective [35, 45] and further suggest that PINK1-mediated mitophagy plays a critical role in RhoA-mediated protection against ischemic stress.

We have previously used adeno-associated virus serotype 9 (AAV9, the most cardiotropic subtype) driven by the cardiac-specific MLC2v promoter to achieve cardiac-specific expression of genes of interest in vivo via tail vein injection [42, 48]. AAV9-mediated RhoA expression and activity in the heart were verified in whole heart homogenates (Fig. 4B). The effect of RhoA activation on the response to ischemia was then examined. Mice were injected with AAV9-GFP or AAV9-RhoA and then 2 wk later subjected to sham surgery or myocardial infarction (MI) surgery via ligation of the left anterior descending artery (LAD) to induce regional ischemia. Consistent with previous reports [29, 53–55], mitochondrial PINK1 levels were increased by ischemia in AAV9-GFP mice; notably this was further increased by expression of AAV9-RhoA (Fig. 4C). Similarly, increases in mitochondrial Parkin levels and ubiquitination of mitochondrial proteins induced by ischemia were also enhanced by RhoA expression (Fig. 4D, E). Finally, we assessed cardiac damage 2 h following sham or LAD ligation procedures, a timepoint that has been demonstrated in previous studies including our own to induce significant MI development in mice [48, 56, 57]. The area at risk (AAR) was not different between AAV9-GFP and AAV9-RhoA injected mice subjected to LAD ligation, indicating that the area of myocardium subjected to ischemia was similar in both groups; however, infarct size per AAR was significantly reduced in the heart expressing RhoA (Fig. 4F), implicating a cardioprotective role for RhoA.

### RhoA does not induce mitochondrial membrane depolarization or transcriptionally upregulate PINK1 expression but impedes PINK1 protein degradation

**Fig. 5:**
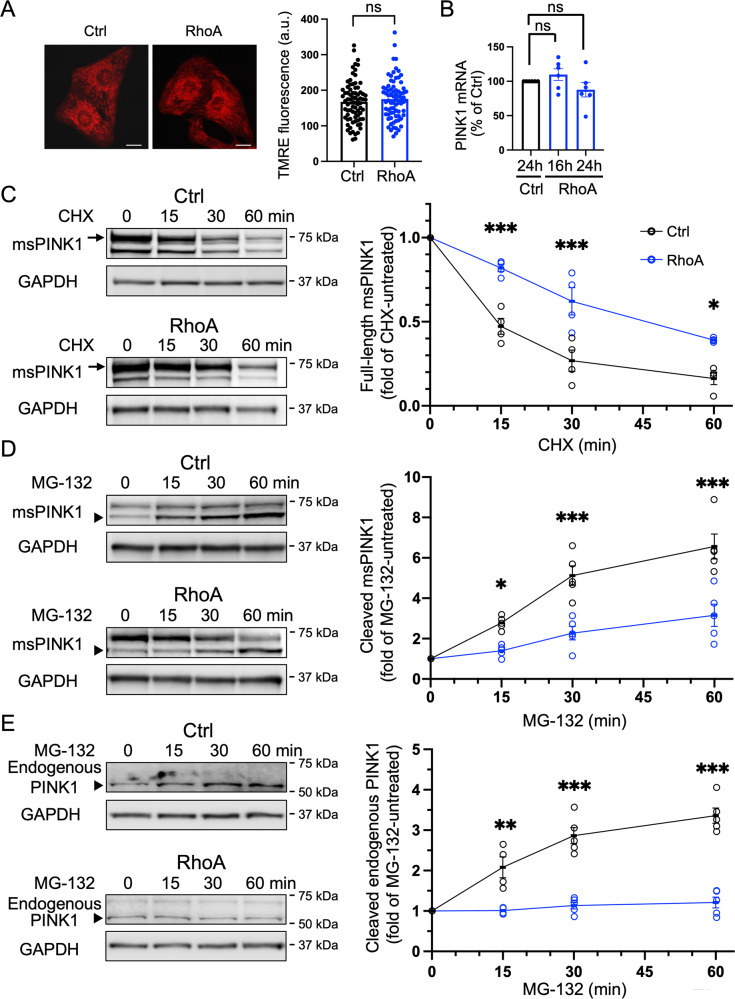
RhoA does not depolarize mitochondrial membrane potential or upregulate PINK1 mRNA but inhibits PINK1 protein degradation in cardiomyocytes. **A** Mitochondrial membrane potential in NRVMs adenovirally infected with GFP (Ctrl) or RhoA was measured using TMRE, visualized by confocal microscopy, and cellular TMRE fluorescence intensity was quantified. Scale bars: 10 μm. *n* > 50 from three independent experiments; ns, not significant. **B** NRVMs were infected with GFP (Ctrl) or RhoA adenovirus for 16 and 24 h and PINK1 mRNA levels were assessed by qPCR. *n* = 6; ns, not significant. **C** GFP (Ctrl) or RhoA was co-expressed with msPINK1 for 16 h, and then treated with cycloheximide (CHX; 100 μg/ml) for the indicated times. Whole cell lysates were subjected to WB for PINK1 (msPINK1) and GAPDH (loading control). Arrows denote full length msPINK1, while lower bands are cleaved msPINK1. *n* = 5; *p < 0.05, ***p < 0.001. **D** GFP (Ctrl) or RhoA was co-expressed with msPINK1 for 16 h, and then treated with MG-132 (50 μM) for the indicated times. Whole cell lysates were subjected to WB for msPINK1 and GAPDH (loading control). Arrowheads denote cleaved msPINK1. *n* = 5, *p < 0.05, ***p < 0.001. **E** Cells expressing GFP (Ctrl) or RhoA were treated with MG-132 (50 μM) for the indicated times. Whole cell lysates were subjected to WB for PINK1 and GAPDH (loading control). Arrowheads denote cleaved endogenous PINK1. *n* = 4-5, **p < 0.01, ***p < 0.001.

Depolarization of the mitochondrial membrane has been shown to inhibit PINK1 cleavage and subsequent degradation, resulting in PINK1 accumulation at mitochondria. To determine whether the observed effects of RhoA activation result from changes in mitochondrial membrane potential, we used tetramethylrhodamine ethyl ester (TMRE), a mitochondrial membrane potential indicator. No significant differences in cellular TMRE fluorescence were observed in GFP versus RhoA expressing cardiomyocytes (Fig. 5A). We next determined if RhoA was regulating PINK1 expression at the transcriptional level since RhoA is known to play a critical role in transcriptional regulation [58–60]. However, qPCR analysis of PINK1 mRNA isolated from cells expressing GFP versus RhoA showed no significant differences (Fig. 5B). Next, we tested the possibility that RhoA regulates PINK1 protein degradation and hence its stability. Using cycloheximide to inhibit protein synthesis, we compared the rates of degradation of exogenously expressed msPINK1 protein in whole cell lysates obtained from control or RhoA-expressing cardiomyocytes. In the presence of cycloheximide, exogenously expressed full-length msPINK1 protein levels were time-dependently decreased in control cells and the time course of msPINK1 degradation was significantly slowed in response to RhoA expression (Fig. 5C). Thus, we surmise that RhoA activation appears to regulate mitochondrial PINK1 protein levels by inhibiting its degradation.

Under basal conditions PINK1 protein is partially imported into healthy mitochondria and embedded in the mitochondrial membrane. The transmembrane segment of PINK1 is cleaved by an inner-mitochondrial membrane protease and the cleaved PINK1 is then retro-translocated to the cytosol where it undergoes proteasomal degradation [18, 61, 62]. To determine whether RhoA regulates the cleavage of PINK1, cells co-expressing msPINK1 with GFP (Ctrl) or RhoA were treated with MG132, a proteasome inhibitor, and whole cell lysates were isolated for WB analysis to measure accumulation of cleaved msPINK1. In control cells treated with MG-132, cleaved msPINK1 accumulated in a time-dependent manner; this response was significantly inhibited in cells expressing RhoA (Fig. 5D). Importantly, endogenous PINK1 cleavage assessed in the presence of MG-132 was also attenuated by RhoA expression (Fig. 5E). These results suggest that RhoA prevents PINK1 cleavage and its subsequent proteasomal degradation.

### Protein kinase D (PKD) activation is required but not sufficient for RhoA-mediated PINK1 accumulation at mitochondria

**Fig. 6:**
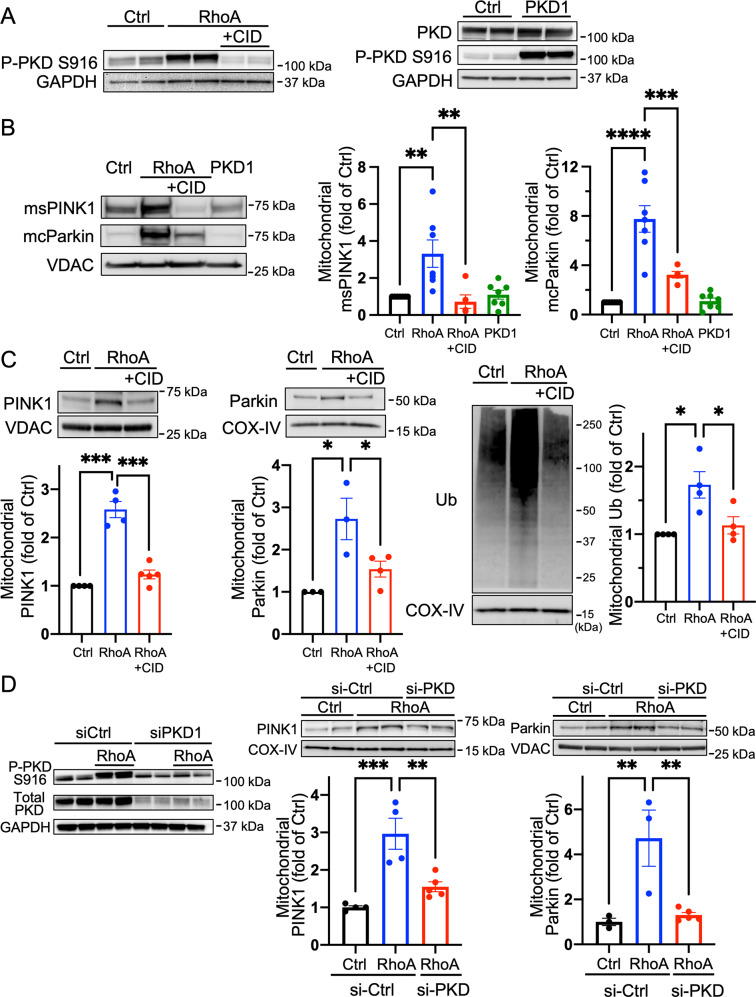
PKD is required but not sufficient for RhoA-mediated PINK1 and Parkin accumulation at mitochondria. **A** NRVMs were adenovirally infected with GFP (Ctrl), RhoA or wild-type PKD1, and PKD activation was assessed by P-S916 PKD (autophosphorylation site) WB in whole cell lysates. A pharmacological inhibitor of PKD, CID755673 was used at 50 μM. GAPDH was used as the loading control. **B** GFP (Ctrl), RhoA or PKD were co-expressed with msPINK1 and mcParkin in NRVMs for 16 h in the absence or presence of CID755673 as above. Mitochondrial fractions were isolated and subjected to WB. VDAC was used as a mitochondrial marker and loading control. *n* = 5–7, **p < 0.01, ***p < 0.001, ****p < 0.0001. **C** GFP (Ctrl) or RhoA was adenovirally expressed in NRVMs and PKD activity was inhibited by CID755673. Endogenous PINK1, Parkin and ubiquitinated proteins in mitochondrial fractions were examined by WB. VDAC and COX-IV was used as mitochondrial markers as well as loading controls. *n* = 3–5, *p < 0.05, ***p < 0.001. **D** PKD expression was suppressed in NRVMs using siRNA to PKD1, then GFP (Ctrl) or RhoA was expressed, and whole cell lysates or mitochondrial fractions were isolated for WB. GAPDH was use as loading control in whole cell lysates, while COX-IV and VDAC were used as loading controls in mitochondrial fractions. *n* = 3-4,**p < 0.01, ***p < 0.001.

Our previous studies have shown that RhoA-mediated cardioprotection is effected in part through protein kinase D1 (PKD1) [35, 45]. We confirmed that RhoA expression activates PKD by examining its autophosphorylation at S916, and this was abolished by treatment with a pharmacological inhibitor of PKD, CID755673 (Fig. 6A). When cells were treated with CID755673, RhoA-mediated msPINK1 and mcParkin accumulations in the mitochondrial fractions were significantly attenuated (Fig. 6B). RhoA-induced increases in endogenous PINK1, Parkin, and ubiquitinated proteins in the mitochondrial fractions were likewise inhibited by CID755673 (Fig. 6C).

We further confirmed the requirement of PKD through siRNA-mediated knockdown of PKD1 demonstrating that siRNA-mediated knockdown of PKD1 attenuated RhoA-mediated PINK1 and Parkin accumulations in the mitochondrial fractions (Fig. 6D). Surprisingly, while adenoviral overexpression of wild-type PKD1 was associated with an increase in its activation state (Fig. 6A), it did not induce msPINK1 and mcParkin increases at mitochondria (Fig. 6B), suggesting that PKD activation is required but not sufficient for RhoA-mediated PINK1 accumulation at mitochondria.

### Localization of active RhoA at mitochondria is PKD-dependent

**Fig. 7:**
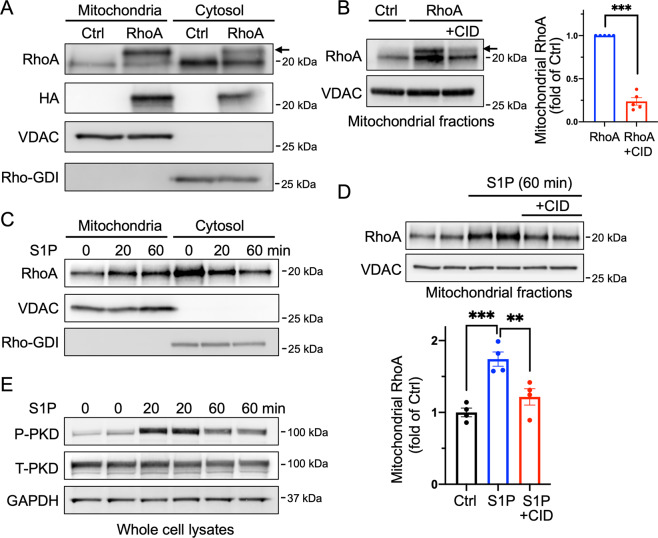
Active RhoA localizes at mitochondria through PKD activation. **A** Mitochondrial and cytosolic fractions were isolated from NRVMs expressing GFP (Ctrl) or RhoA (HA-tagged) and subjected to WB. RhoA antibody detects endogenous wild-type RhoA as well as exogenous HA-L63RhoA (upper band indicated by an arrow). VDAC and Rho-GDI were used as mitochondrial and cytosolic markers respectively and served as gel loading controls. **B** NRVMs were adenovirally infected with GFP (Ctrl) or RhoA, and treated with CID755673 (50 μM), and mitochondrial fractions were subjected to WB for RhoA. Exogenously expressed RhoA (upper band indicated by an arrow) was quantified. *n* = 5, ***p < 0.001. **C** NRVMs were treated with 1 μM S1P for 20 and 60 min. Mitochondrial and cytosolic fractions were subjected to WB for endogenous RhoA. VDAC and Rho-GDI were used as mitochondrial and cytosolic markers respectively and served as loading controls. **D** NRVMs were stimulated with 1 μM S1P for 60 min in the absence or presence of CID755673 (50 μM). Mitochondrial fractions were isolated and subjected to WB. Endogenous RhoA levels in the mitochondrial fractions were quantified. VDAC was used as a mitochondrial marker and loading control. *n* = 4, **p < 0.01, ***p < 0.001. **E** NRVMs were treated with 1 μM S1P for 20 and 60 min and whole cell lysates were analyzed for P-S916 PKD, total PKD and GAPDH (loading control).

We proceeded to examine the localization of RhoA. A substantial amount of adenovirally expressed active RhoA localizes to mitochondria in NRVMs (Fig. 7A) but this mitochondrial localization was prevented by PKD inhibition using CID755673 (Fig. 7B). Similarly, stimulation of cardiomyocytes with S1P increased endogenous RhoA levels in the mitochondrial fractions and this response was sustained for at least 60 min (Fig. 7C) and largely attenuated in cells treated with CID755673 (Fig. 7D). This inhibitory effect can be attributed to PKD, since S1P treatment also led to activation of PKD as indicated by increases in P-PKD (Fig. 7E). These data suggest that PKD mediates the response to RhoA by regulating its mitochondrial localization.

### RhoA interacts with PINK1 at mitochondria

**Fig. 8:**
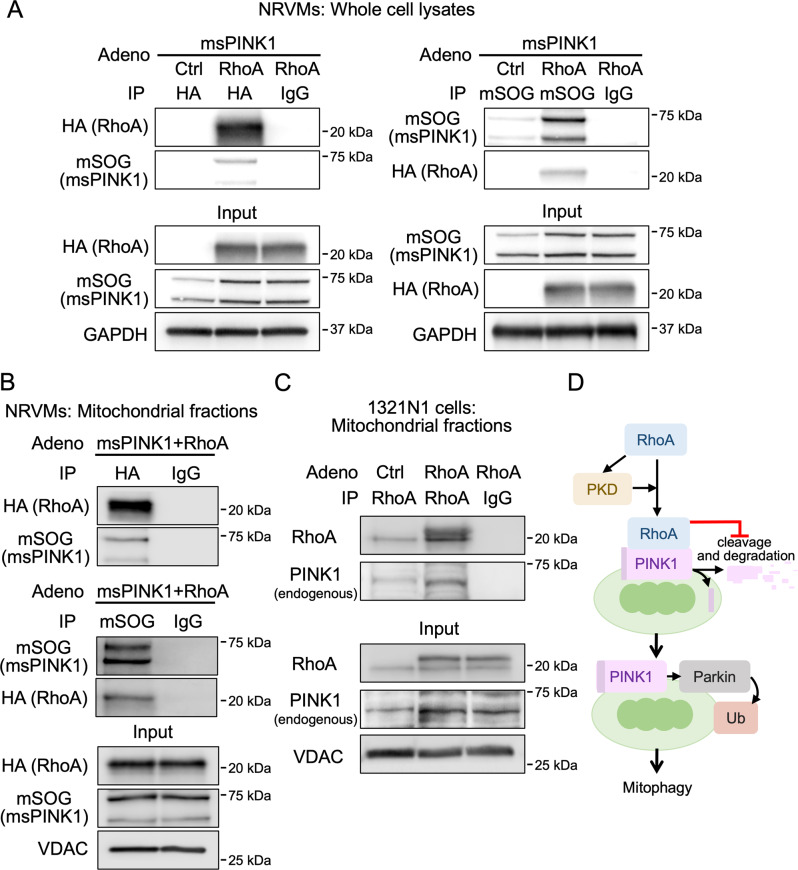
Activated RhoA interacts with PINK1 at mitochondria. **A** GFP (Ctrl) or HA-L63RhoA were co-expressed with msPINK1 in NRVMs and whole cell lysates were prepared. HA (RhoA, left) or miniSOG (msPINK1, right) were immunoprecipitated and subjected to WB for HA and miniSOG. Specific interaction between HA (RhoA) and miniSOG (PINK1) is deduced by co-precipitation of miniSOG with HA and the converse co-precipitation of HA with miniSOG. Duplicate gels demonstrating equal lysate usage in immunoprecipitations are shown, with GAPDH serving as the loading control. **B** Mitochondrial fractions were isolated from NRVMs expressing RhoA with msPINK1, and HA (RhoA) or miniSOG (msPINK1) were immunoprecipitated and subjected to WB for HA and miniSOG. VDAC served as a mitochondrial marker and the loading control. **C** 1321N1 astrocytoma cells were infected with GFP (Ctrl) or RhoA adenovirus, and mitochondrial fractions were isolated. RhoA was immunoprecipitated from the mitochondrial fractions and subjected to WB for RhoA and endogenous PINK1. VDAC served as a mitochondrial marker and the loading control. **D** Schematic illustration of RhoA-mediated PINK1 regulation and mitophagy.

Since RhoA appears to stabilize and limit degradation of PINK1, we sought to determine whether there was a physical interaction between RhoA and PINK1. RhoA was immunoprecipitated from whole cell lysates of NRVMs co-expressing msPINK1 with either GFP or HA-L63RhoA. msPINK1 (miniSOG) was detected in the RhoA (HA) immunocomplex (Fig. 8A, left). Conversely, when msPINK1 was immunoprecipitated using an miniSOG antibody, active RhoA (HA) was detected in the immunocomplex (Fig. 8A, right). This experiment was repeated using mitochondrial fractions (Fig. 8B), demonstrating that this interaction occurs at mitochondria. Endogenous PINK1 levels were too low to detect in the mitochondrial RhoA immunocomplex from primary cultures of cardiomyocytes examined in Fig. 8B. To circumvent this, we prepared a greater quantity of mitochondria from cultured 1321N1 astrocytoma cells. Active RhoA was detected in the mitochondrial fractions of 1321N1 cells and endogenous PINK1 was detected in the RhoA immunocomplex (Fig. 8C).

## Discussion

It is well accepted that mitochondrial membrane depolarization stabilizes PINK1 at mitochondria, leading to Parkin translocation to mitochondria, and consequent induction of mitophagy [17, 20, 21, 23, 24]. However, the intracellular signaling pathways that regulate this process have not been fully elucidated. We demonstrate here, through studies using either expression of active RhoA or activation of endogenous RhoA with the GPCR ligand S1P, that RhoA signaling leads to accumulation of PINK1 at mitochondria, triggering the translocation of Parkin to mitochondria. This in turn leads to ubiquitination of mitochondrial proteins, recognition of mitochondria by autophagosomes and their lysosomal degradation. Our in vitro and in vivo studies support previous findings that induction of mitophagy is protective against ischemic stress [15, 26–31, 48], and introduce the novel concept that this process can be regulated by RhoA signaling. Mitochondrial PINK1 accumulation induced by RhoA is not explained by increased PINK1 transcription or by mitochondrial membrane depolarization but is instead due to PINK1 protein stabilization through suppression of its cleavage. We suggest that the molecular interaction of RhoA with PINK1 at mitochondria prevents PINK1 cleavage, ensuing sequelae of events culminating in mitophagy (Fig. 8D). To our knowledge, this is the first demonstration that a G-protein signaling pathway can modulate mitophagy through a process distinct from the classical membrane depolarization-dependent pathway.

### RhoA-induced PINK1 accumulation at mitochondria

Our in vitro and in vivo studies using RhoA adenovirus, AAV9-RhoA and S1P demonstrate that activation of RhoA in cardiomyocytes induces PINK1 accumulation at mitochondria. We also demonstrate that PINK1 accumulates at mitochondria in response to activated RhoA in human astrocytoma and rat hepatocyte cell lines, indicating that RhoA regulation of PINK1 accumulation is not a cell-type or species-specific signaling event. Although previous studies have shown that RhoA signaling plays a pivotal role in regulating gene transcription [47, 58–60, 63], it does not affect PINK1 mRNA levels. Mitochondrial membrane depolarization is also unaffected by RhoA activation, indicating that RhoA-mediated increases in mitochondrial PINK1 is independent of the canonical mitochondrial membrane potential depolarization pathway. Experiments examining PINK1 turnover in the presence of cycloheximide demonstrate that PINK1 protein stability is increased in cells expressing active RhoA. This is accompanied by decreased formation of cleaved PINK1, as evidenced by our findings that accumulation of cleaved msPINK1 or endogenous PINK1 in the presence of MG-132 is significantly inhibited by RhoA. These results suggest that the increase in full-length PINK1 at mitochondria results from inhibition of PINK1 cleavage. Through co-immunoprecipitation studies, we demonstrate that RhoA interacts with PINK1. Mechanistically, we suggest that formation of this molecular complex impedes the cleavage of PINK1. Interestingly, a recent study showed that binding of BNIP3 to PINK1 inhibits the cleavage of PINK1 [64]. Although the precise mechanism by which RhoA prevents PINK1 degradation remains to be elucidated, our results suggest that receptor agonists and interventions that activate RhoA represent a novel means of stabilizing PINK1 to stimulate mitophagy without concomitantly diminishing mitochondrial membrane potential and function.

### PKD and RhoA

Our previous studies showed that PKD is activated through RhoA signaling and that PKD1 inhibits Bax translocation to mitochondria, suppressing apoptosis [35, 45]. Through pharmacological inhibition and siRNA-mediated knockdown of PKD1, we demonstrate that PKD1 is required for PINK1 protein stabilization by RhoA. Additionally, experiments using PKD1 adenovirus in cardiomyocytes revealed that PKD1 activation alone does not induce PINK1 accumulation at mitochondria (Fig. 6B and Supplementary Fig. 1). Thus, PKD1 plays a regulatory role in RhoA-mediated PINK1 accumulation at mitochondria but is not a sufficient stimulus to increase PINK1 stability. Inhibition of ROCK, another established downstream effector of RhoA, using the pharmacological inhibitor Y-27632, also did not affect RhoA-induced mitochondrial accumulation of PINK1 (Supplementary Fig. 2).

Interestingly, we show that a significant portion of adenovirally expressed active RhoA localizes at mitochondria, and that S1P treatment likewise increases endogenous RhoA localization at mitochondria, concomitant with increase in PKD activation. Active RhoA localization at mitochondria is regulated through PKD activation since pharmacological inhibition of PKD significantly attenuates this. To our knowledge, this is the first study that demonstrates mitochondrial RhoA localization and its regulation through PKD. Our finding that PKD also has the ability to regulate mitophagy in concert with RhoA activation suggests an important role for PKD in autophagy and mitophagy, extending previous studies demonstrating that PKD stimulates autophagosome formation [65, 66].

### RhoA and cardioprotection

We previously reported that RhoA signaling stimulates survival pathways including Akt, FAK and PKD, providing protection against oxidative stress in cardiomyocytes [35, 43, 45, 47]. RhoA-induced FAK activation leads to Akt stimulation [43], which inhibits opening of the mitochondrial permeability transition pore [52, 67]. Activation of PKD inhibits the translocation of active Bax, a pro-apoptotic protein, to mitochondria [45]. These findings demonstrate that RhoA signals through several pathways that block mitochondrial death pathways under oxidative stress conditions. The current study reveals an additional novel mechanism for RhoA-mediated protection against ischemia involving PINK1 and mitophagy. The contribution of this critical mitochondrial quality control mechanism to RhoA-mediated cardioprotection is supported by the demonstration that the ischemic protection provided by RhoA is attenuated by siRNA knockdown of PINK1 in cardiomyocytes. Parkin has also been reported to exert anti-apoptotic effects by increasing the threshold for the release of cytochrome c and inhibiting the apoptotic function of Bax [68, 69] and this could also contribute to the protection mediated by RhoA. Although it is still controversial [70], mitochondrial fission is another mechanism that was originally suggested to facilitate mitophagic degradation by segregating the damaged part of the mitochondrion [71, 72]. Our recent work demonstrated that RhoA signaling facilitates mitochondrial fission through regulation of Drp1 phosphorylation, contributing to RhoA-mediated protection [42]. Thus, mitochondria are a convergence point for RhoA-mediated cellular protection, with RhoA signaling through multiple pathways to preserve the integrity of mitochondria by inhibition of mitochondrial death pathways and enhancing removal of compromised mitochondria during stress.

It has been widely accepted that autophagy and mitophagy, activated by energy depletion and ischemic stress, serve as protective mechanisms [26, 27, 48], although some controversy remains. In addition to PINK1/Parkin-mediated mitophagy, another form of mitophagy regulated by mitophagic receptors (FUNDC1, BNIP3, NIX) has been reported to be protective in some settings [73–76]. These molecules, PINK1/Parkin and mitophagic receptors, are essential for tagging compromised mitochondria and triggering their removal. Targeting these molecules therapeutically might be challenging, however, since their mitophagic effects are only elicited in response to stressors such as mitochondrial membrane depolarization and hypoxia [21, 74]. Additionally, BNIP3 and NIX were originally identified as pro-apoptotic molecules [76–78], thus limiting their suitability as cardioprotective drug targets. Our data suggest that pathways involved in RhoA signaling could serve as druggable targets to facilitate mitophagy to prevent ischemic disease.

In summary, we have demonstrated that RhoA-mediated signals can regulate PINK1 protein stability and thereby stimulate protective mitophagy. We suggest that RhoA activates PKD, which in turn enhances RhoA localization at mitochondria where it interacts with and inhibits degradation of PINK1 resulting in full-length PINK1 accumulation, subsequent Parkin recruitment and mitochondrial protein ubiquitination, and culminating in the transport via autophagosomes to lysosomes for degradation. Mitophagy has attracted increasing attention as a target for the development of novel therapeutics and this study suggests that activation of RhoA may provide a pharmacological means of regulating mitophagy without compromising mitochondrial functions to promote cell survival.

## Materials and methods

All procedures were performed in accordance with the NIH Guide for the Care and Use of Laboratory Animals and approved by the Institutional Animal Care and Use Committee of UCSD.

### Cell Culture

Neonatal rat ventricular myocytes (NRVMs) were isolated, cultured and infected with adenovirus as described previously [48]. Briefly, myocytes were isolated from 1-2 day old Sprague-Dawley rats using a Neonatal Cardiomyocyte Isolation kit (Worthington Biochemical Corporation), plated on gelatin-coated dishes, and maintained overnight in Dulbecco’s modified Eagle’s medium (DMEM) supplemented with 15 % fetal bovine serum (FBS). Beginning the following day, cells were starved with serum-free DMEM for 24 h. 1321N1 cells (MilliporeSigma) and clone 9 cells (ATCC) were cultured in DMEM supplemented with 10% FBS and serum starved for 24 h.

Adenovirus for the HA-tagged active RhoA (HA-L63RhoA), msPINK1, mcParkin, PKD1, Mito-Keima and GFP (used as control) were used as described in our previous studies [48, 51, 52]. Adenovirus encoding mcParkin was provided by Dr. Asa Gustafsson (University of California, San Diego, La Jolla, CA, USA) and adenovirus encoding Mito-Keima was provided by Dr. Junichi Sadoshima (Rutgers New Jersey Medical School, Newark, New Jersey, USA). To knock down PINK1 or PKD1 expression, cells were transfected with pre-designed siRNA (Qiagen, Hilden, Germany; 2 μg/1 × 10^6^cells) for 48 h using DharmaFECT 1 transfection reagent [48, 51, 52].

### Adeno-associated virus serotype 9 (AAV9) and myocardial infarction

AAV9 injection and surgical induction of myocardial infarction (MI) were performed as described in our previous study [48]. Briefly, 8–12 wk old male and female mice (C57/BL6) were anesthetized with isoflurane (2%) and injected via tail vein with AAV9 in lactated Ringer’s solution (0.3 × 10^11^ viral particles/mouse). MI surgery was conducted on anesthetized mice by performing a left thoracotomy through the third intercostal space and ligating an 8–0 nylon suture around the left anterior descending (LAD) coronary artery. Age-matched sham-operated control mice underwent similar surgical procedures without ligation of the artery. Mice were randomly subjected to MI or sham surgery in each group.

### Reagents

The primary antibodies used were RhoA (#2117, for WB), Parkin (#2132), COX-IV (#11967), VDAC (#4661), lamin A/C (#2032), Rho-GDI (#2564), HA (#3724), PKD (#90039), P-PKD S916 (#2051), GAPDH (#2118), α-actinin (#3134) and LC3B (#3868) from Cell Signaling Technology; PINK1 from Novus Biologicals (#NB600-973); RhoA (SC-418, for IP) and ubiquitin (SC-8017) from Santa Cruz Biotechnology; miniSOG from Kerafast (#EFH004). Horseradish peroxidase (HRP)-conjugated secondary antibodies for WB, carbonyl cyanide 4-(trifluoromethoxy)phenylhydrazone (FCCP), cycloheximide (CHX), MG-132, CID755673, Bafilomycin A1, Evans blue and triphenyltetrazolium chloride (TTC) were purchased from MilliporeSigma. Y-27632 was purchased from Cell Signaling Technology. DharmaFECT-1 and LysoTracker Blue were purchased from Thermo Fisher Scientific. C3 exoenzyme was purchased from Cytoskeletton, Inc.

### Western blot

Lysates were prepared by homogenizing adult mouse ventricles or lysing cells in RIPA buffer (150 mM NaCl, 50 mM Tris (pH 7.4), 0.2 mM EDTA, 1% sodium deoxycholate, 1% NP-40 alternative, 0.1% SDS supplemented with 10 μg/ml aprotinin, 10 μg/ml leupeptin, 200  μmol Na_3_VO_4_, 1 mM PNPP and 1 mM PMSF) [48, 51, 52]. Samples were rocked at 4 °C for 10 min, spun down at 20,817 × *g* at 4 °C for 5 min and supernatants transferred to new tubes. After protein concentration measurement using micro BCA assay kit (Thermo Fisher Scientific), lysates were mixed with LDS sample buffer and reducing agent and heated at 75 °C for 15 min. Equal amounts of protein (15–60 μg) were loaded onto NuPAGE Bis-Tris gels (Thermo Fisher Scientific) and transferred to Polyvinylidene difluoride (PVDF) membrane (MilliporeSigma). Membranes were blocked with 5% milk in TBS-Tween at room temperature for 1 h and probed using primary antibodies diluted in 5% BSA/TBS-Tween at 4 °C overnight followed by incubation with 1:3000 dilution of HRP-conjugated secondary antibody and visualization using SuperSignal West Femto ECL detection reagents (Thermo Fisher Scientific). Full length original western blots for these results are provided in Supplementary material.

### Mitochondria and cytosolic fractionation

Mitochondria were isolated as previously described [48]. Briefly, cells were washed with cold PBS, harvested in mitochondrial isolation buffer (420 mM mannitol, 140 mM sucrose, 2 mM EDTA, 20 mM HEPES (pH 7.4), 0.025 % digitonin, 10 μg/ml aprotinin, 10 μg/ml leupeptin, 200 μM Na_3_VO_4_, 1 mM PMSF and 1 mM PNPP). Cell suspensions were passed through a 25-gauge needle five times and rocked at 4 °C for 20 min. Nuclei and unbroken cells were spun down by centrifugation (700 × *g* at 4 °C for 10 min). Supernatants were further cleared by centrifugation at 700 × *g* at 4 °C for 10 min. The resultant supernatants were spun at 16,000 × *g* at 4 °C for 15 min to pellet mitochondria while the supernatant was saved as the cytosolic fraction. The pellet was washed, resuspended in RIPA buffer and spun at 20,817 × *g* at 4 °C for 5 min. The supernatant was saved as the mitochondrial fraction. For isolation of mitochondria from adult mouse hearts, the ventricles were homogenized in mitochondrial isolation buffer and rocked at 4 °C for 15 min. The homogenates were centrifuged twice at 700 × *g* at 4 °C for 10 min to spin down nuclei and cell debris. Clarified supernatants were spun at 16,000 × *g* at 4 °C for 15 min. Mitochondrial pellets were resuspended in RIPA buffer, rocked at 4 °C for 10 min and centrifuged at 20,817 × *g* at 4 °C for 5 min and the supernatant was saved as the mitochondrial fraction.

### Immunoprecipitation

Cells were washed with cold PBS twice and lysed in 1 % digitonin buffer (20 mM Tris HCl (pH 7.2), 50 mM KCl, 1 mM MgCl_2_, plus protease and phosphatase inhibitors). After 30 min rocking at 4 °C, samples were spun down at 20,000 × *g* for 7 min and supernatants were saved. Supernatants were incubated with antibody conjugated with Dynabeads (Dynabeads Co-Immunoprecipitation kit from Thermo Fisher Scientific) at 4 °C overnight. Immunocomplexes were washed with cold lysis buffer three times, eluted using elution buffer, mixed with 2X LDS and DTT and boiled for 10 min for WB analysis.

### Cell death ELISA Assay

Cell death was measured using the Cell Death Detection ELISA^PLUS^ kit (Roche Applied Science) according to manufacturer’s instructions and as previously reported [48, 51, 52]. Briefly, NRVMs were lysed in cytosolic extraction buffer (125 mM NaCl, 20 mM β-glycerophosphate, 3 mM EDTA, 3 mM EGTA, and 0.3 % NP-40 alternative, 20 nM and Tris pH 7.6, plus protease and phosphatase inhibitors), and centrifuged at 20,000 × *g* at 4 °C for 5 min to pellet nuclei containing unfragmented DNA. Cytoplasmic supernatants were incubated with immunoreagents consisting of anti-histone-biotin antibody and anti-DNA-peroxidase antibody in streptavidin-coated wells of the provided microplate in the dark at room temperature for 2 h. Unbound antibodies were washed and a substrate solution containing 2,2’-azino-di-[3-ethylbenzthiazoline sulfonate-6] (100 μl per well) was added. Absorbance was measured at 405 nm using an Infinite M200 PRO plate reader (Tecan).

### Confocal imaging

Mito-Keima imaging was performed as previously described [48]. Briefly, cells were loaded with LysoTracker Blue at 250 nM for 2 h and washed twice with a modified Krebs-Henseleit buffer (121 mM NaCl, 5 mM NaHCO_3_, 4.7 mM KCl, 1.2 mM KH_2_PO_4_, 1.2 mM MgSO_4_, 1.8 mM CaCl_2_, 5 mM glucose and 10 mM Na-HEPES pH 7.4). Mito-Keima fluorescence was visualized at 37 °C using a Nikon confocal microscope. Mito-Keima signal was excited by 458 nm laser and by 561 nm, and Mito-Keima emission was detected between 610 and 640 nm. LysoTracker Blue was excited by 405 nm laser and fluorescence was detected between 415 nm and 450 nm. To measure mitochondria membrane potential, NRVMs were loaded with 50 nM of tetramethylrhodamine ethyl ester (TMRE) for 45 min in the presence of cyclosporine H (Tocris Bioscience) at 1.6 μM [79, 80] at room temperature and excited at 561 nm and detected between 570 nm and 620 nm. Fluorescence intensity was measured using ImageJ2 software.

### RhoA pull-down assay

The amount of active RhoA (GTP-RhoA) was determined using the Rho Activation Assay Biochem kit (Cytoskeleton, Inc.) as described previously [46, 63]. Briefly, hearts were homogenized in lysis buffer (50 mM Tris-HCl pH 7.2, 500 mM NaCl, 10 mM MgCl_2_, 0.1% SDS, 1% NP-40 alternative and supplemented with protease and phosphatase inhibitors). Protein concentration of the tissue lysates were measured and equivalent protein amounts were incubated with 50 µg Rhotekin-Rho Binding Domain beads on a rocker at 4 °C for 1 h. After washing to remove unbound proteins, LDS and DTT were added and the samples were boiled for 10 min to release activated GTP-bound RhoA from the beads and analyzed by WB.

### Statistical analysis

Results are reported as average ± SEM. Comparisons between two groups were accomplished using unpaired two-tailed Student’s test. Statistical significance was determined using ANOVA followed by the Tukey post hoc test. A *p* value of *p* < 0.05 was considered to be statistically significant.

## Supplementary information

Supplemental figure 1

Supplemental Figure 2

Raw western blot images

Author Contribution Form

Reproducibility Checklist

Related Manuscript File

## Data Availability

The data used to support the findings of this study are available from the corresponding author upon request.
