# Supplementary material for: RhoA signaling increases mitophagy and protects cardiomyocytes against ischemia by stabilizing PINK1 protein and recruiting Parkin to mitochondria
Source: Cell Death Differ. 2022 Jun 27;29(12):2472–86. doi: 10.1038/s41418-022-01032-w (PMC9751115; doi:10.1038/s41418-022-01032-w)
Supplement: Supplementary file 1 — Supplemental figure 1 [file 41418_2022_1032_MOESM1_ESM.pdf]

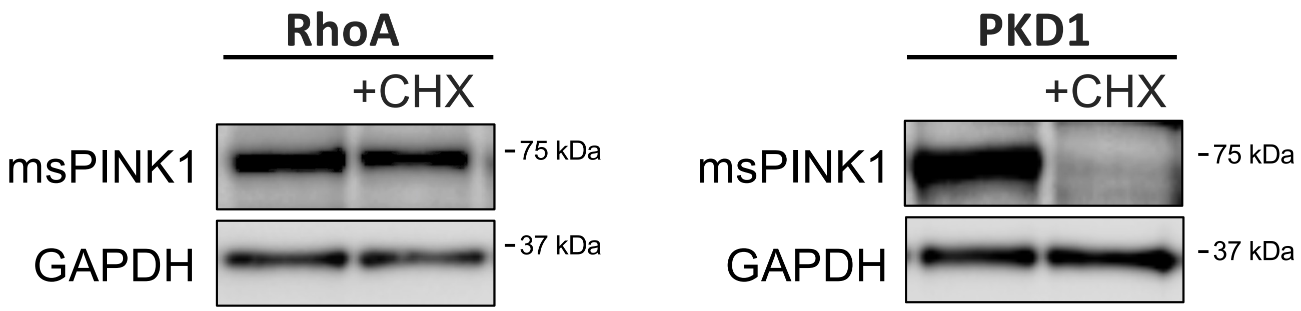

Supplementary Figure 1. PKD1 overexpression does not inhibit msPINK1 degradation. RhoA or PKD-1 were co-expressed with msPINK1 for 16 hrs, and then treated with cycloheximide (100 µg/ml) for 30 min. Whole cell lysates were subjected to WB for PINK1 (msPINK1) and GAPDH (loading control).
