# Supplementary material for: RhoA signaling increases mitophagy and protects cardiomyocytes against ischemia by stabilizing PINK1 protein and recruiting Parkin to mitochondria
Source: Cell Death Differ. 2022 Jun 27;29(12):2472–86. doi: 10.1038/s41418-022-01032-w (PMC9751115; doi:10.1038/s41418-022-01032-w)
Supplement: Supplementary file 2 — Supplemental Figure 2 [file 41418_2022_1032_MOESM2_ESM.pdf]

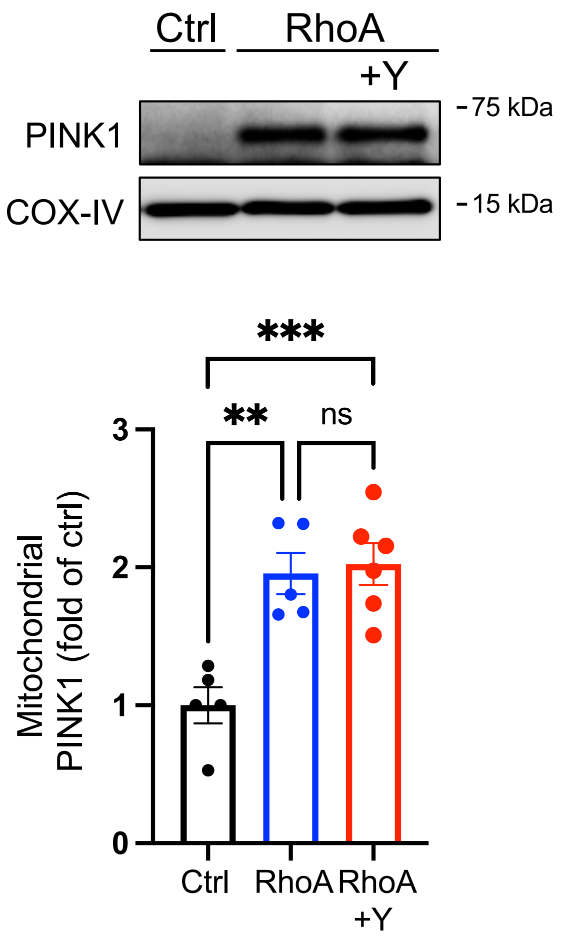

Supplementary Figure 2. Inhibition of ROCK does not affect RhoA-induced mitochondrial accumulation of PINK1.
GFP (Ctrl) or RhoA were adenovirally expressed in NRVMs and ROCK activity was inhibited by Y-27632 (10 μM). Mitochondrial fractions were isolated and subjected to WB for PINK1 and COX-IV (loading control). n=5-6; ** $p<0.01$, *** $p<0.001$.
