# Supplementary material for: RhoA signaling increases mitophagy and protects cardiomyocytes against ischemia by stabilizing PINK1 protein and recruiting Parkin to mitochondria
Source: Cell Death Differ. 2022 Jun 27;29(12):2472–86. doi: 10.1038/s41418-022-01032-w (PMC9751115; doi:10.1038/s41418-022-01032-w)

Fig. 1A, msPINK1

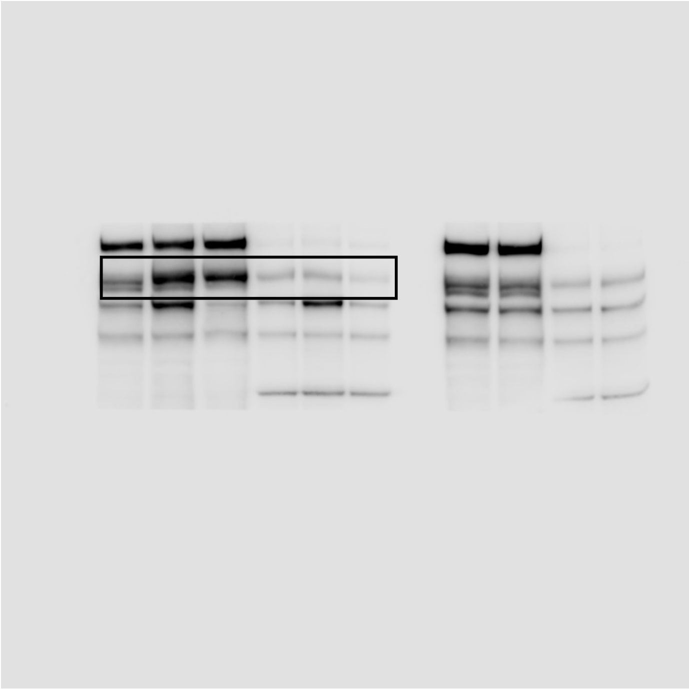

Fig. 1A, mcParkin

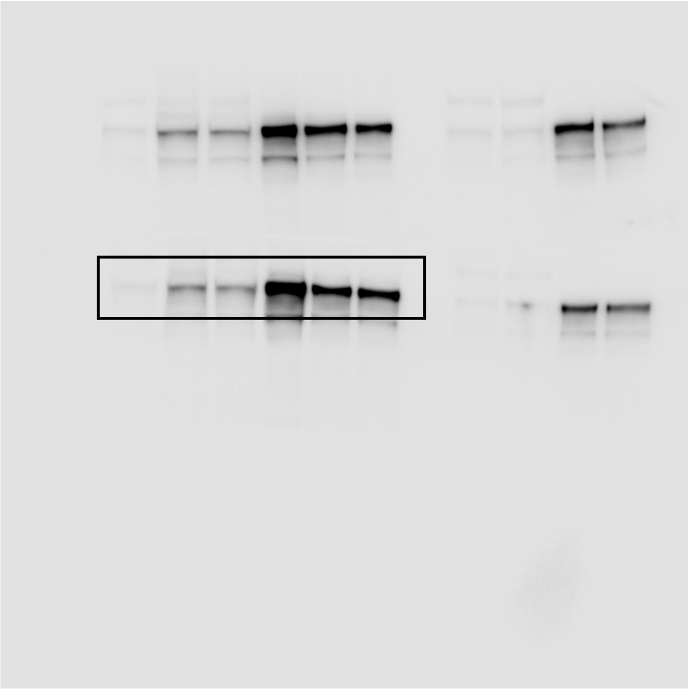

Fig. 1A, mcParkin long exposure

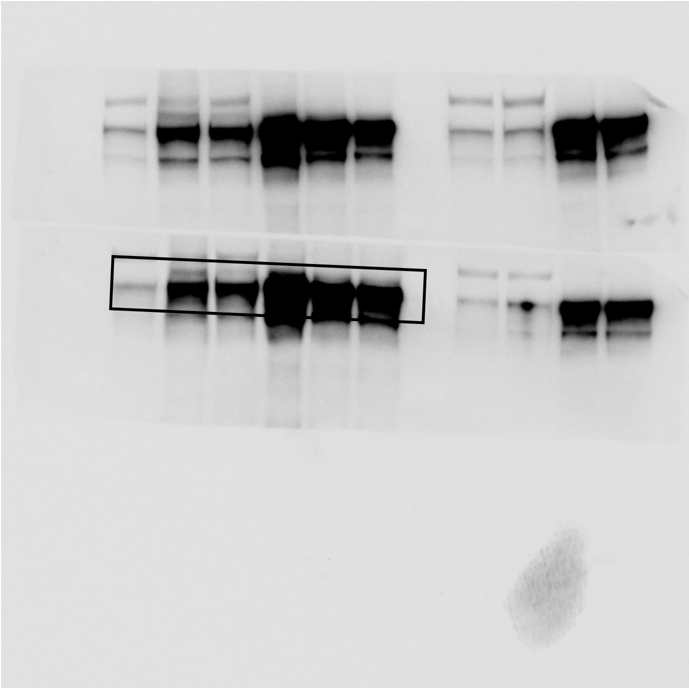

Fig. 1A, COX-IV

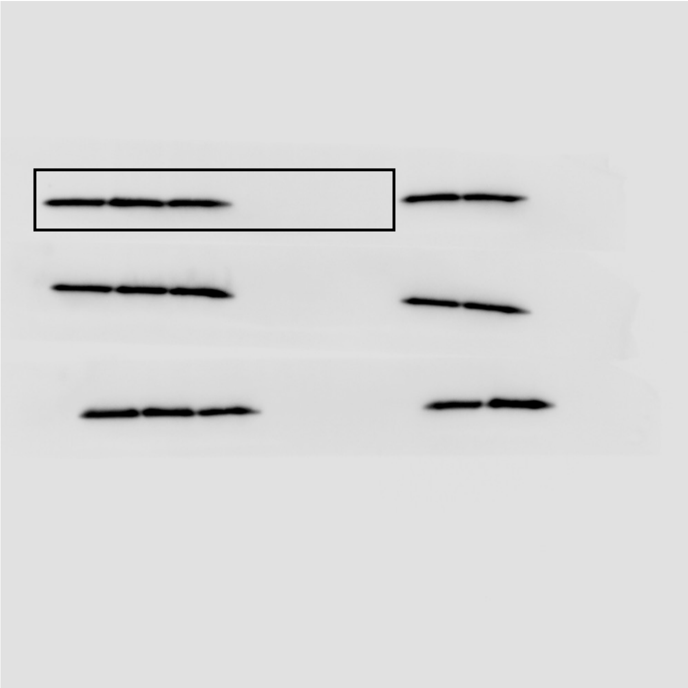

Fig. 1A, Rho-GDI

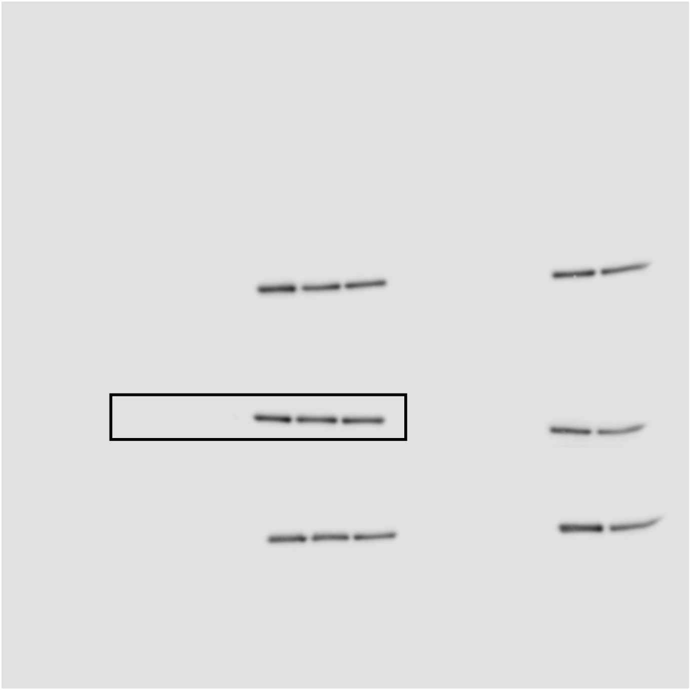

Fig. 1E, msPINK1

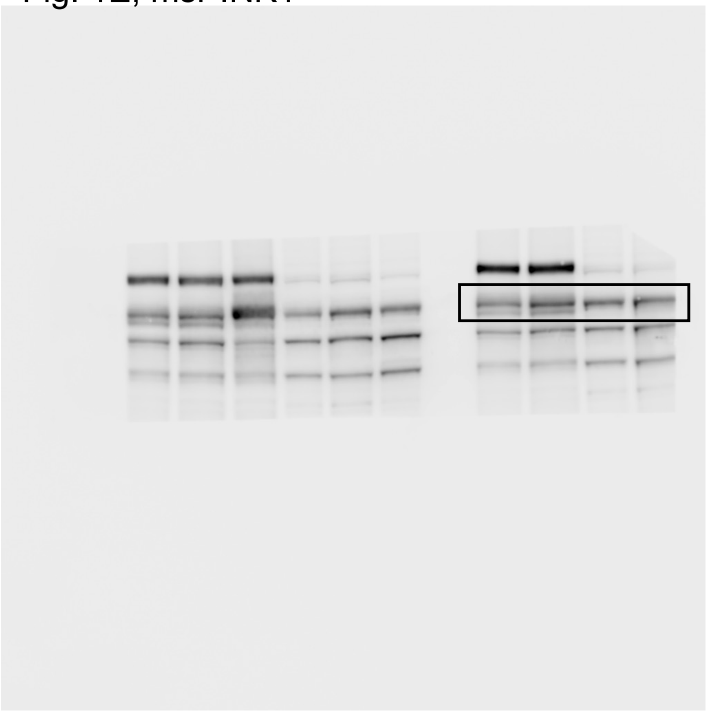

Fig. 1E, mcParkin

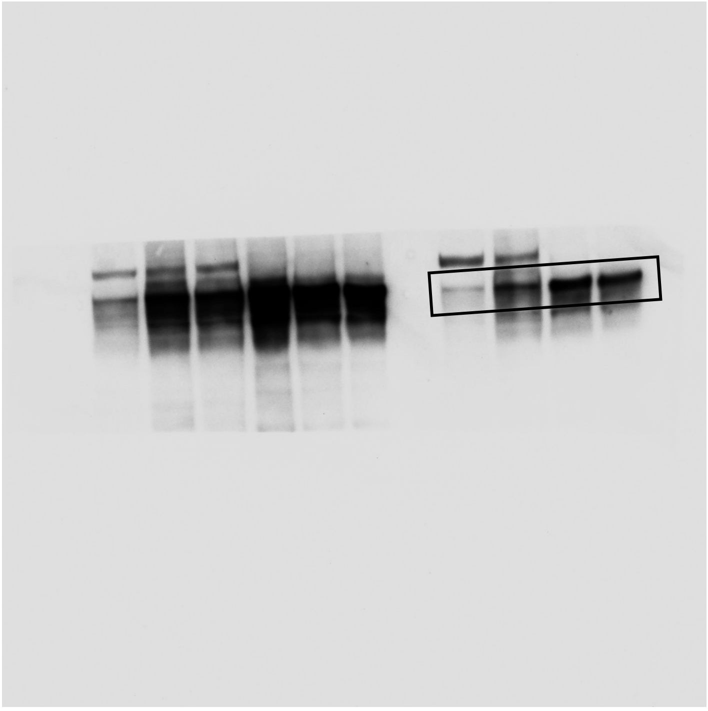

Fig. 1E, COX-IV

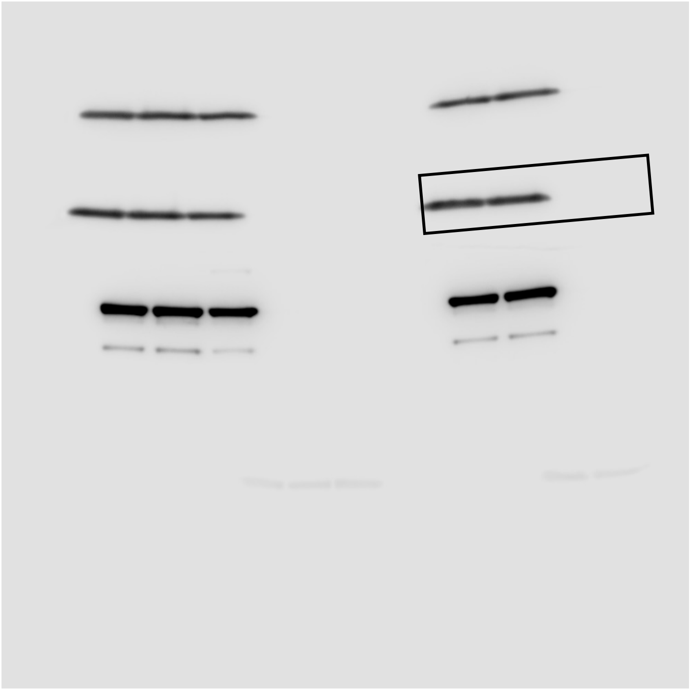

Fig. 1E, Rho-GDI

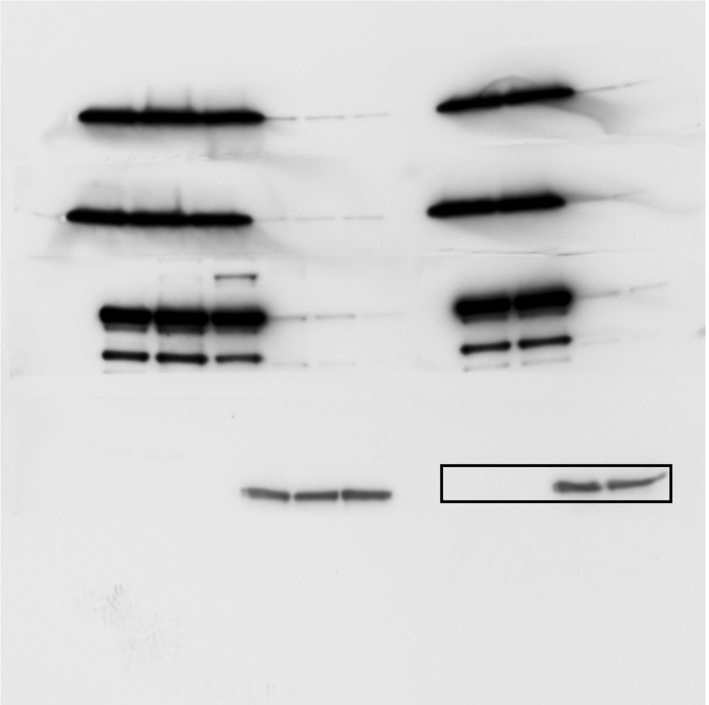

Fig. 2A, PINK1

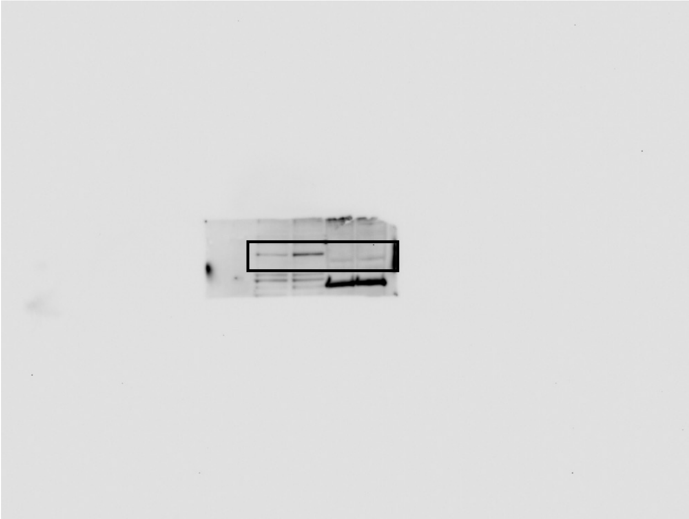

Fig. 2A, COX-IV

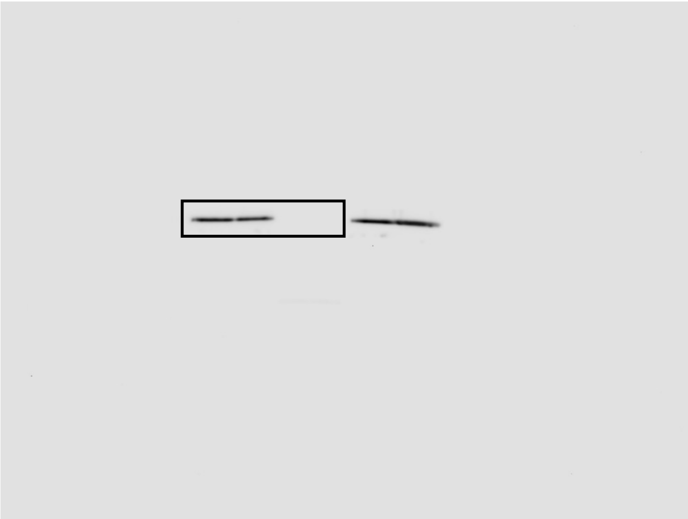

Fig. 2A, Rho-GDI

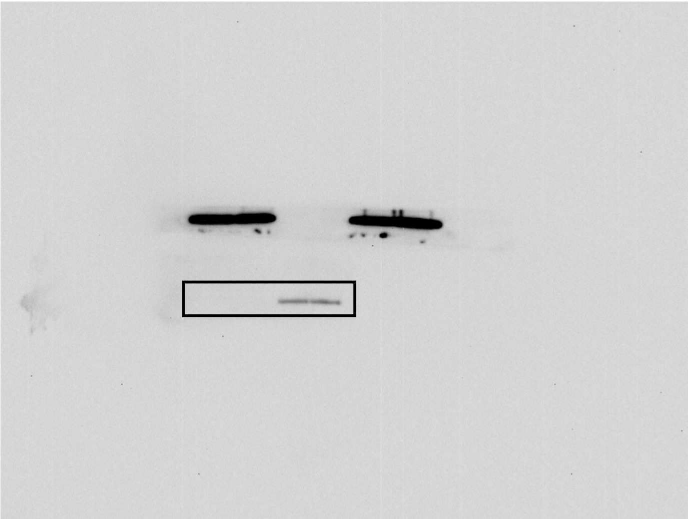

Fig. 2B, PINK1

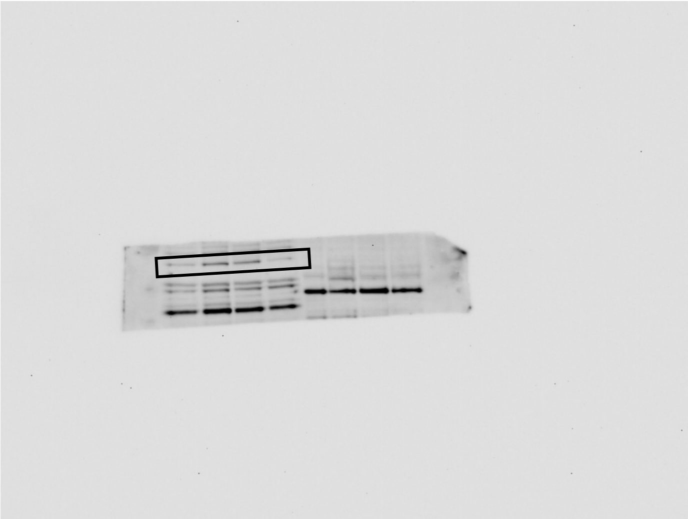

Fig. 2B, COX-IV

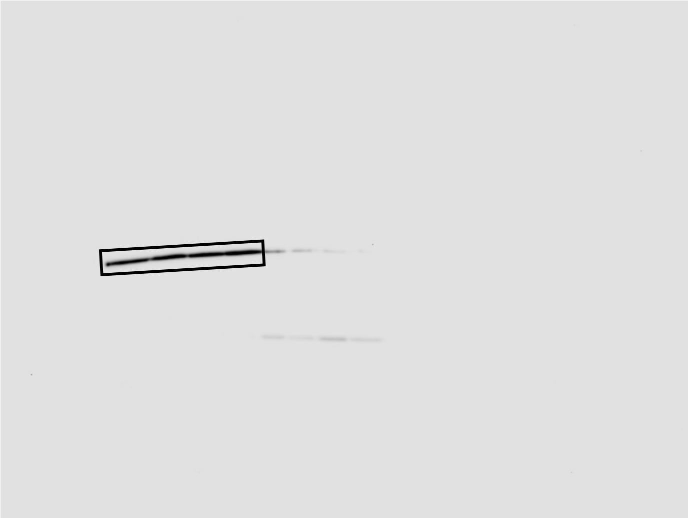

Fig. 2C, 1321N1 PINK1

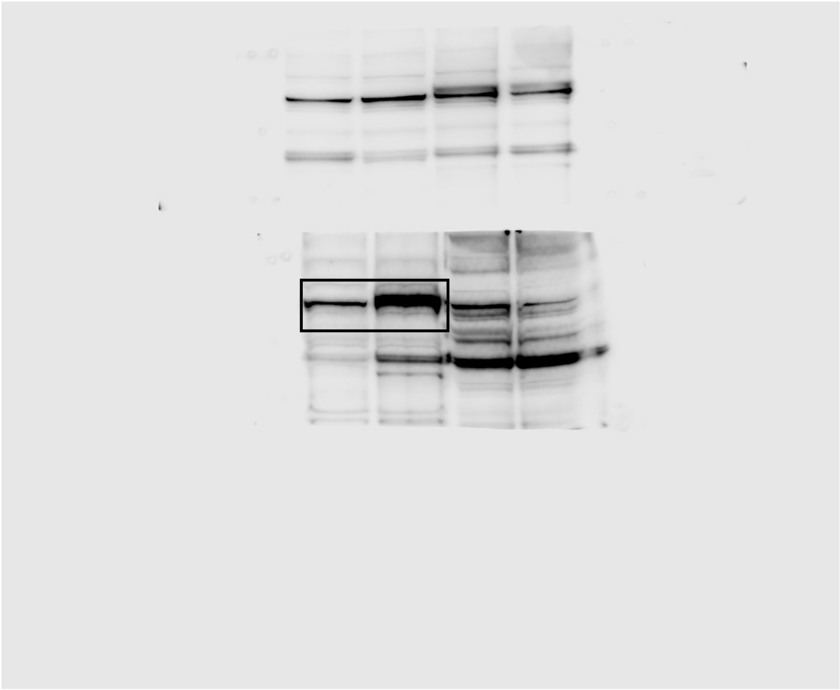

Fig. 2C, 1321N1 VDAC

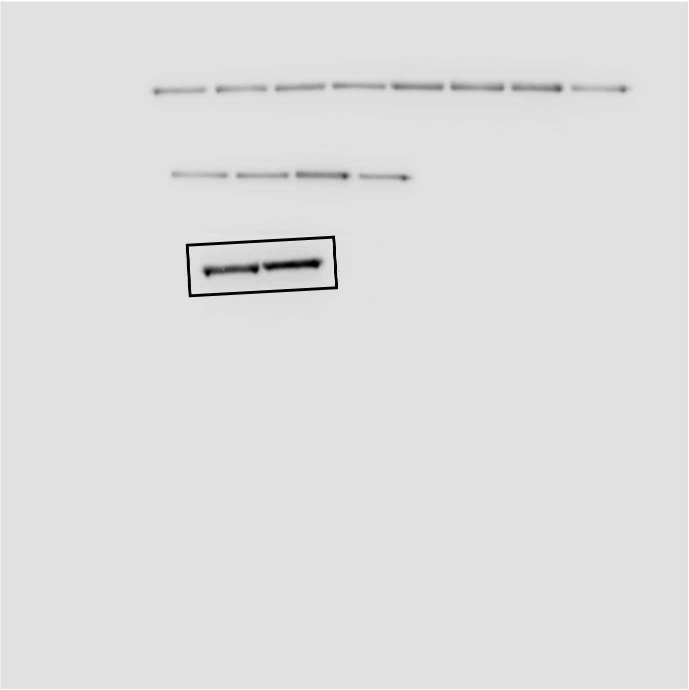

Fig. 2C, Clone 9 cells PINK1

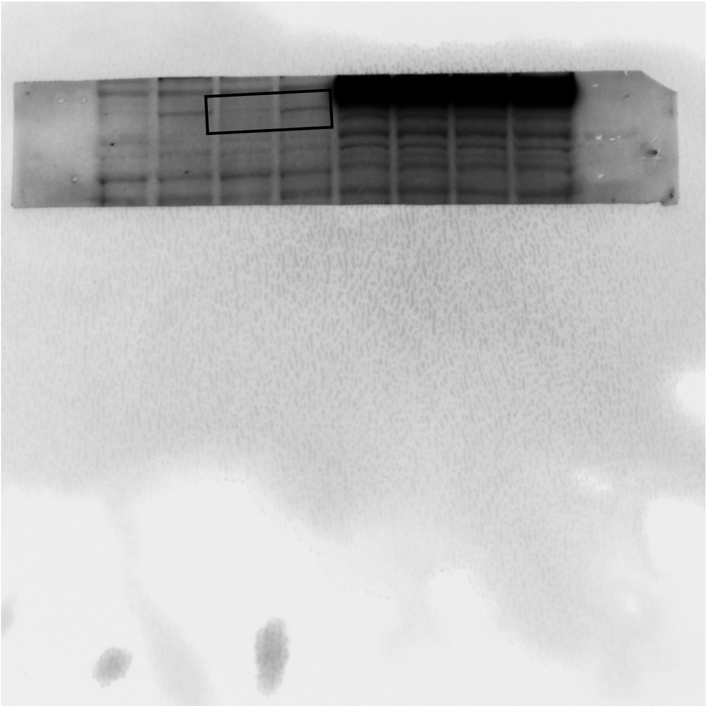

Fig. 2C, Clone 9 cells VDAC

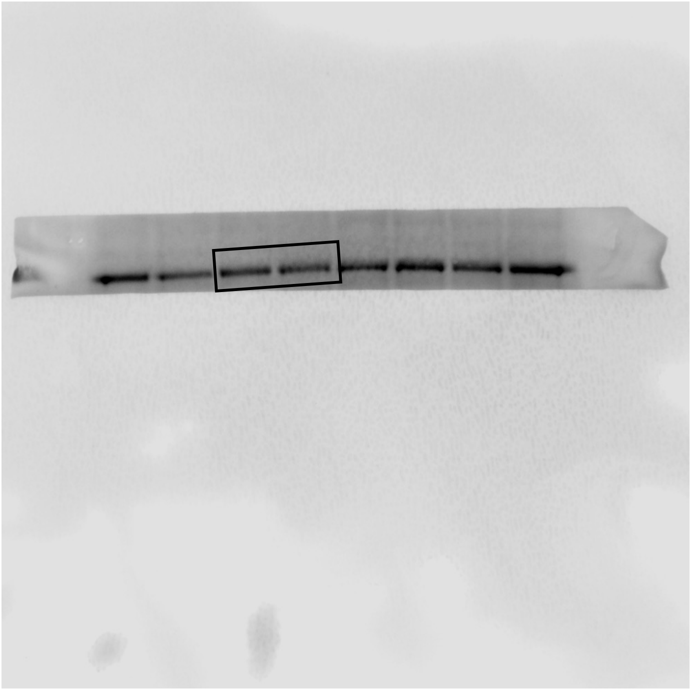

Fig. 3A, Parkin

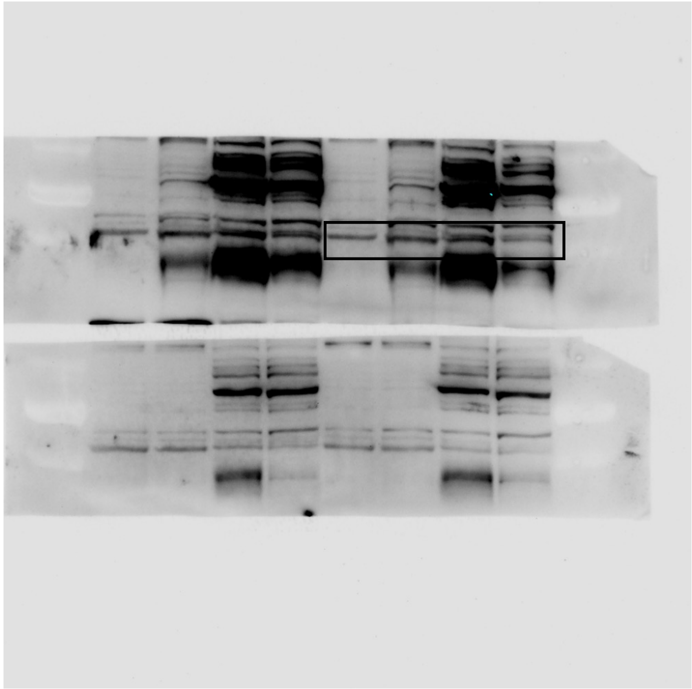

Fig. 3A, COX-IV (upper) and Rho-GDI (bottom)

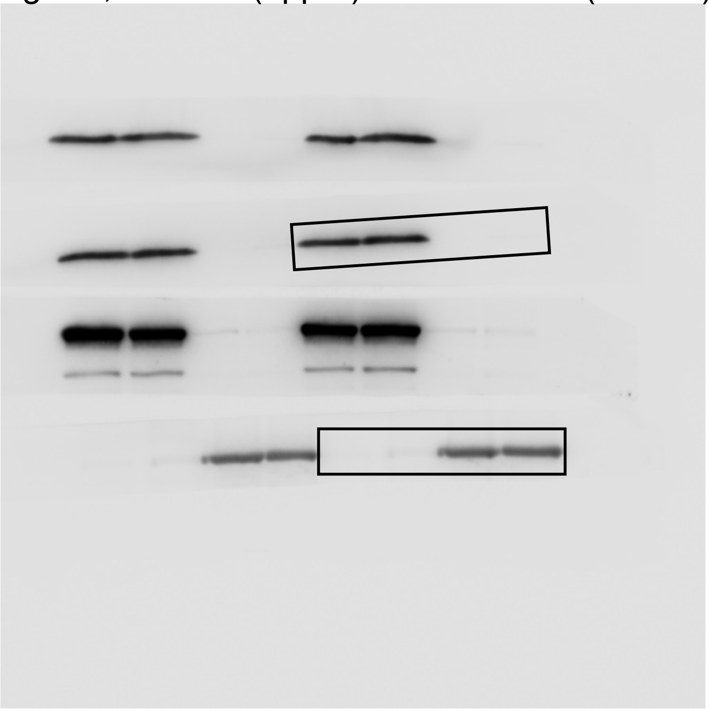

Fig. 3B, Ub

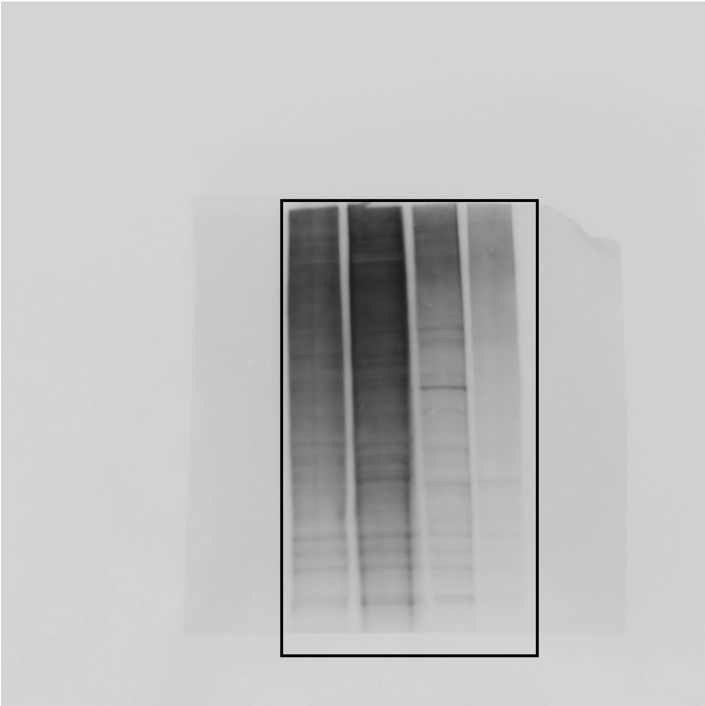

Fig. 3B, COX-IV (upper) and Rho-GDI (bottom)

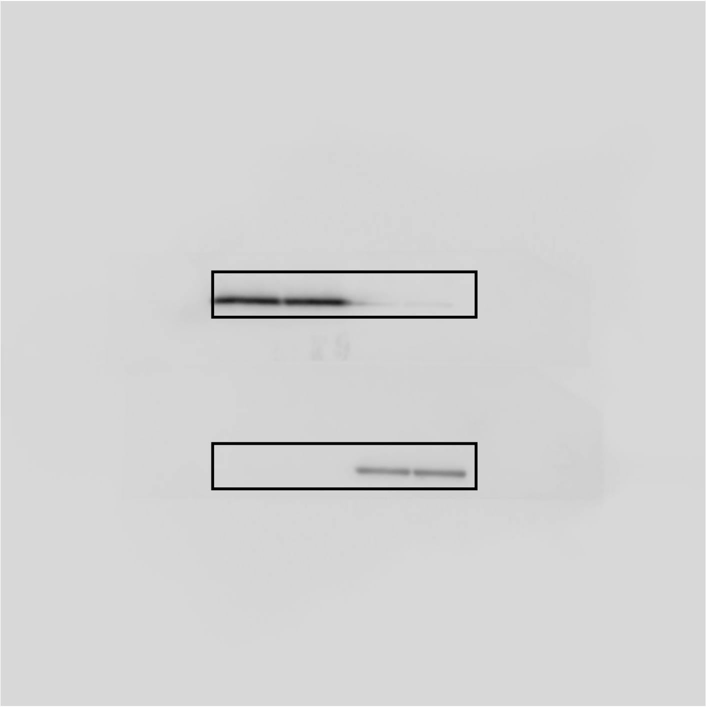

Fig. 3C, LC3-II

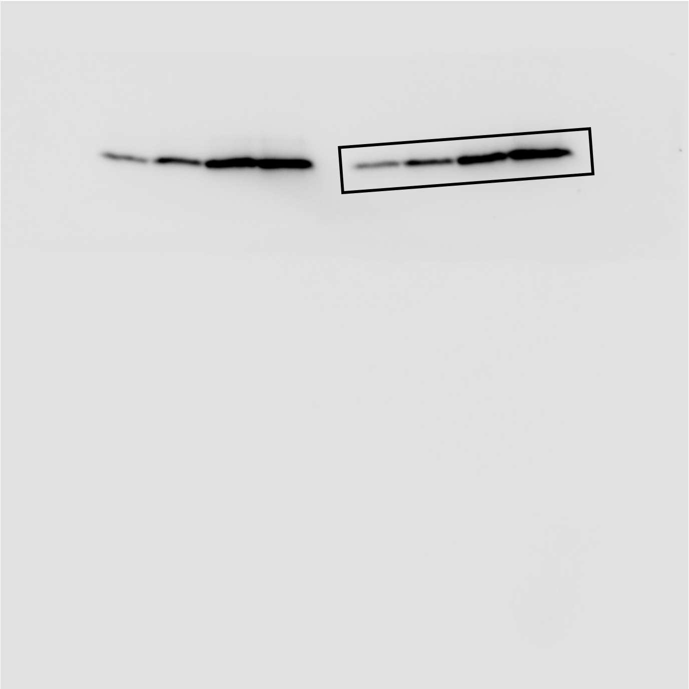

Fig. 3C, VDAC

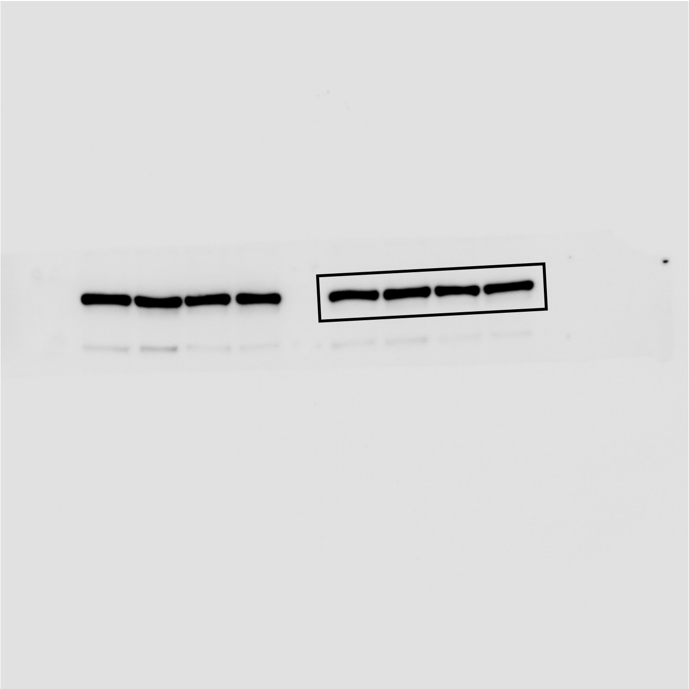

Fig. 3F, VDAC

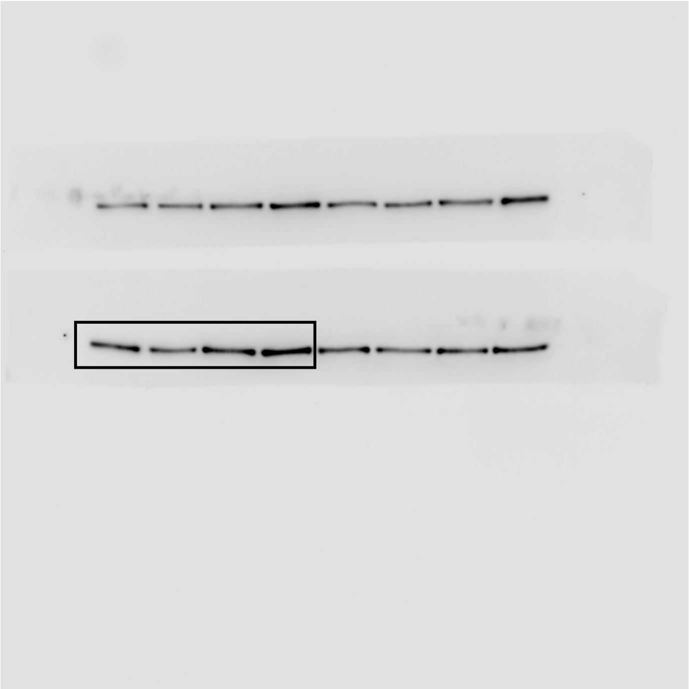

Fig. 3F, COX-IV

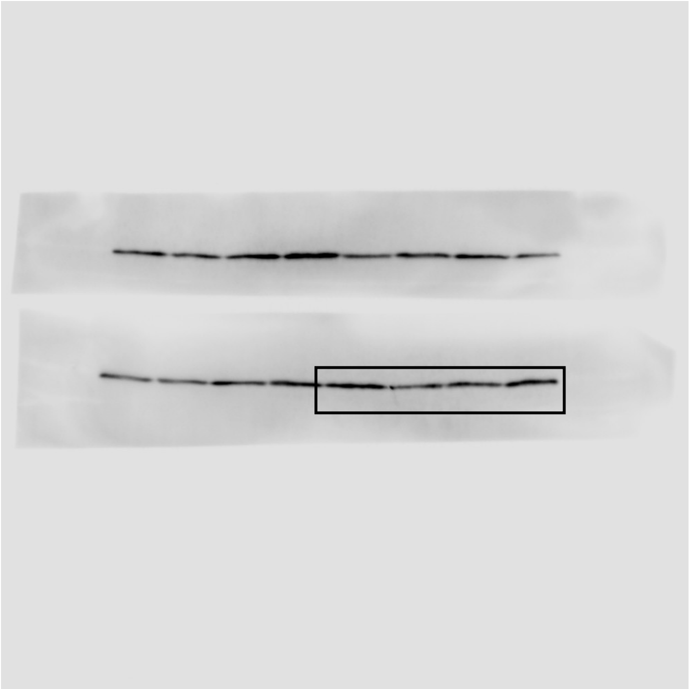

Fig. 3F, Lamin A/C

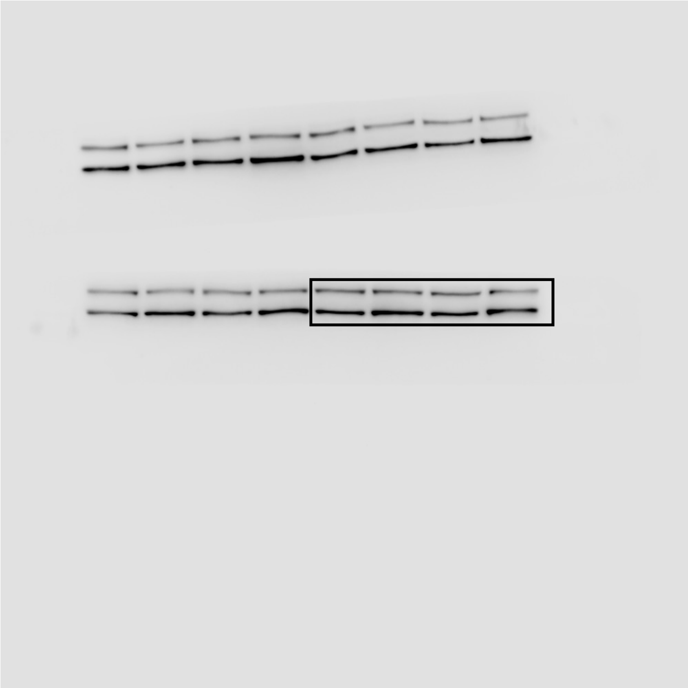

Fig. 3F, a-actinin

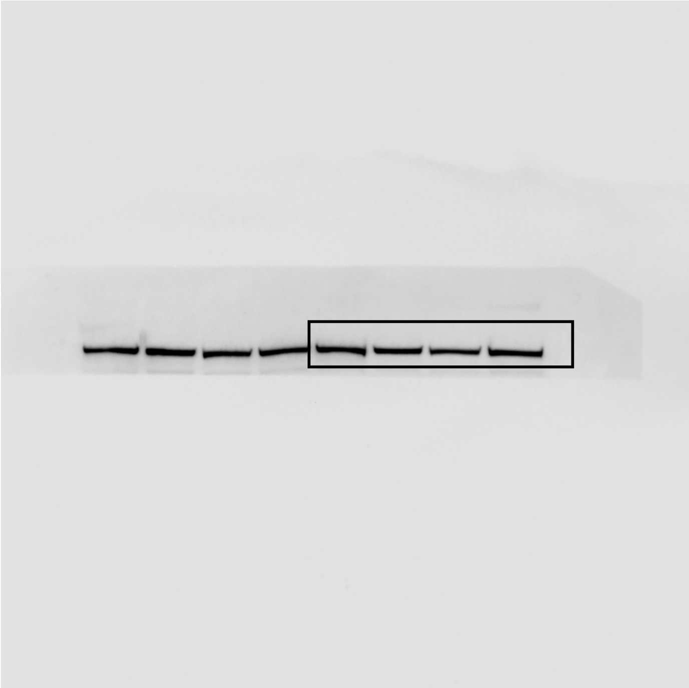

Fig. 4B, Total-RhoA

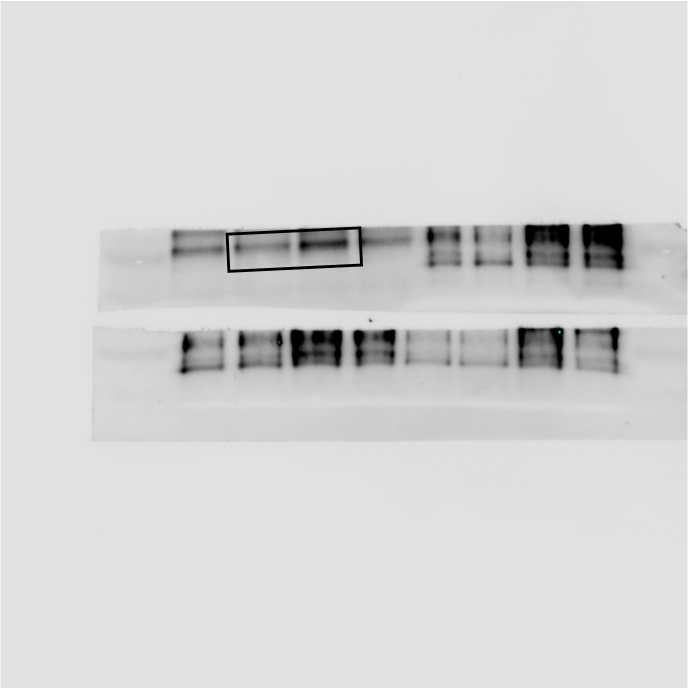

Fig. 4B, Active RhoA

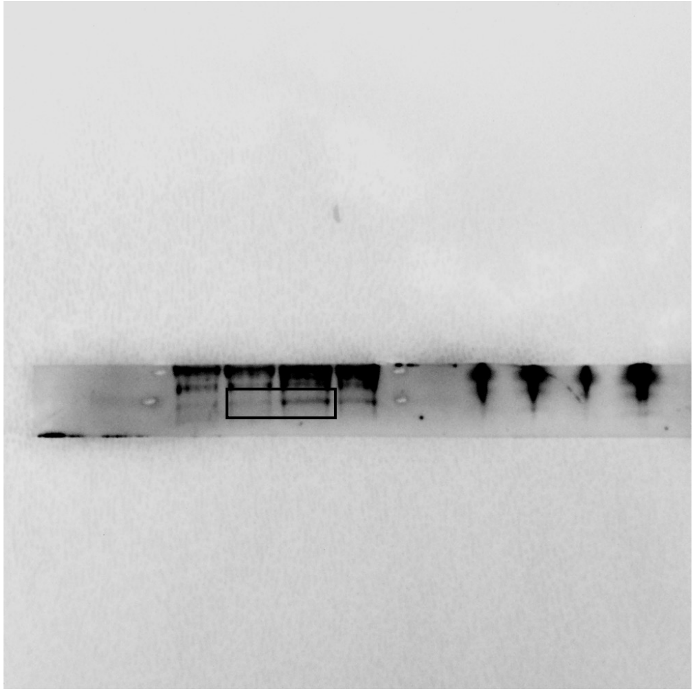

Fig. 4B, GAPDH

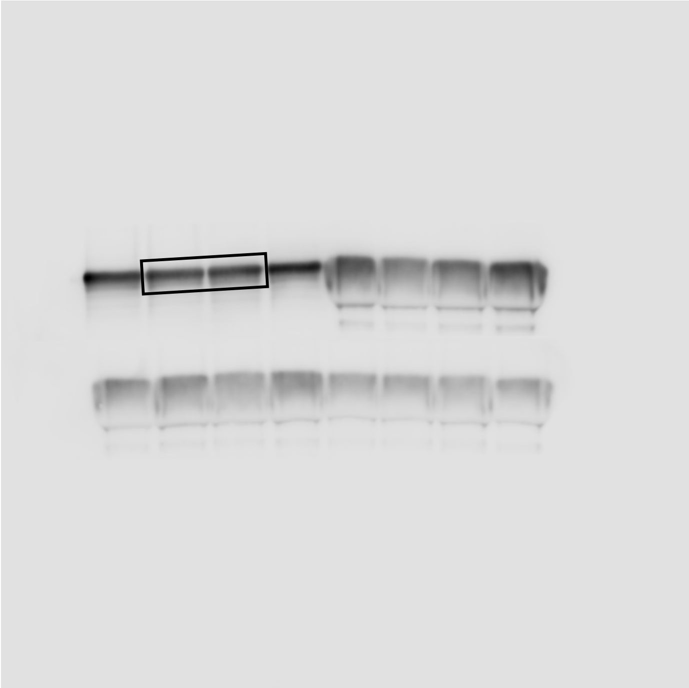

Fig. 4C, PINK1

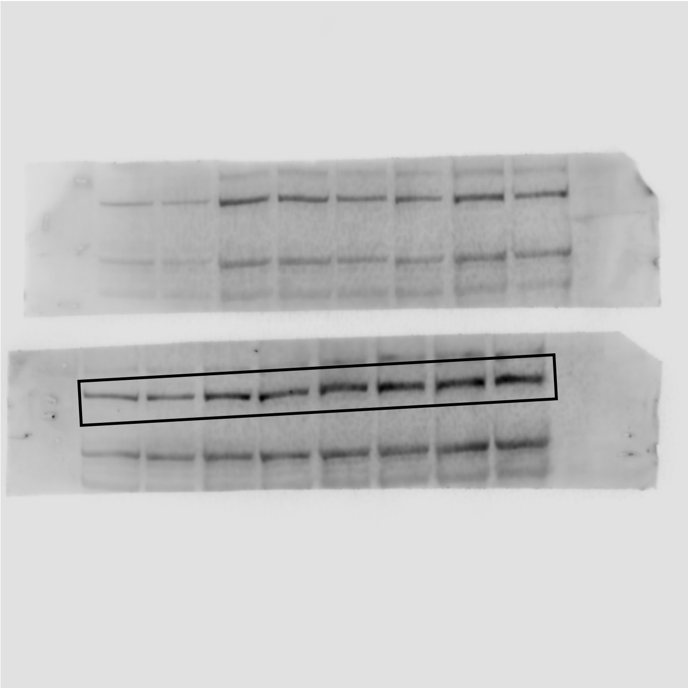

Fig. 4C, VDAC

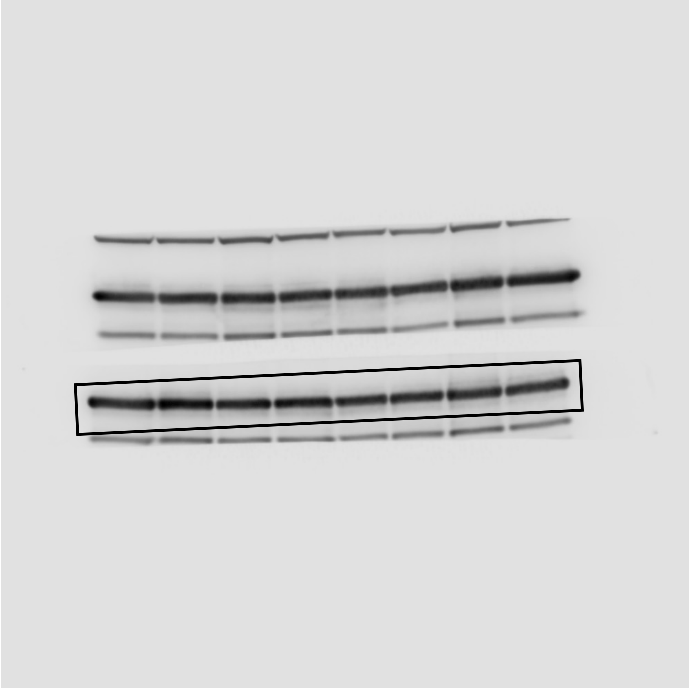

Fig. 4D, Parkin

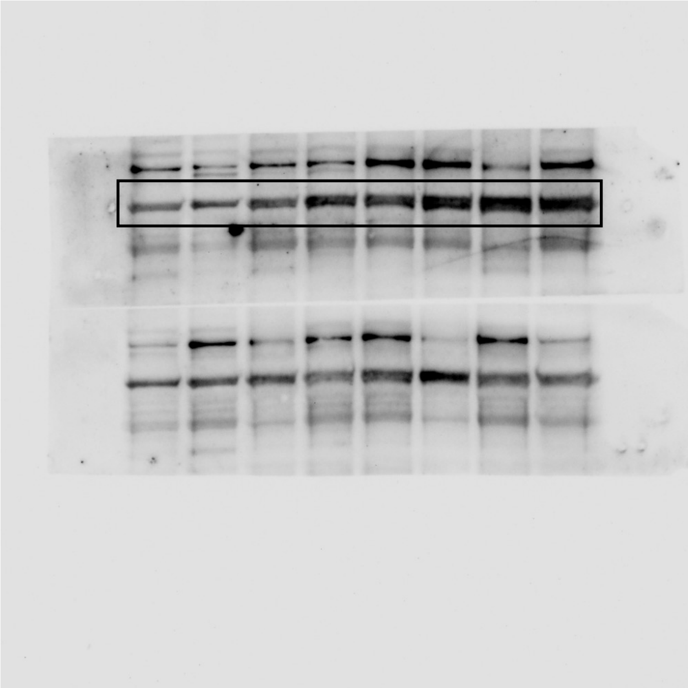

Fig. 4D, VDAC

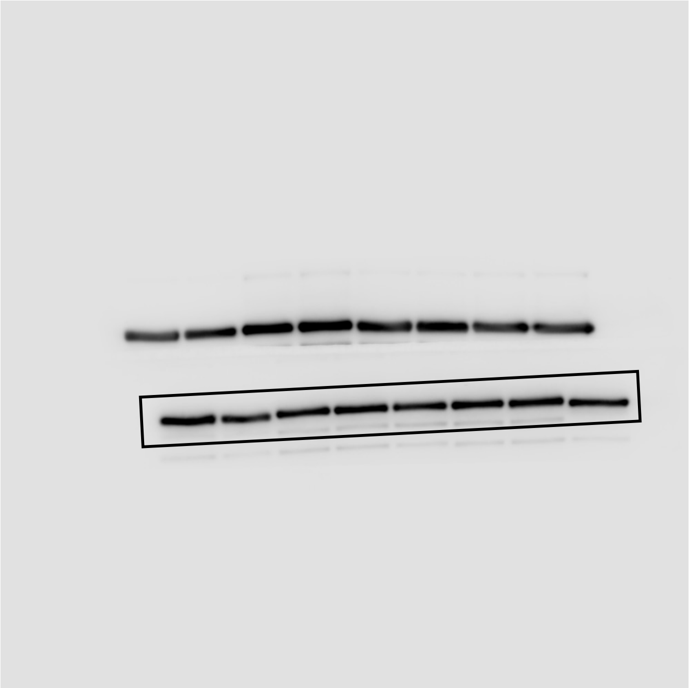

Fig. 4E, Ub

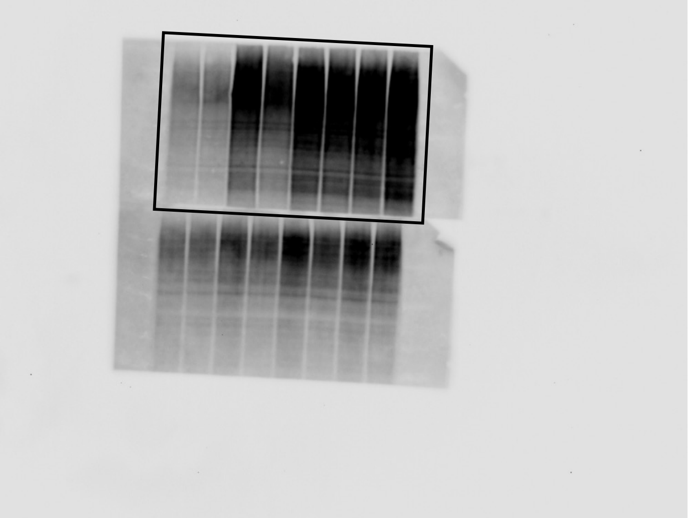

Fig. 4E, COX-IV

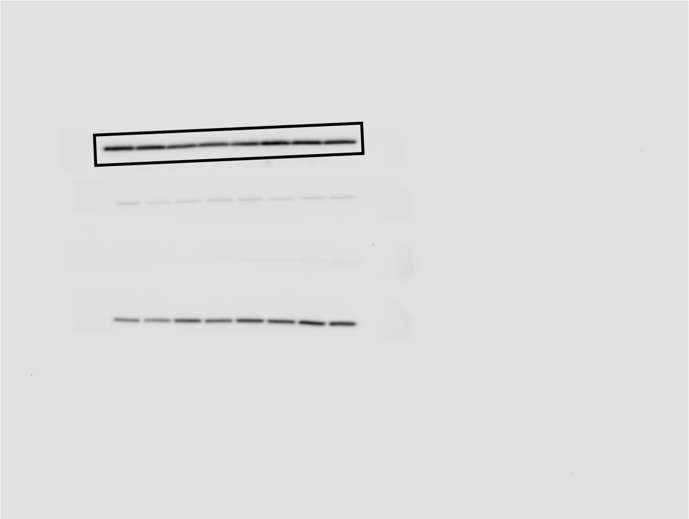

Fig. 5C, msPINK1 for Ctrl

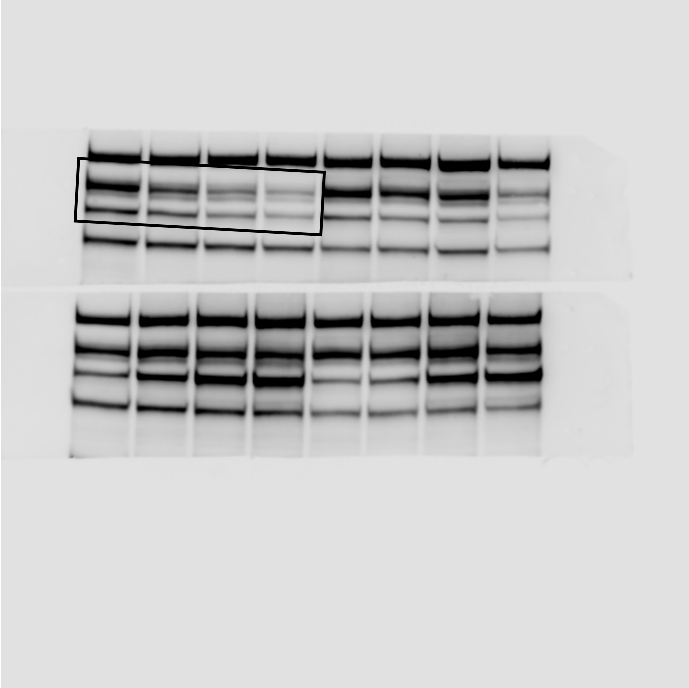

Fig. 5C, GAPDH for Ctrl

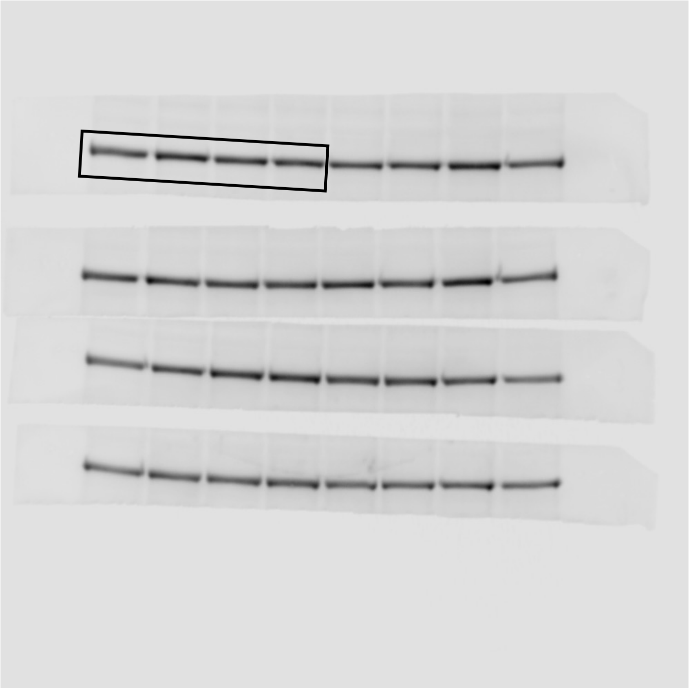

Fig. 5C, msPINK1 for RhoA

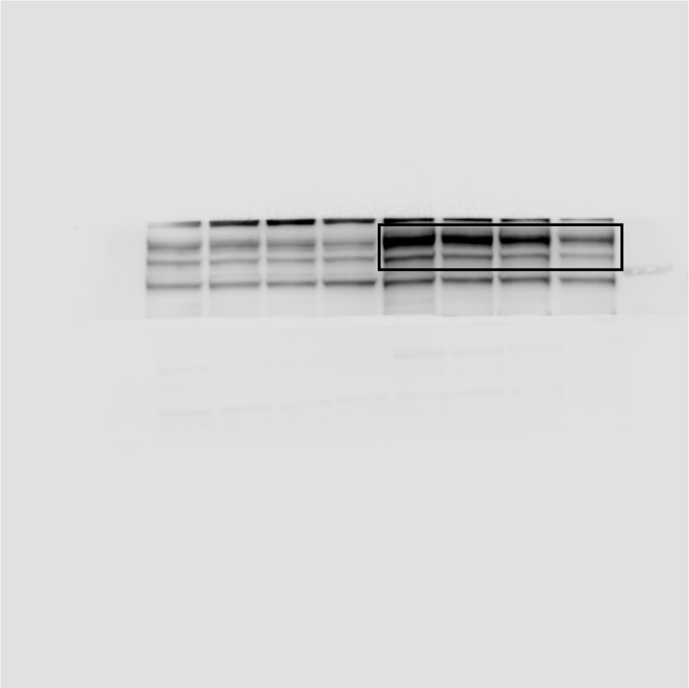

Fig. 5C, GAPDH for RhoA

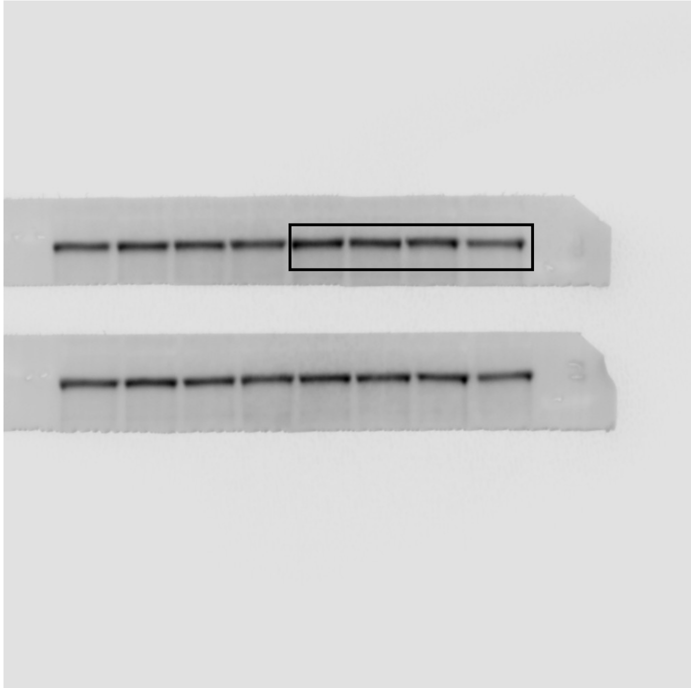

Fig. 5D, msPINK1 for Ctrl (left) and RhoA (right)

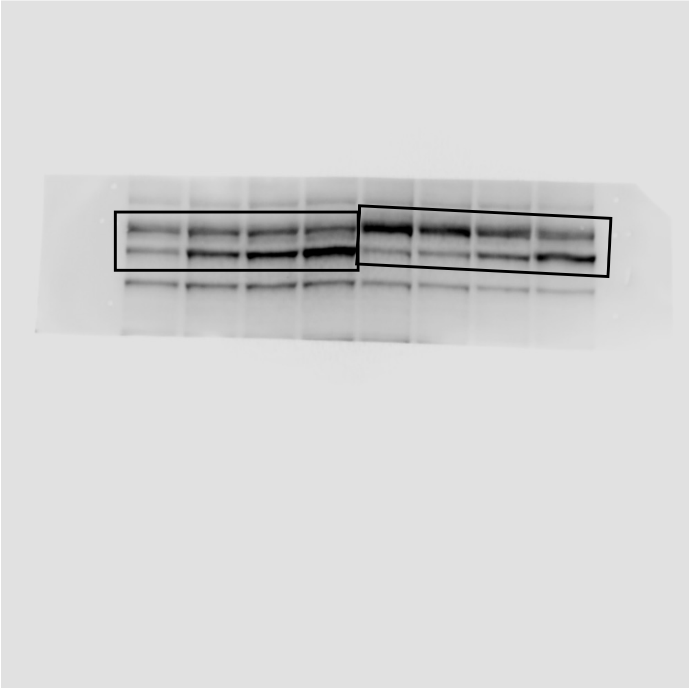

Fig. 5D, GAPDH for Ctrl (left) and RhoA (right)

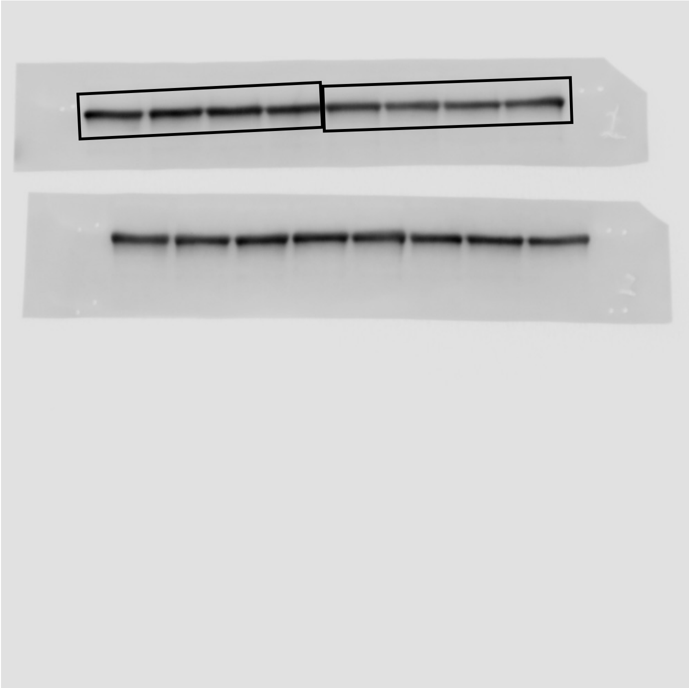

Fig. 5E, msPINK1 for Ctrl (left) and RhoA (right)

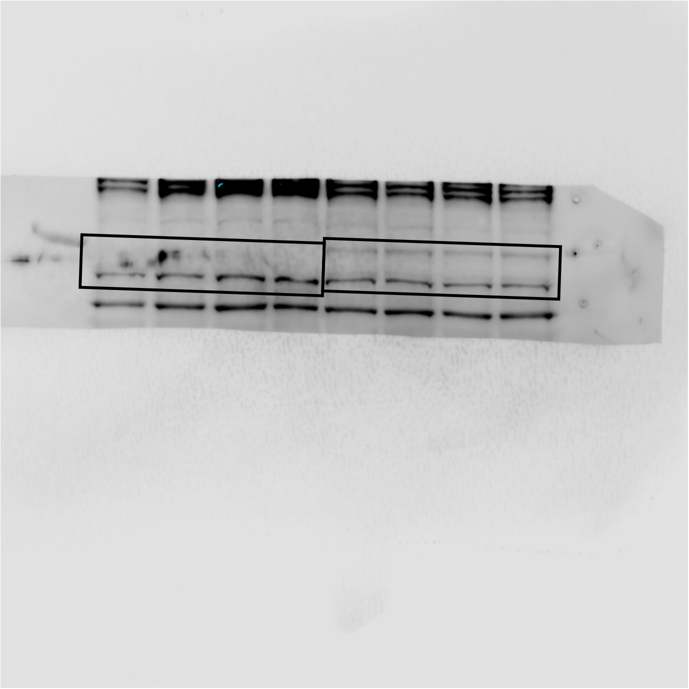

Fig. 5E, GAPDH for Ctrl (left) and RhoA (right)

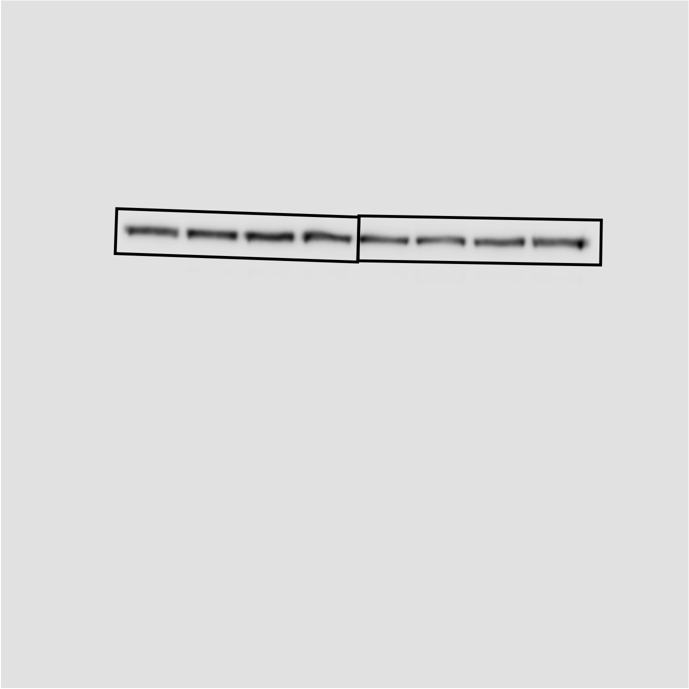

Fig. 6A, (left panel), P-PKD S916

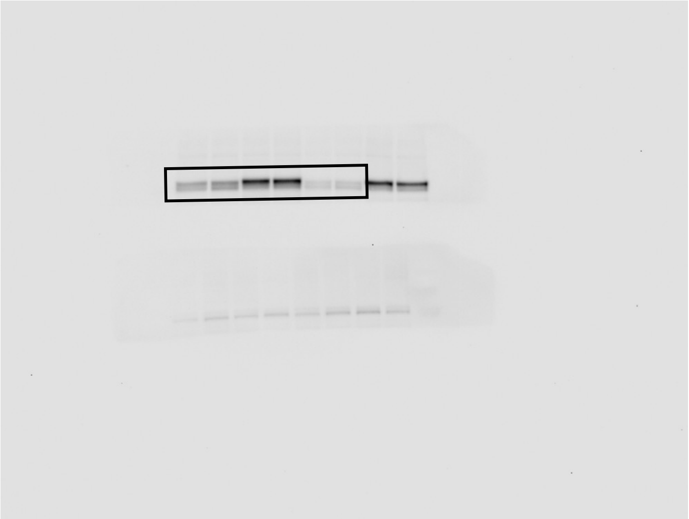

Fig. 6A, (left panel), GAPDH

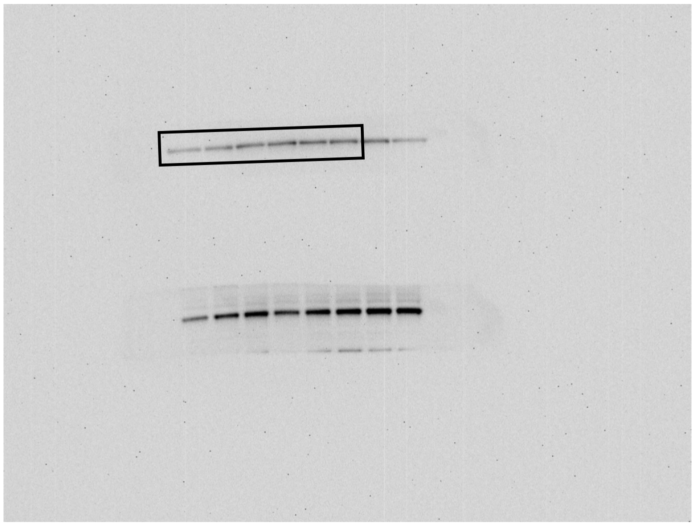

Fig. 6A (right panel), PKD (upper) and GAPDH (lower)

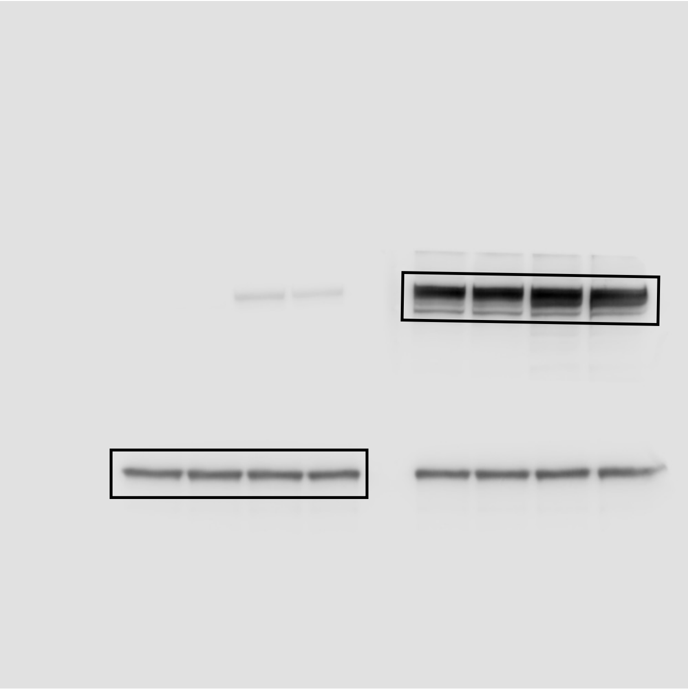

Fig. 6A (right panel), P-PKD S916

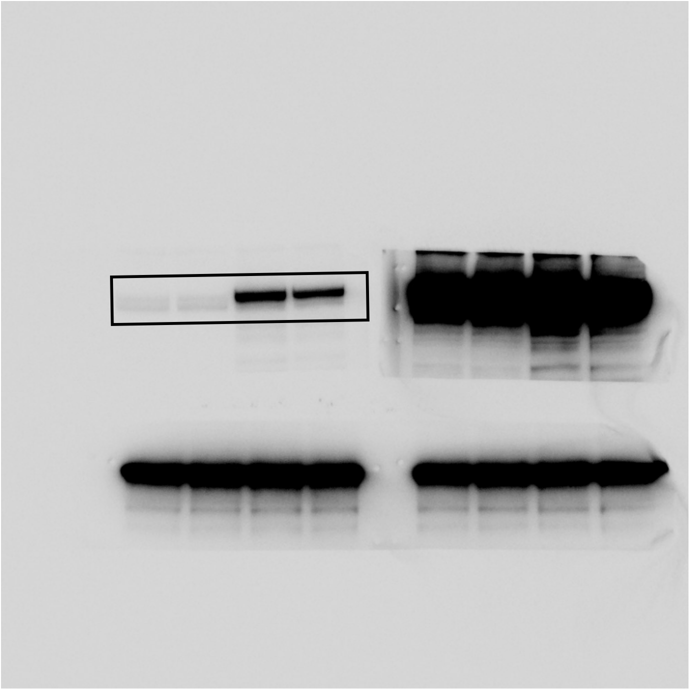

Fig. 6B, msPINK1

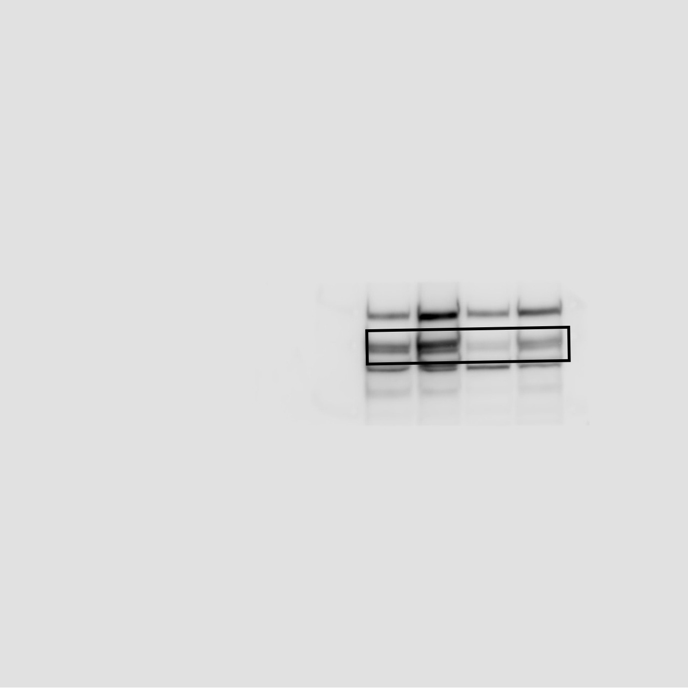

Fig. 6B, mcParkin

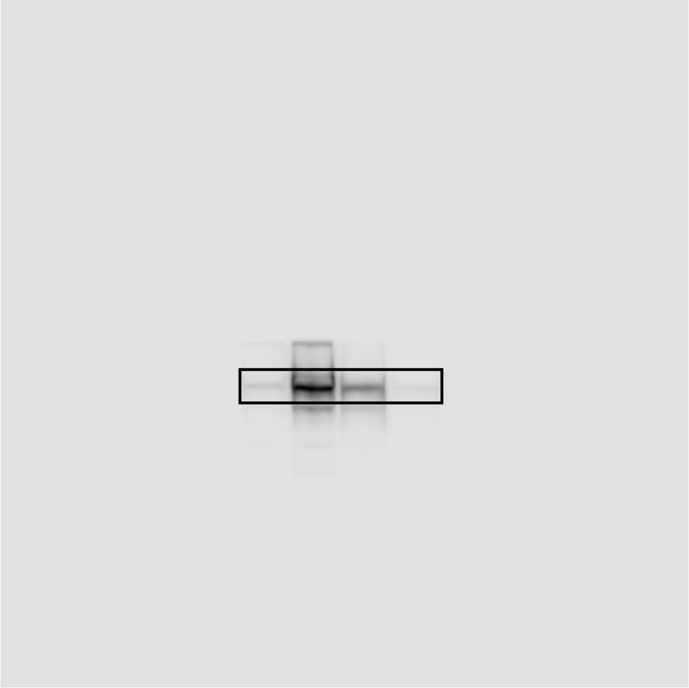

Fig. 6B, VDAC

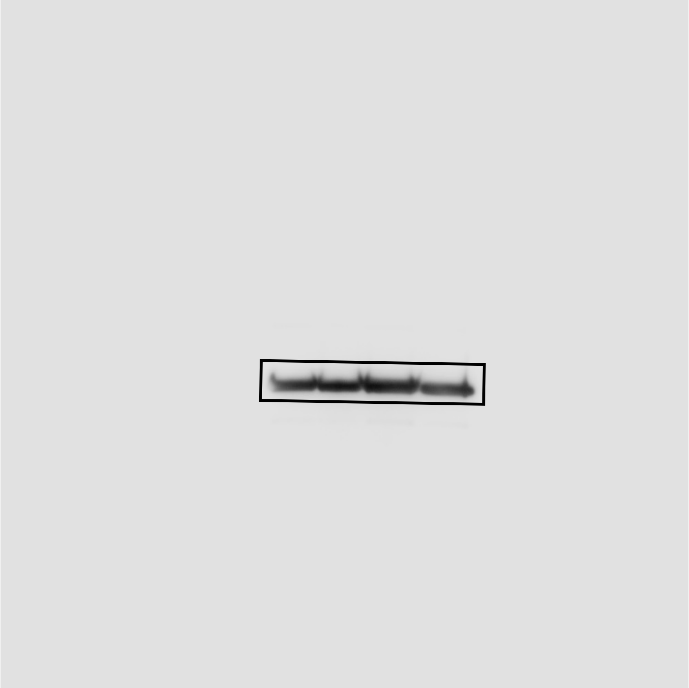

Fig. 6C, PINK1 (upper) and Parkin (lower)

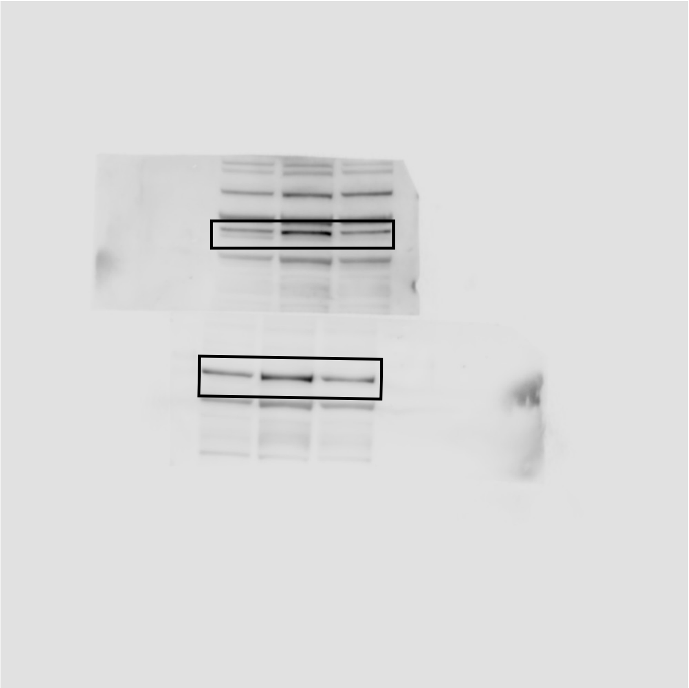

Fig. 6C, COX-IV (upper) and VDAC (lower)

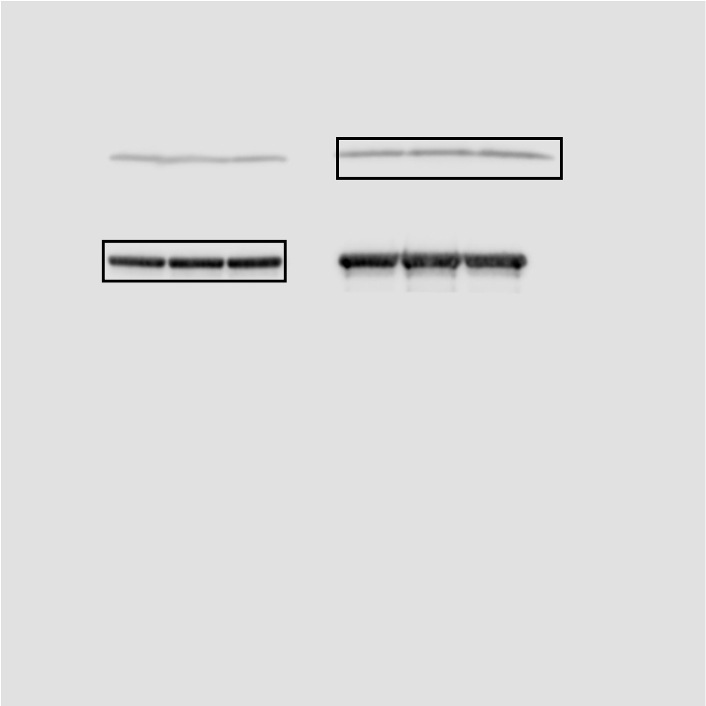

Fig. 6C, Ub

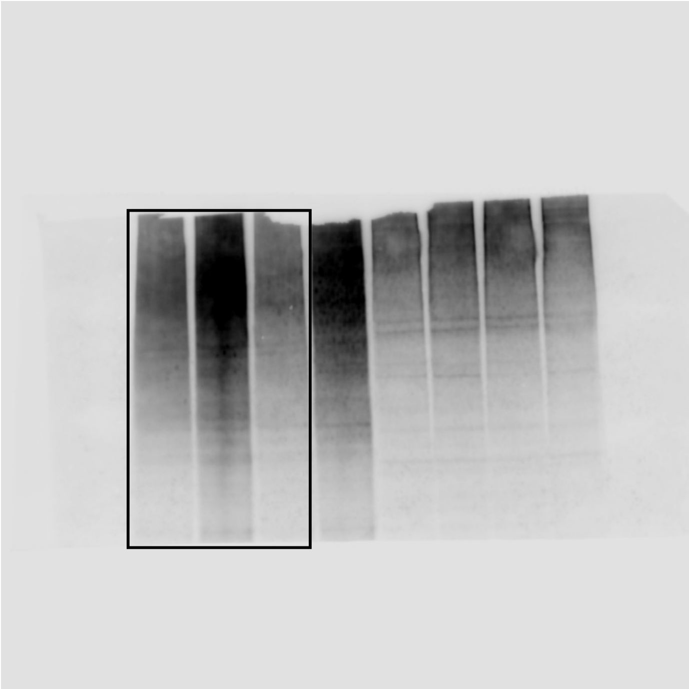

Fig. 6C, COX-IV for Ub

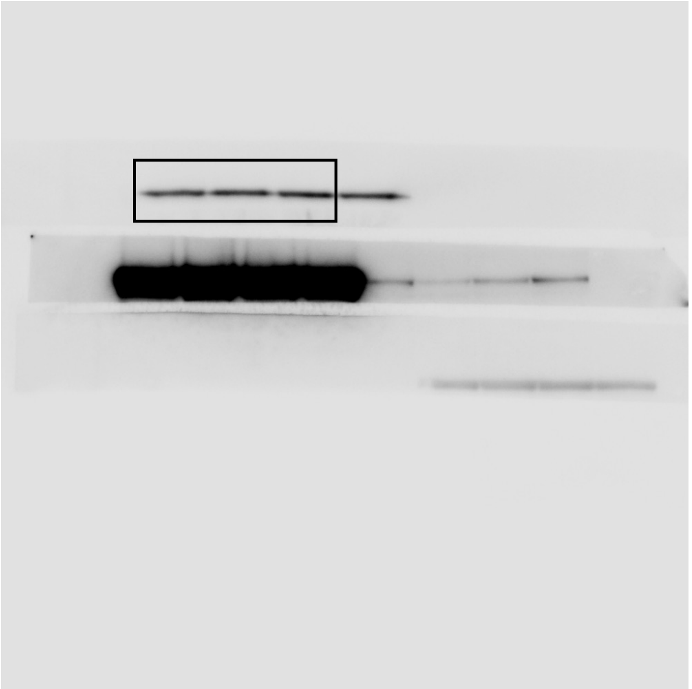

Fig. 6D (left panel), P-PKD S916

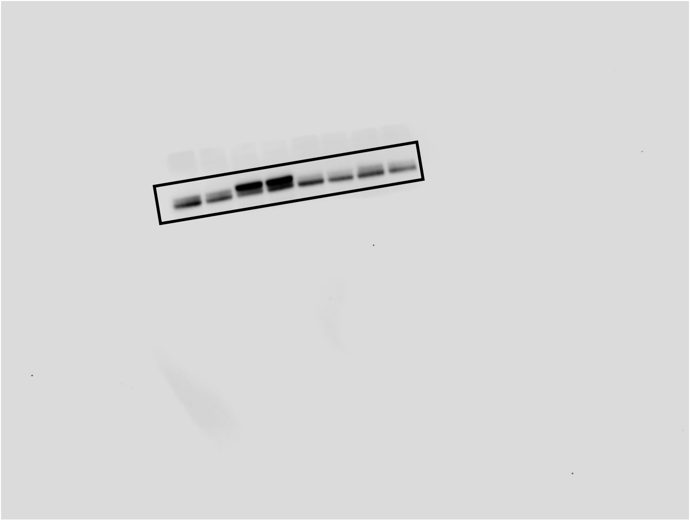

Fig. 6D (left panel), Total PKD

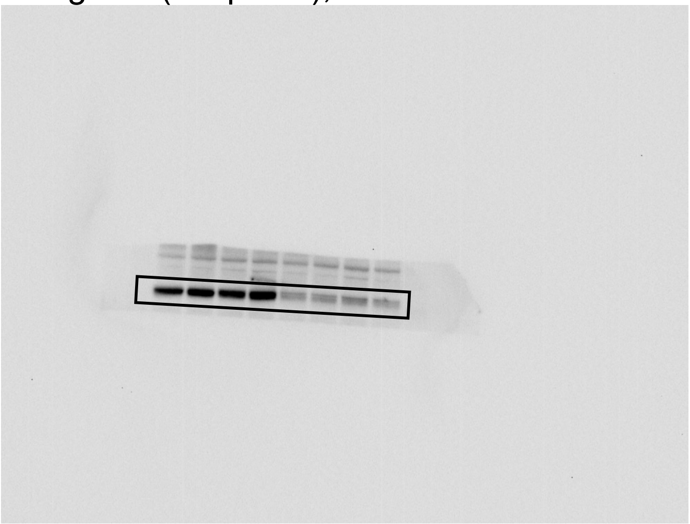

Fig. 6D (left panel), GAPDH

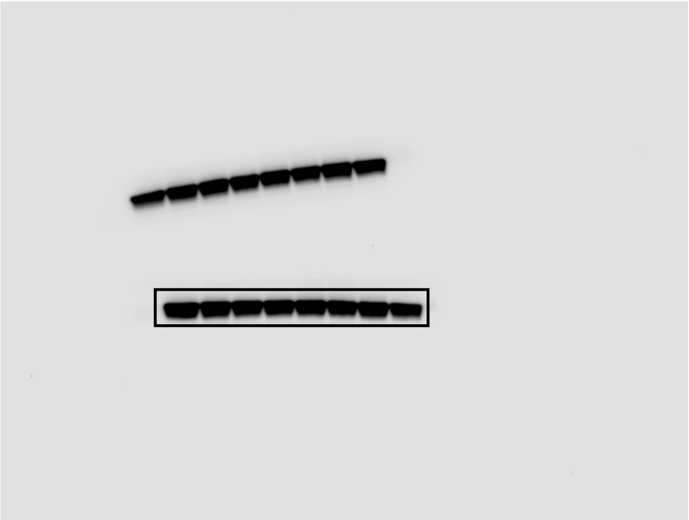

Fig. 6D (middle panel), PINK1

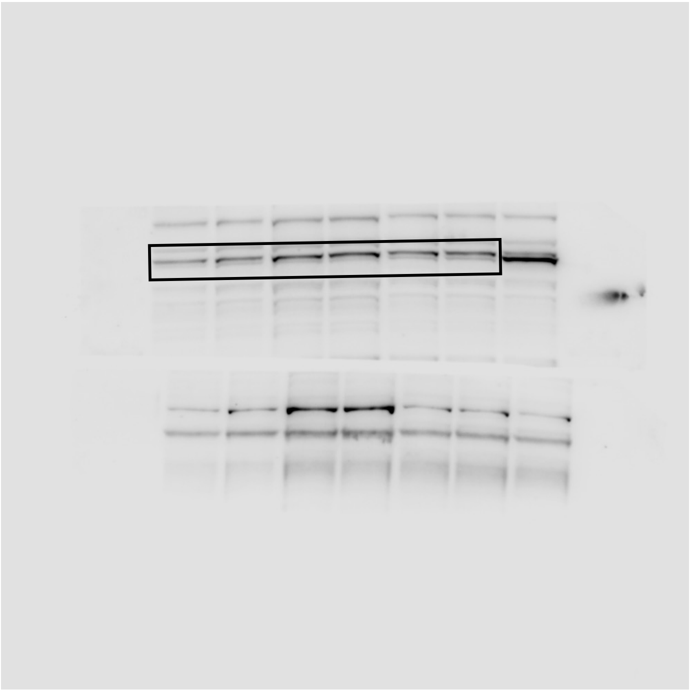

Fig. 6D (middle panel), COX-IV for PINK1

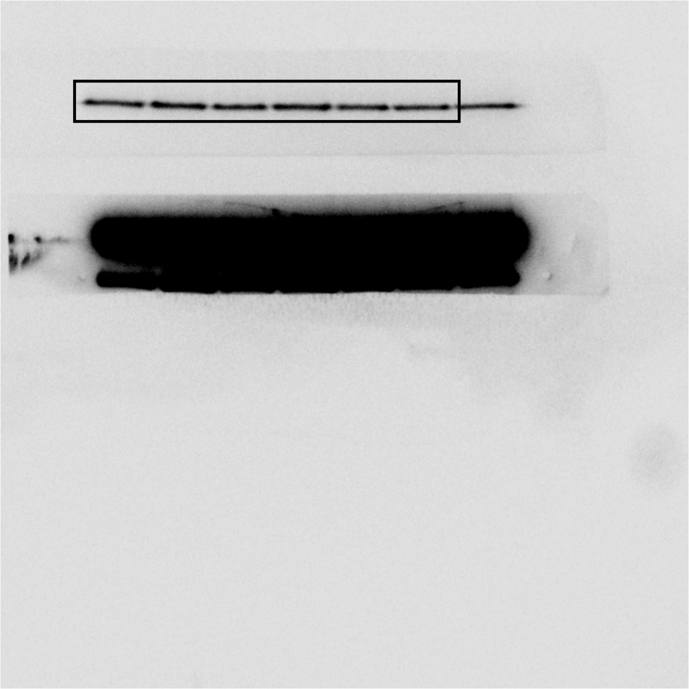

Fig. 6D (right panel), Parkin

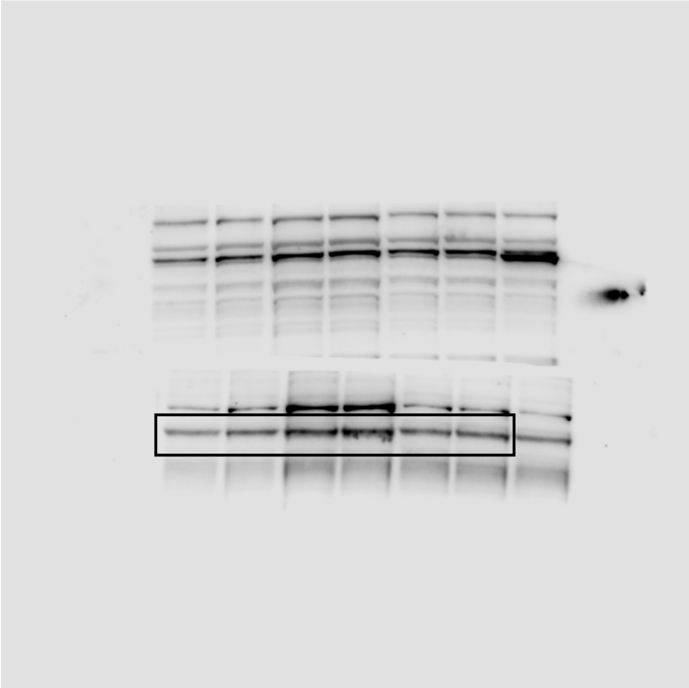

Fig. 6D (right panel), VDAC for Parkin

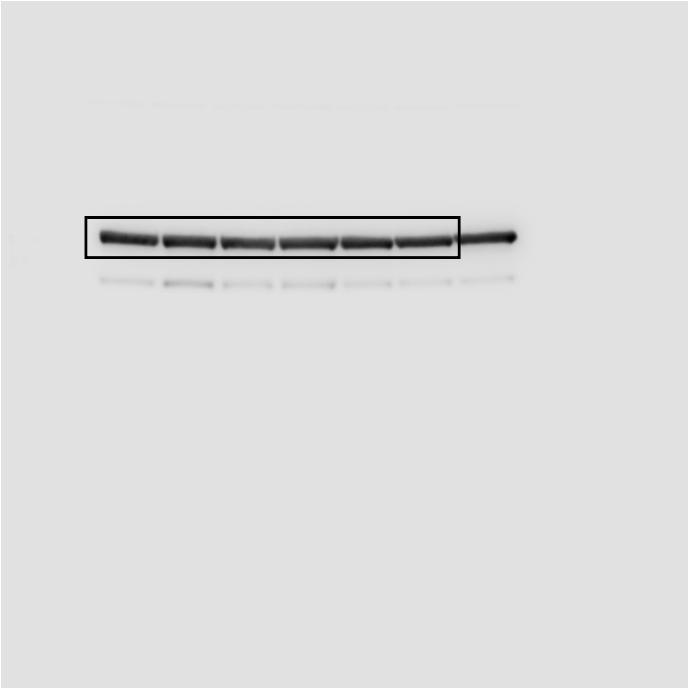

Fig. 7A, RhoA

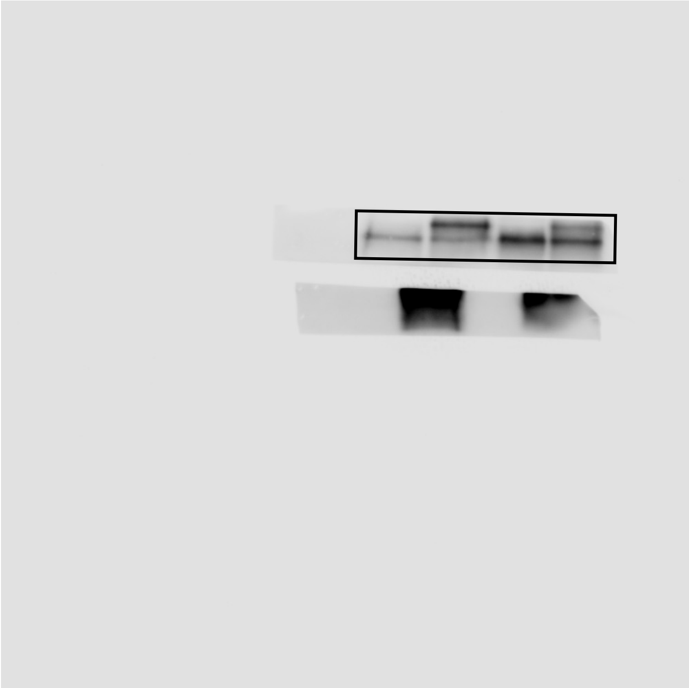

Fig. 7A, HA

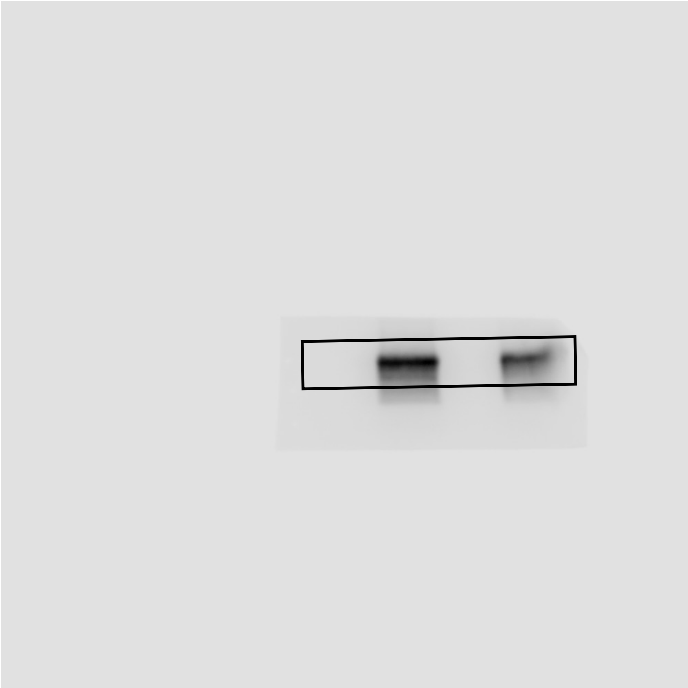

Fig. 7A, VDAC

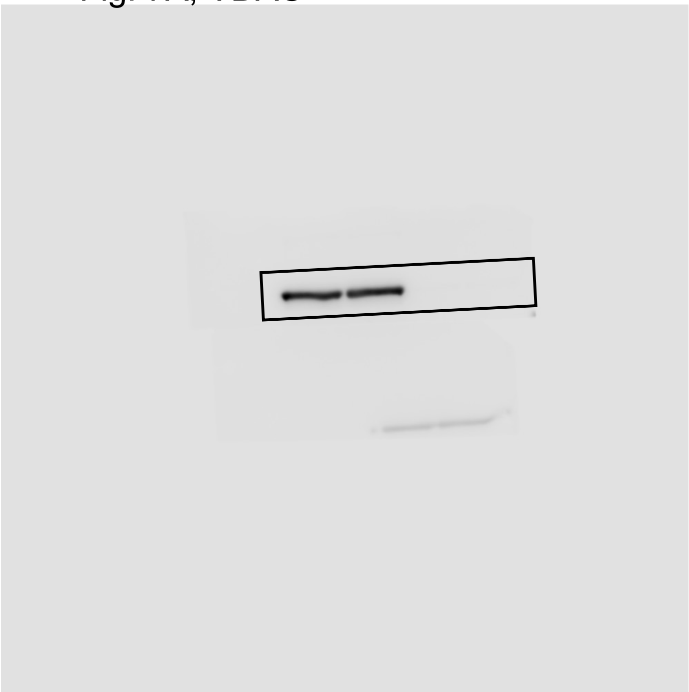

Fig. 7A, Rho-GDI

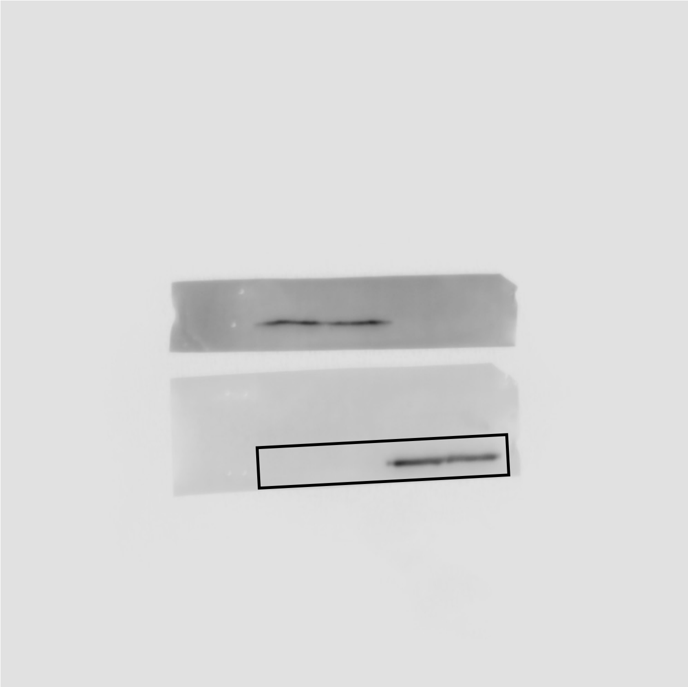

Fig. 7B, RhoA

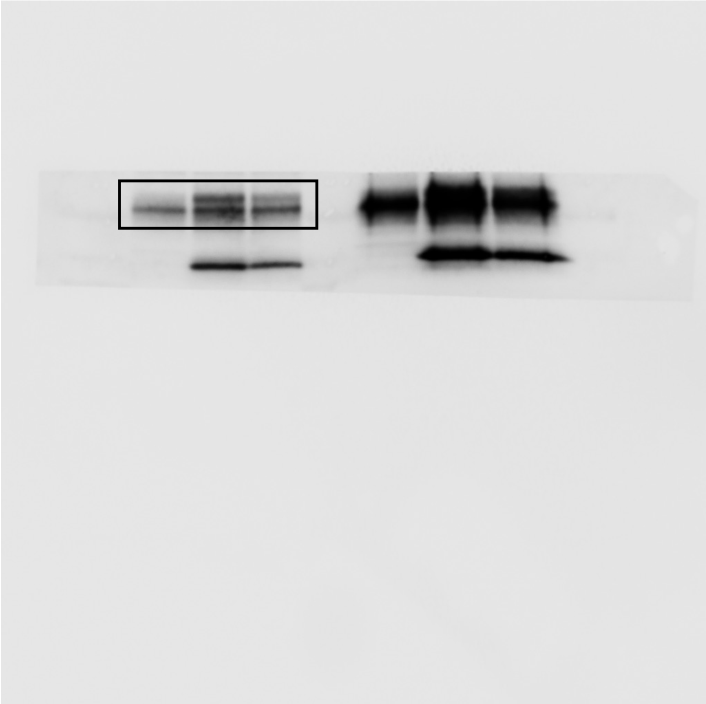

Fig. 7B, VDAC

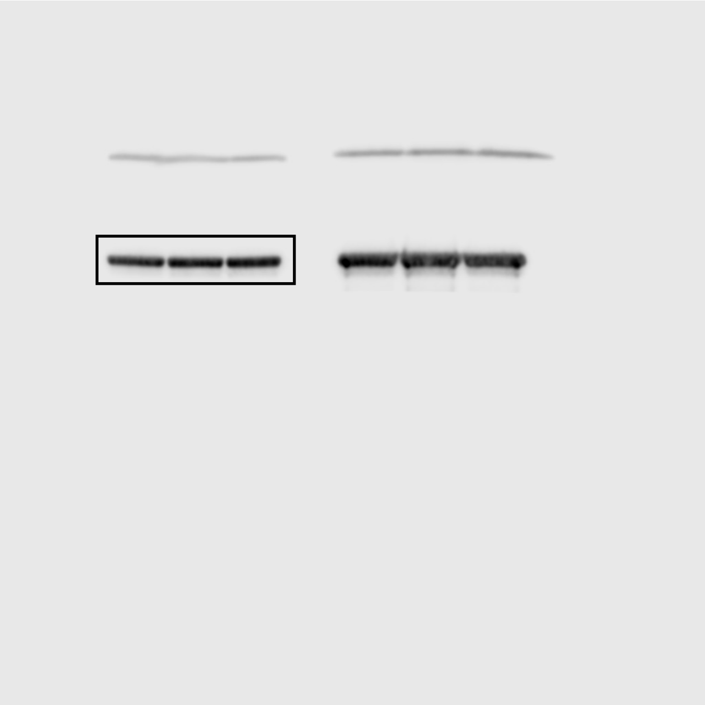

Fig. 7C, RhoA

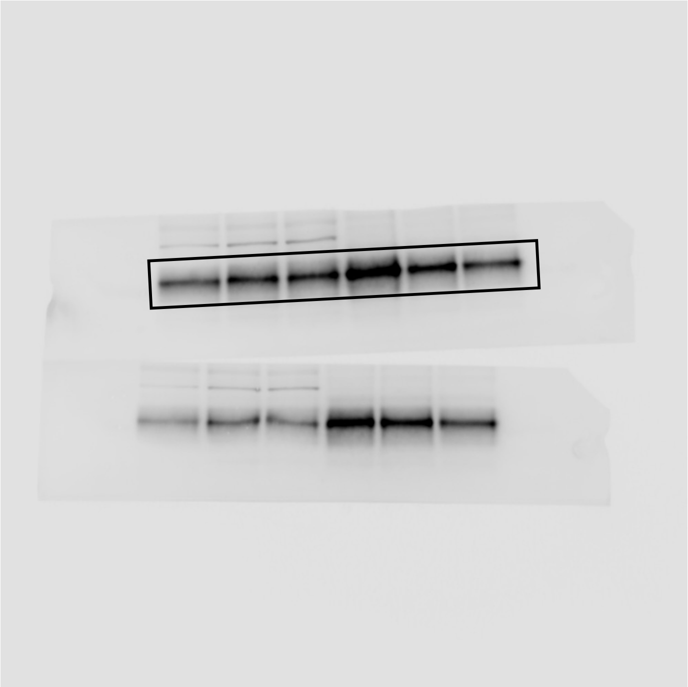

Fig. 7C, VDAC (upper) and Rho-GDI (lower)

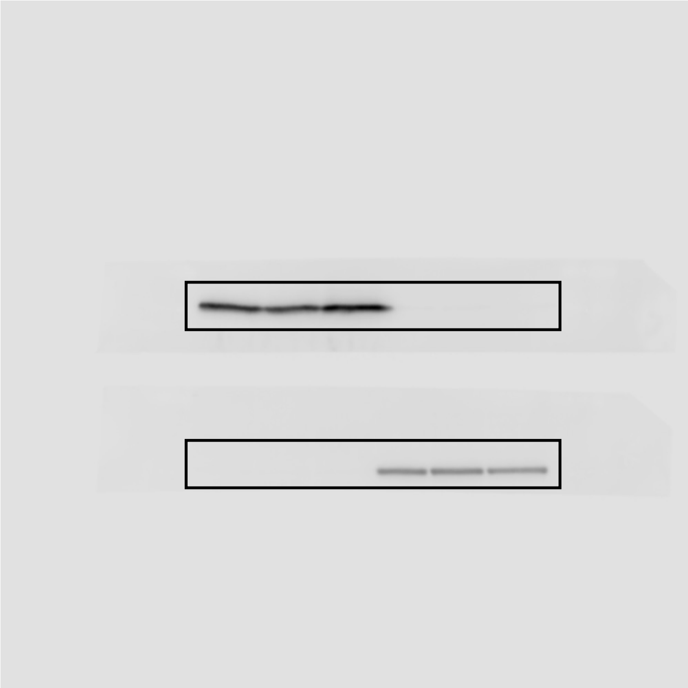

Fig. 7D, RhoA

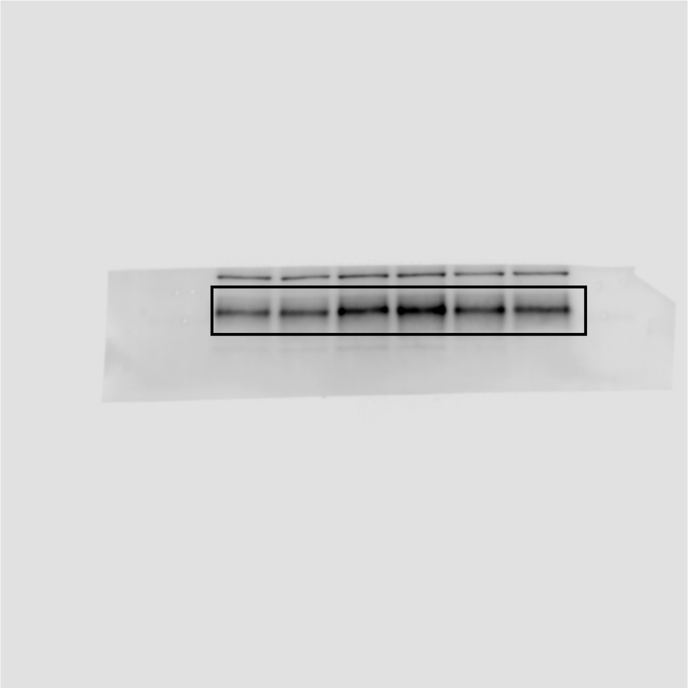

Fig. 7D, VDAC

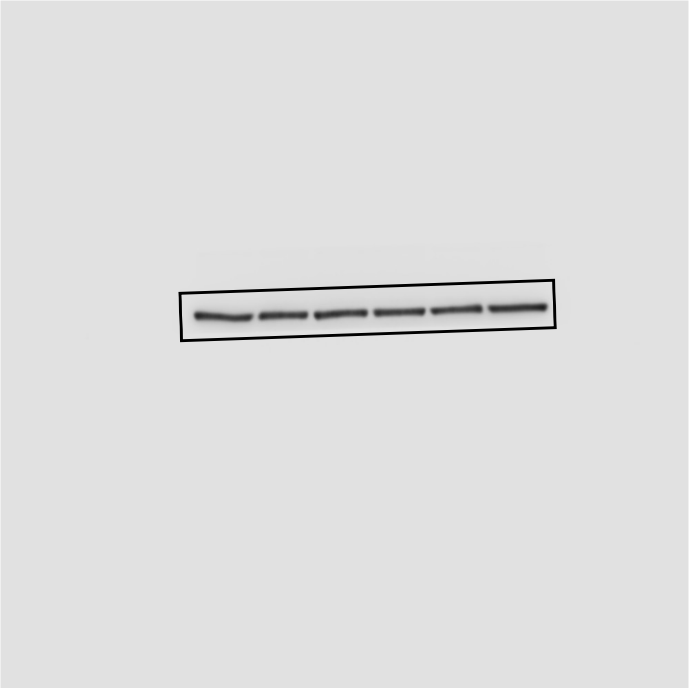

Fig. 7E, P-PKD

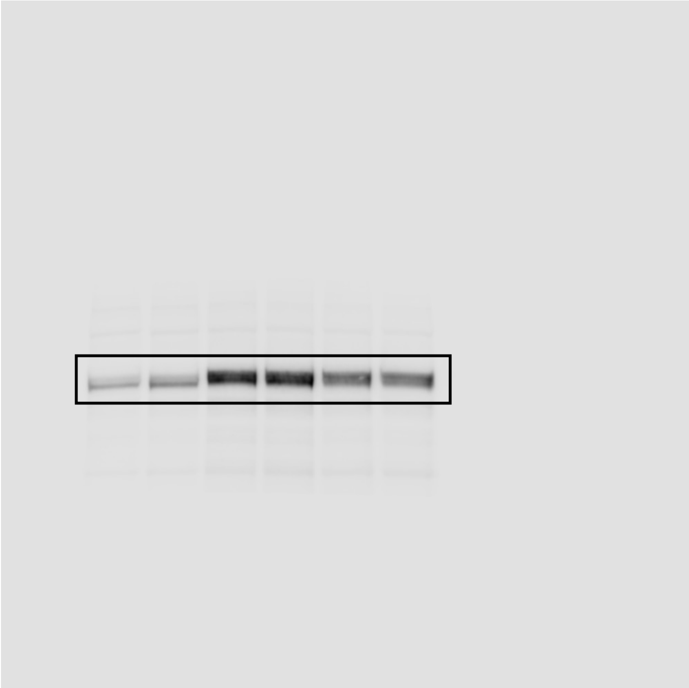

Fig. 7E, T-PKD

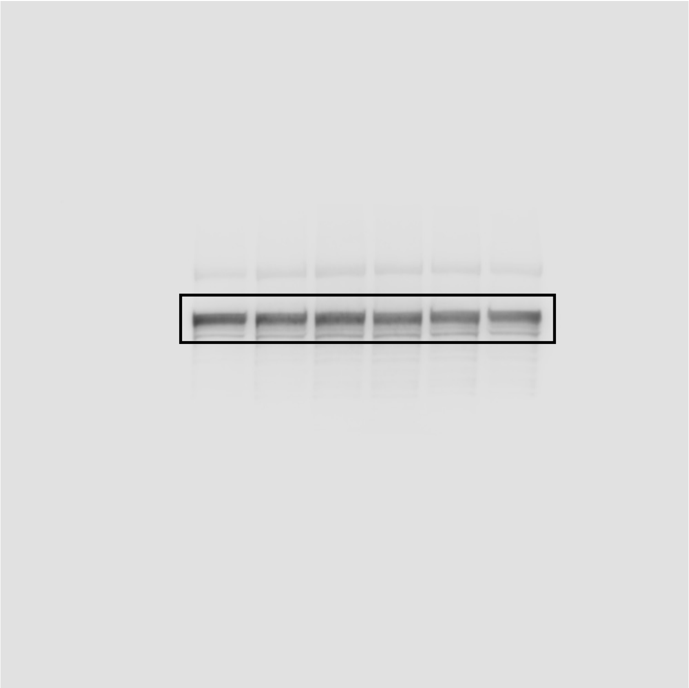

Fig. 7E, GAPDH

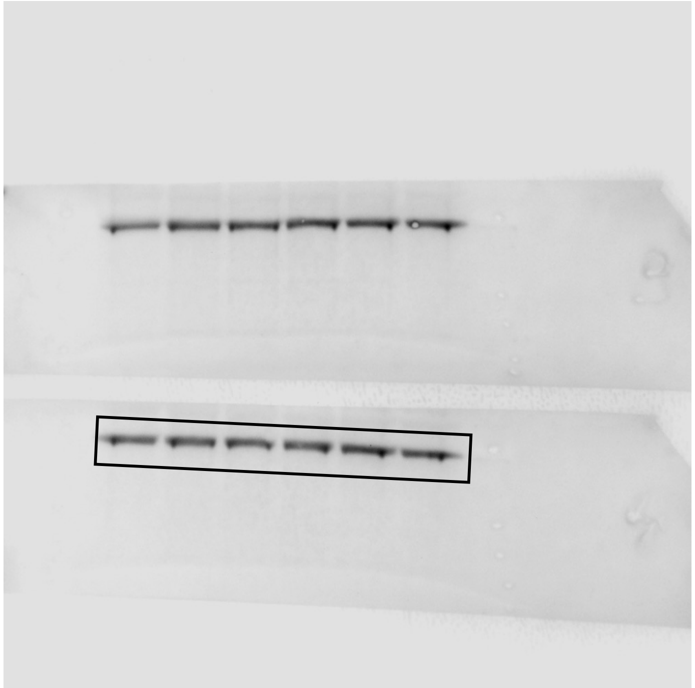

Fig. 8A, HA

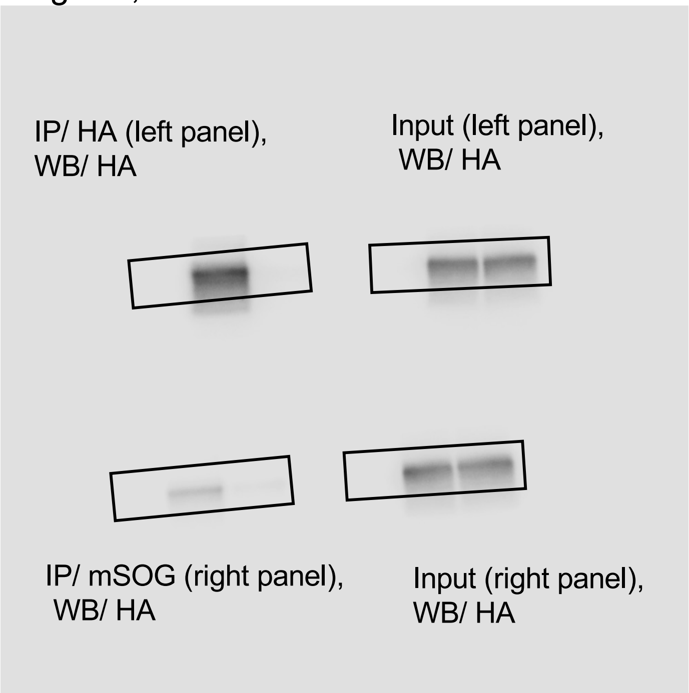

Fig. 8A, mSOG

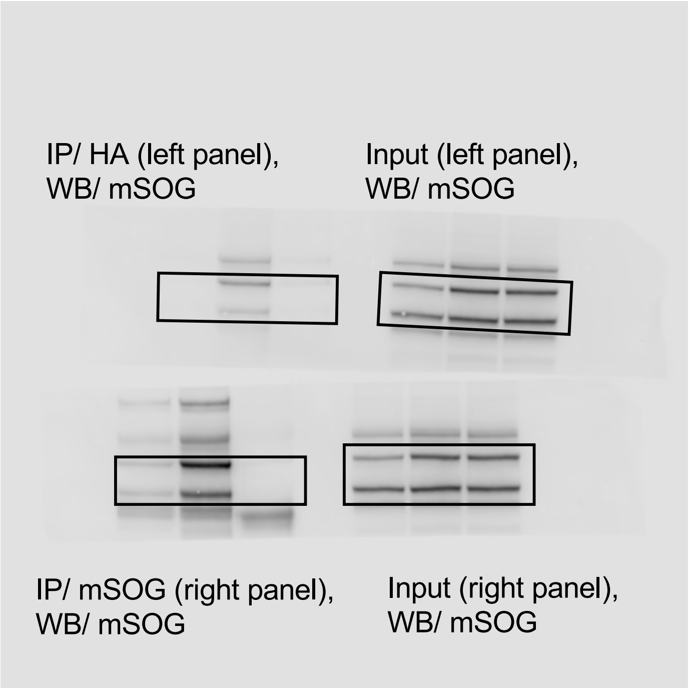

Fig. 8A, GAPDH (left panel)

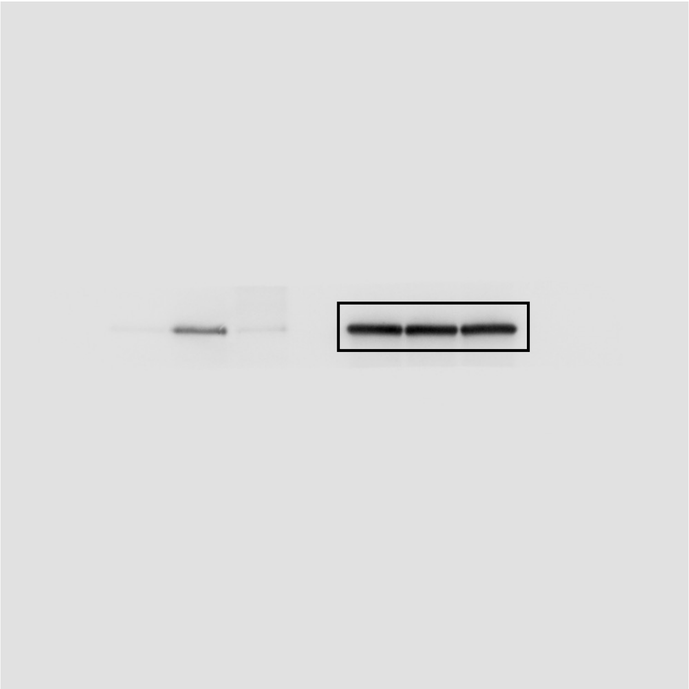

Fig. 8A, GAPDH (right panel)

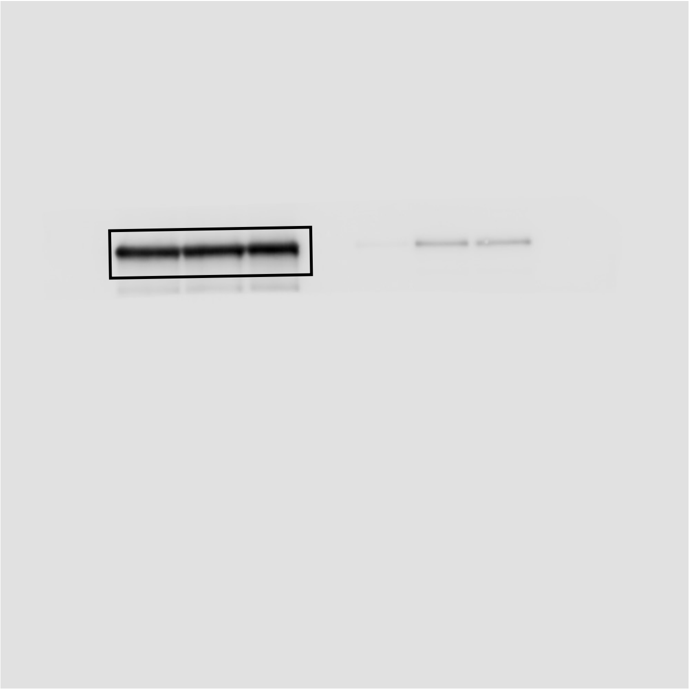

Fig. 8B, HA (IP/HA)

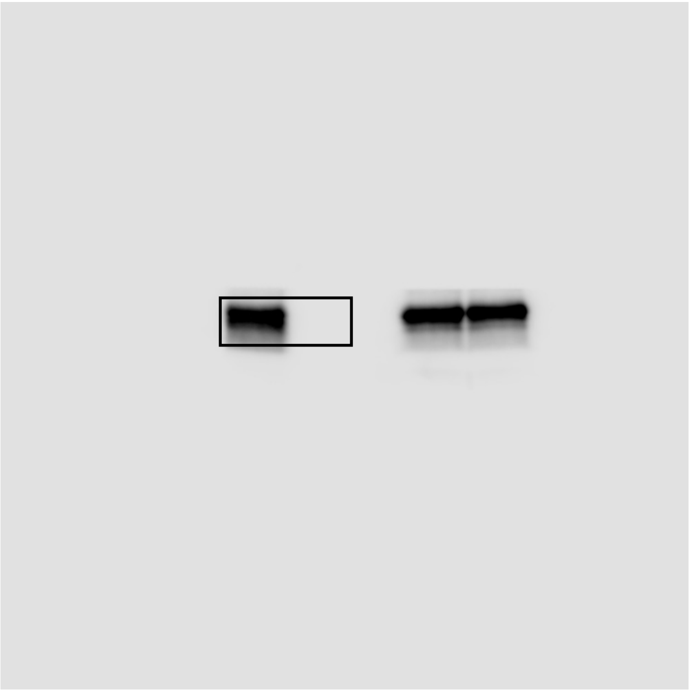

Fig. 8B, mSOG (IP/HA)

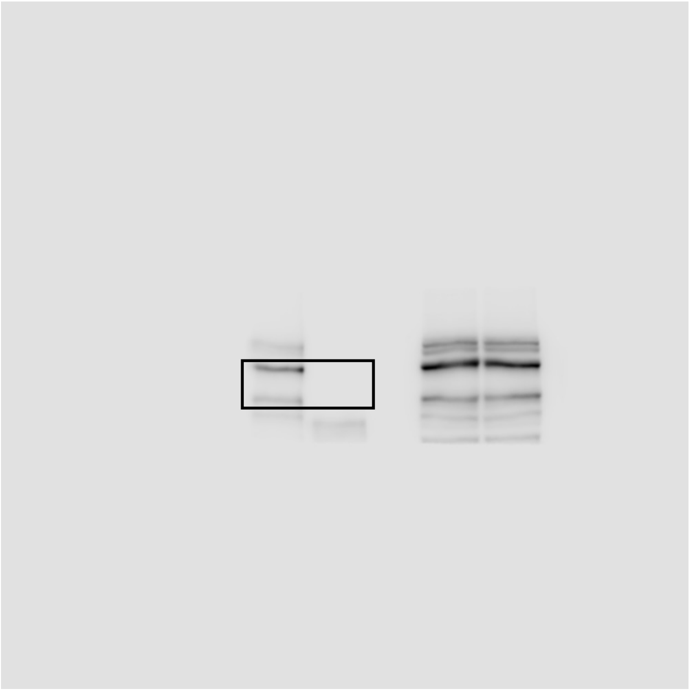

Fig. 8B, mSOG (IP/mSOG)

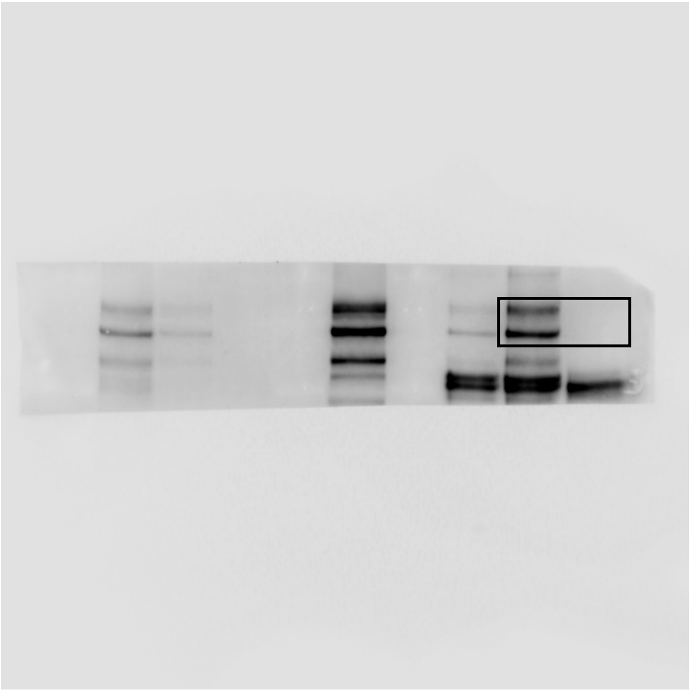

Fig. 8B, HA (IP/mSOG)

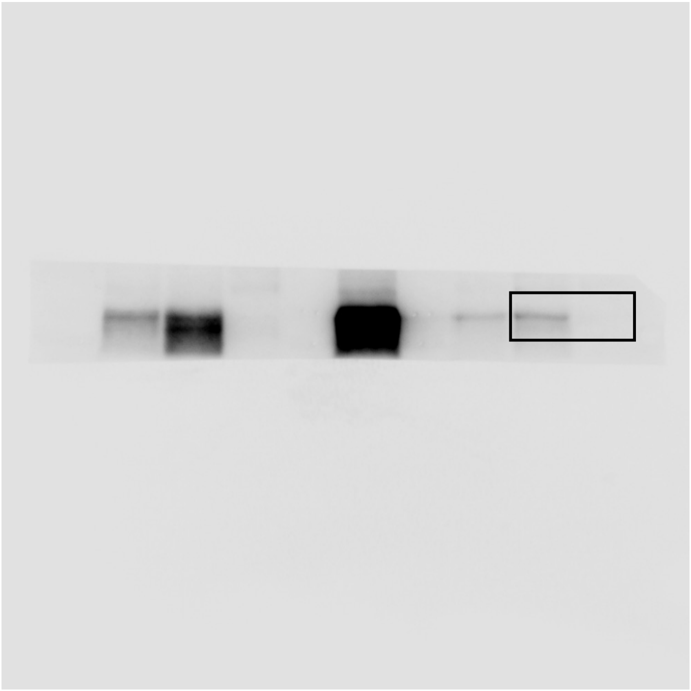

Fig. 8B, HA (input)

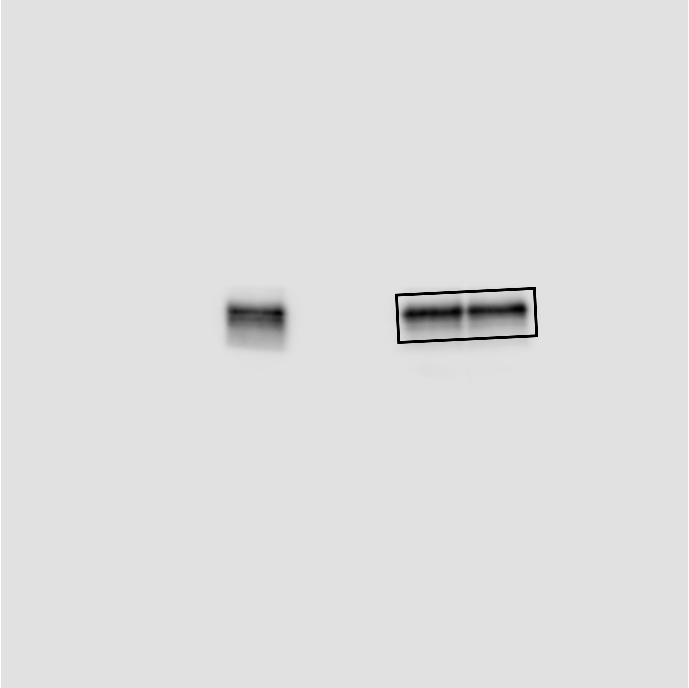

Fig. 8B, mSOG (input)

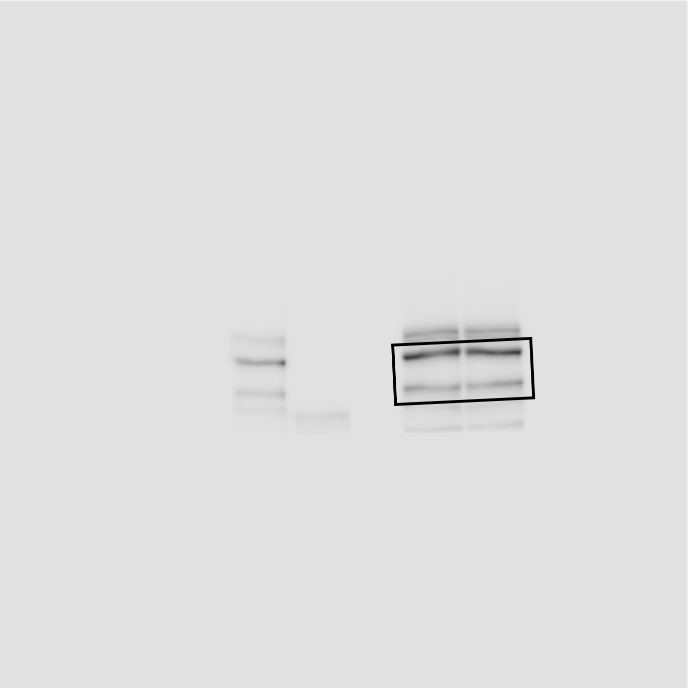

Fig. 8B, VDAC (input)

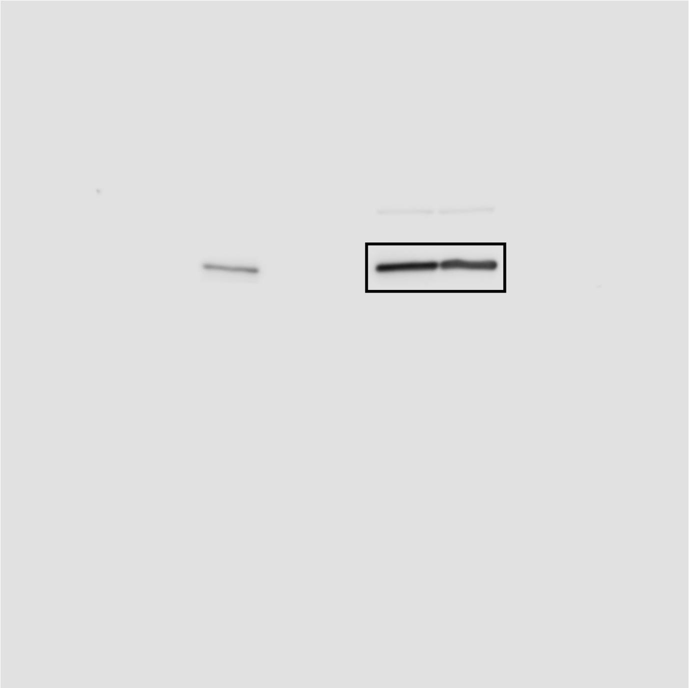

Fig. 8C, RhoA (IP/RhoA)

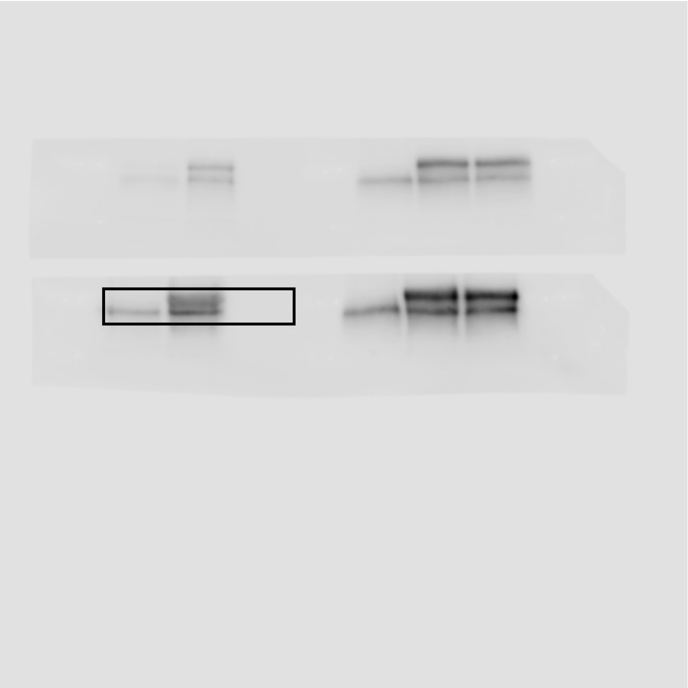

Fig. 8C, PINK1 (IP/RhoA)

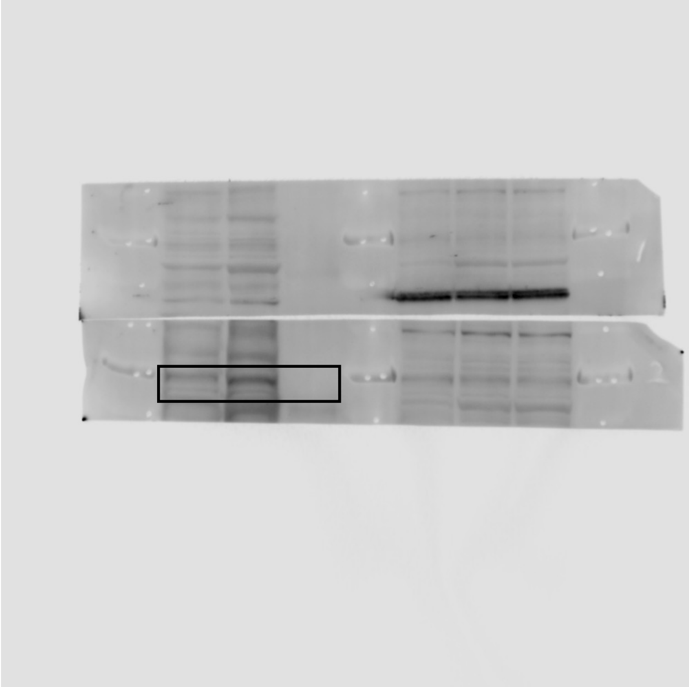

Fig. 8C, RhoA (input)

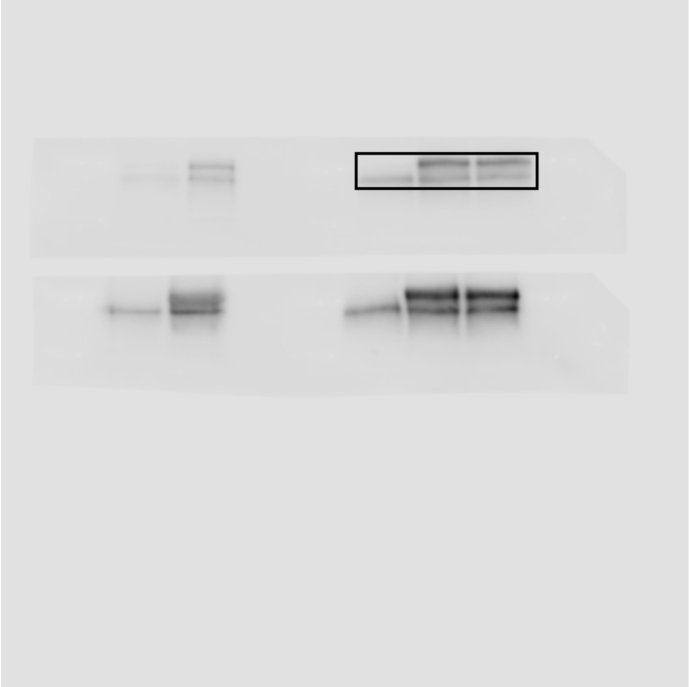

Fig. 8C, PINK1 (input)

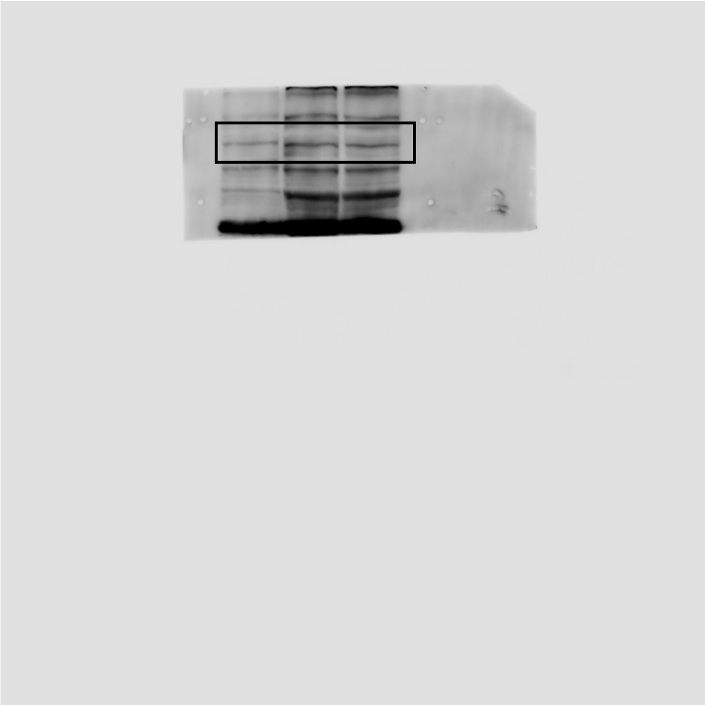

Fig. 8C, VDAC (input)

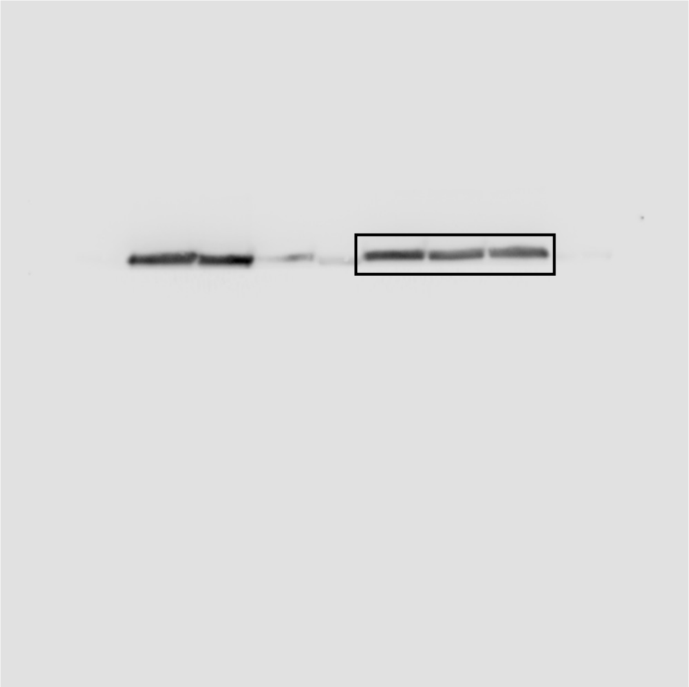

Suppl. Fig. 1, msPINK1 for RhoA

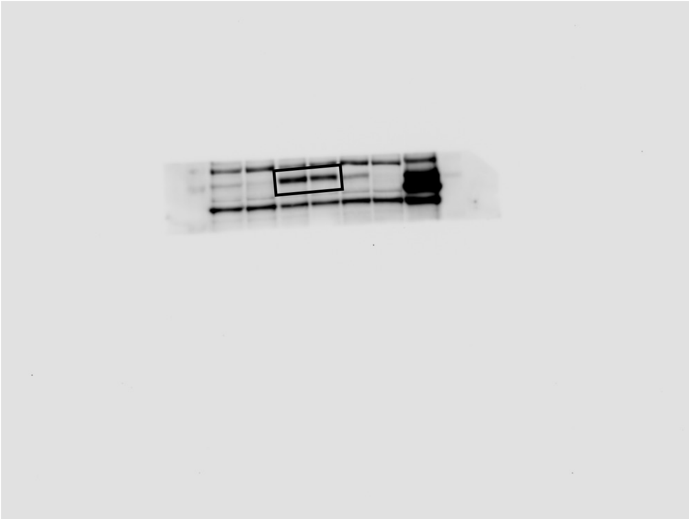

Suppl. Fig. 1, msPINK1 for PKD1

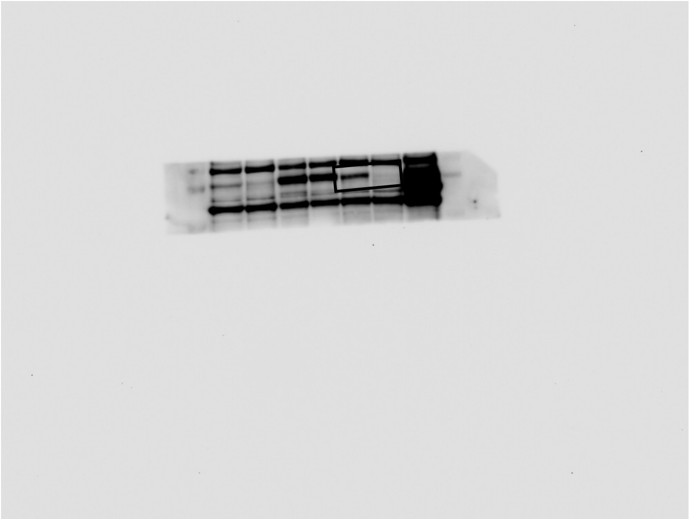

Suppl. Fig. 1, GAPDH for RhoA (left) and PKD (right)

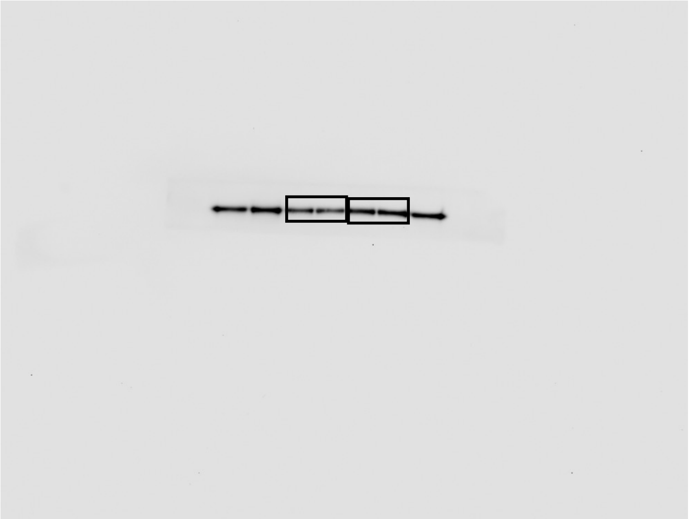

Suppl. Fig. 2, PINK1

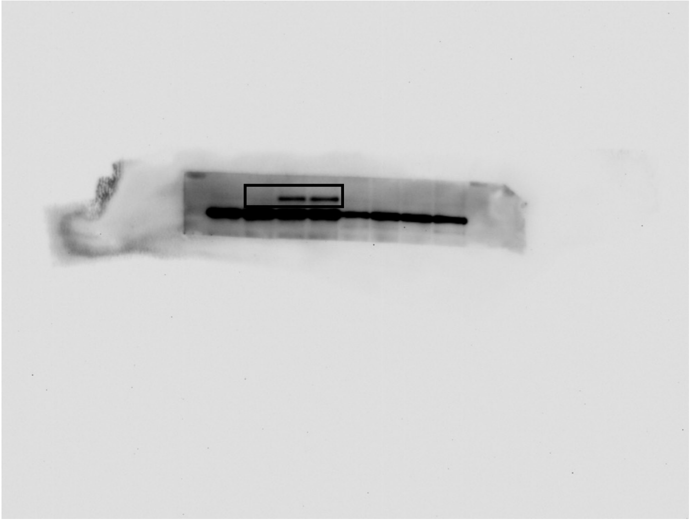

Suppl. Fig. 2, COX-IV

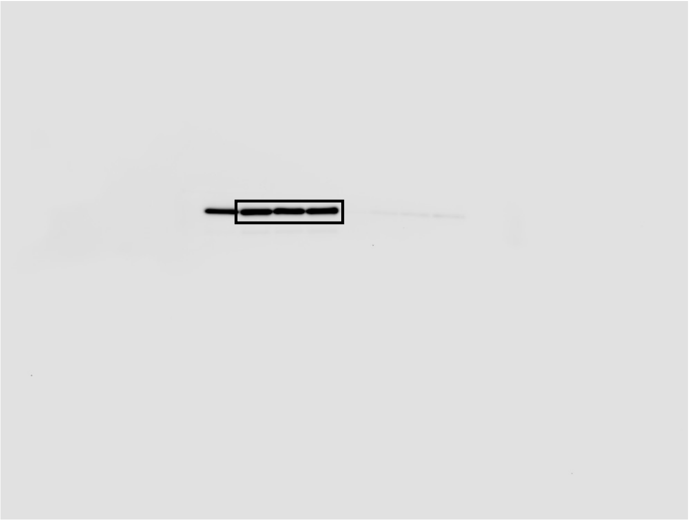

Supplement: Supplementary file 3 — Raw western blot images [file 41418_2022_1032_MOESM3_ESM.pdf]
