# Supplementary material for: RhoA signaling increases mitophagy and protects cardiomyocytes against ischemia by stabilizing PINK1 protein and recruiting Parkin to mitochondria
Source: Cell Death Differ. 2022 Jun 27;29(12):2472–86. doi: 10.1038/s41418-022-01032-w (PMC9751115; doi:10.1038/s41418-022-01032-w)
Supplement: Supplementary file 4 — Author Contribution Form [file 41418_2022_1032_MOESM4_ESM.pdf]

DECLARATION OF CONTRIBUTIONS TO ARTICLE

ADMC

Manuscript Number: CDD-21-2077

Journal Name: *Cell Death & Differentiation* (the 'Journal')

Proposed Title of the Contribution: RhoA signaling increases mitophagy and protects cardiomyocytes against ischemia (the 'Contribution)

Author(s): Michelle Tu, Valerie P. Tan, Justin D. Yu, Raghav Tripathi, Zahna Bigham, Melissa (the 'Authors')

For all *CDD* articles, each person named as an author in the published version must be able to show he or she has contributed substantially to the article.

Authorship credit should be based on 1) substantial contributions to conception and design, acquisition of data, or analysis and interpretation of data; 2) drafting the article or revising it critically for important intellectual content; and 3) final approval of the version to be published. Authors should meet conditions 1, 2 and 3.

Any person who cannot be shown to have made a substantial contribution to the article cannot be listed as an author in the final version. The name of any person who is deemed to have made a minor contribution can, however, appear in the Acknowledgments section of the article.

Please complete the table below to indicate the contributions of all named authors to the manuscript.

Author Full Name:	Specification of Contribution to the Manuscript:
Michelle Tu	acquisition of data, analysis and interpretation of data, generation
Valerie P. Tan	acquisition of data, analysis and interpretation of data, generation
Justin D. Yu	acquisition of data, analysis and interpretation of data, editing the
Raghav Tripathi	acquisition of data, analysis and interpretation of data, generation
Zahna Bigham	acquisition of data, analysis and interpretation of data, generation
Melissa Barlow	acquisition of data, editing the manuscript.
Jeffrey M. Smith	acquisition of data, analysis and interpretation of data, editing the
Joan Heller Brown	conceptualization and designing the study, writing and editing the
Shigeki Miyamoto	conceptualization and designing the study, acquisition of data,

Please complete the table below to indicate the contributions of all named authors to the figures.

Figure 1:
Michelle Tu, Valerie P. Tan, Justin D. and Shigeki Miyamoto

Figure 2:
Michelle Tu, Valerie P. Tan, Justin D. Yu, and Shigeki Miyamoto

Figure 3:
Michelle Tu, Valerie P. Tan, Melissa Barlow, Jeffrey M. Smith and Shigeki Miyamoto

Figure 4:
Valerie P. Tan, Melissa Barlow, Jeffrey M. Smith, and Shigeki Miyamoto

Figure 5:
Michelle Tu, Justin D. Yu, Raghav Tripathi, Zahna Bigham and Shigeki Miyamoto

Figure 6:
Figure6: Raghav Valerie P. Tan, Tripathi, Zahna Bigham and Shigeki Miyamoto.
Figure 7: Michelle Tu, Valerie P. Tan Justin D. Yu, Raghav Tripathi, Zahna Bigham and Shigeki Miyamoto
Figure8: Michelle Tu, Justin D. Yu and Shigeki Miyamoto

Signed for and on behalf of the Author(s):	Print Name:	Date:
Shigeki Miyamoto	Shigeki Miyamoto	04/30/2022
