# Supplementary figures and images for: RhoA signaling increases mitophagy and protects cardiomyocytes against ischemia by stabilizing PINK1 protein and recruiting Parkin to mitochondria
Source: Cell Death Differ. 2022 Jun 27;29(12):2472–86. doi: 10.1038/s41418-022-01032-w (PMC9751115; doi:10.1038/s41418-022-01032-w)

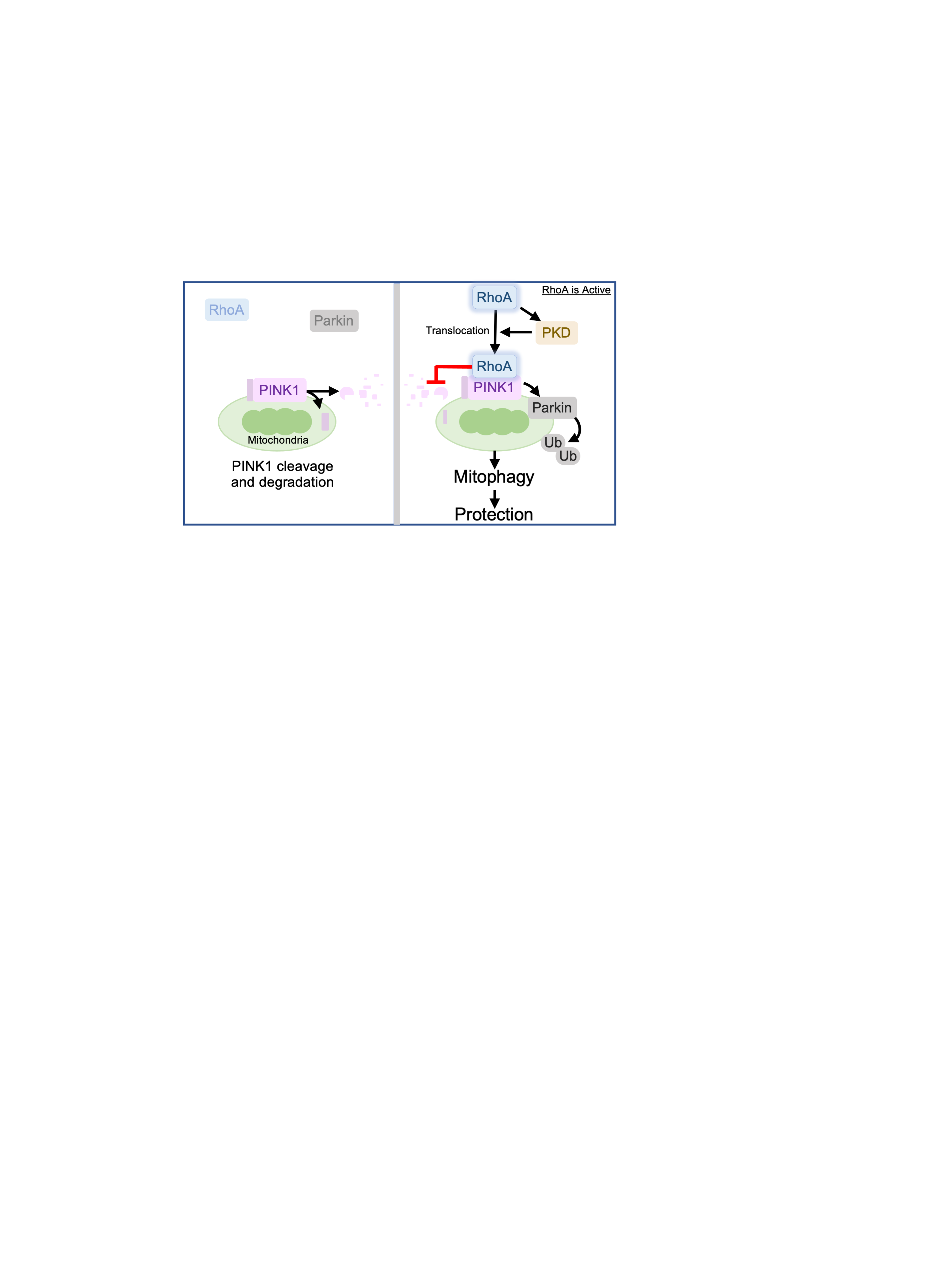

Supplement: Supplementary file 6 — Related Manuscript File [file 41418_2022_1032_MOESM6_ESM.tif]
